# A qualitative examination of football players’ acceptability and perceptions on the use of virtual reality in football training

**DOI:** 10.1371/journal.pone.0334167

**Published:** 2025-10-09

**Authors:** Anna U. Shields, David L. Neumann, Matthew J. Stainer

**Affiliations:** School of Applied Psychology, Griffith University, Gold Coast, Queensland, Australia; Manchester Metropolitan University Faculty of Health and Education, UNITED KINGDOM OF GREAT BRITAIN AND NORTHERN IRELAND

## Abstract

**Introduction:**

Virtual reality (VR) technologies are being increasingly used for training among football teams. Despite this, limited research has examined football players’ acceptability and perceptions of VR training. It is essential to understand football players’ perspectives on this training approach to identify any psychological factors that may influence its uptake.

**Methods:**

A qualitative approach was adopted to explore footballers’ acceptability and perceptions of VR in training. Fourteen professional and semi-professional football players completed a survey and took part in qualitative semi-structured interviews between May and October 2023. All participants were asked questions about their perspectives of the benefits and barriers of using VR for football training. The interview data was recorded and analysed using reflexive thematic analysis.

**Results:**

Themes regarding the footballers’ acceptability were interpreted within the framework of the Technology Acceptance Model. Overall, the footballers demonstrated acceptability and emphasised its potential usefulness in football training. Footballers perceived VR as being beneficial for cognitive and perceptual-motor skill training, mental preparation and pressure training through exposure to representative game-like environments, as well as providing greater training flexibility. Alongside these benefits, factors which could hinder optimal uptake were identified, including lack of accessibility, financial costs, and perceptual differences between the VR environments and the real-world.

**Conclusion:**

The present study provides insight into experienced football players’ acceptability and perceptions of VR for training and suggest that VR training has potential to be accepted by football players, particularly when it is promoted to them as a flexible approach that can enhance their on-field performance.

## Introduction

Virtual reality (VR) refers to computer-simulated, interactive environments that aim to create the experience of being physically and psychologically present in another place and enables the user to interact with features of the virtual environment [1]. The technology offers multiple opportunities for sports training through simulation of real-world task and environmental constraints, enabling creation of controlled and repeatable training tasks that may be impractical and impossible to replicate in real-world training. Football teams have used VR to train players’ cognitive skills such as scanning, decision-making, and situational awareness through simulations that replicate real-world football match scenarios [2], and as a training aid to keep injured players mentally engaged in football without the physical load of on-field training [3]. However, to date no studies have examined football players’ perspectives on using VR in football training before first use. Understanding how footballers view VR training before first use is essential, as an individual’s initial attitudes and perceptions can strongly influence the adoption of new technological systems [4]. As such, the present study aimed to explore the acceptability and perceptions regarding the use of VR as a complementary training tool in a sample of professional and semi-professional football players’ who have not previously used VR for training.

VR technologies afford various opportunities in football training. By simulating real-life game environments, VR may serve as an effective training tool to enhance perceptual-cognitive skills (e.g., decision-making, anticipation) [5], and perceptual-motor skills (e.g., timing of actions, reaction speed) [6] that are crucial to achieve high-level performance in football. Empirical research has demonstrated that computer-generated VR heading training improves football players’ real-world heading performance [7], and decision-making and visual search behaviours [8]. Additionally, VR training may enhance players ability to better cope with psychological pressure and build psychological resilience through exposure to representative high-pressure scenarios [9,10]. VR simulations may also aid in injury prevention and rehabilitation by allowing players to engage in football-related actions without the physical strain of real-world training, enabling them to continue to train cognitively, even when injured [11]. Further, VR training may enhance footballers’ perceptions of self-efficacy and confidence in their abilities which may enhance their performance on-field [7], Overall, VR presents a range of opportunities to be used as a complement to real-world training.

However, it is important to note that VR has limitations and is best considered as a supplementary training tool to real-world training. For example, VR environments cannot fully replicate the physical and sensory demands of football, such as the tactile interaction with a physical ball [12]. Similarly, while VR can be useful for enhancing perceptual-cognitive, psychological, and more simple motor skills (e.g., golf putting), the technology may not be effective for complex motor skill development (e.g., tackling) due to the lack of physical interaction [6]. In addition, VR cannot replicate the real-time interactions between players that are essential for tactical awareness and co-adaptive decision-making that emerges through the dynamic interplay of teammates and opponents [13]. Moreover, there are practical factors which may hinder implementation of this technology, including financial costs, time constraints, and technical constraints [11,14,15].

Despite the potential applications of VR technologies for football training, limited research has examined footballers’ perspectives on VR for training. A greater understanding of football players’ acceptability and perceptions of factors which could limit uptake of VR in training is essential because an individual’s attitudes and beliefs towards a technology can either facilitate or hinder their willingness and subsequent behavioural intentions to adopt it [4,16]. In fact, even if a technology has been found to be effective, if it is not perceived as useful, or accepted by its intended user, it might not be adopted or optimally used [4].

User acceptance of a technology is the willingness to employ it for the task it is intended for [17], and is critical for successful adoption of a technology [4]. Acceptance is divided into two processes: acceptance before using a technological device, known as *acceptability*, and acceptance after use, referred to as *acceptance* [18]. Acceptability is an a priori phenomenon, and refers to a more or less positive mental representation of a technology before having used it, while acceptance is an posteriori pragmatic evaluation, and refers to an individual’s judgment towards a technology after actual use [18]. Both acceptability and acceptance are crucial determinants of initial and continued use of a technology [18]. The present study focuses on acceptability, as individuals may hold beliefs of a technology before first use based on prior knowledge, external influences (e.g., perceptions among peers or coaches), and perceived relevance to its intended purpose [16]. Furthermore, given that the evidence supporting the effectiveness of VR for football training is still developing, and there is limited evidence that it is currently being used in applied training settings, it is likely that adoption among players will be low. Those who have not used VR may view it with scepticism, and examining acceptability may help identify psychological factors that could limit initial adoption of the technology [19].

Various theoretical frameworks have been used to investigate technology acceptance, including the Technology Acceptance Model [TAM; 4, 16] and the Unified Theory of Acceptance and Use of Technology [20]. The TAM is a widely applied theoretical framework for explaining user acceptance and intentions to use a new technology prior to first use, and following regular use in various domains (e.g., education, healthcare, sports training), using different technologies (e.g., smartphone apps, wearable technologies, artificial intelligence), and in different demographic populations (e.g., students, sport coaches, athletes, health professionals) [19,21–24]. The TAM suggests that perceived usefulness (i.e., perceived evaluation of a technology’s ability to improve performance) and perceived ease of use (i.e., the amount of effort required to use the technology) predict an individual’s attitudes towards a technology, which subsequently predict intentions to use and actual usage of the technology [4]. Additionally, perceived usefulness has consistently been found to be the strongest predictor of intentions to use [16]. As such, football players who perceive VR training as useful and easy to use are more likely to hold positive attitudes and have stronger intentions to adopt this training approach.

Although the TAM provides a framework for evaluating the acceptability of using VR for football training, it may not fully account for the broader range of practical and experiential factors that might influence footballers’ engagement with the technology, such as performance advantages, technical concerns, or contextual limitations [25]. As such, the present study also examined footballers’ perceptions of the benefits and barriers of using VR to provide complementary insights regarding real-world value and potential challenges associated with future use. The inclusion of acceptability and perceptions in the current study thus allowed for a comprehensive understanding that encompassed both theoretical willingness to adopt and practical considerations that might influence footballers use of VR for training.

Mascret et al. [19] examined acceptability of using VR for training among 1162 athletes of various sports disciplines and levels (ranging from recreational to international). The athletes completed a self-report survey assessing their perceived usefulness, ease of use, enjoyment, subjective norms, and intentions to use VR. The findings showed that athletes of all levels had significant intentions to use VR, and perceived VR to be easy to use and useful (except for recreational athletes). There were 138 football players included in the study, and examination of the descriptive statistics revealed that they had similar scores of perceived ease of use and perceived enjoyment, and higher scores of perceived usefulness, subjective norms, and intentions to use VR when compared to the general sample of athletes. No initial factors which could threaten intentions to use were identified among the athletes. A recent qualitative investigation by Lewellen et al. [15] examined experienced athletes’ perceptions on VR after using the technology for training, although no football players were included in their sample. Athletes were generally accepting about the use of VR training and viewed it as useful for perceptual-cognitive skill training, mental preparation, and enhancing confidence. The athletes noted barriers such as financial costs, lack of coach-buy in, and cybersickness, although they suggested that greater accessibility and education about its benefits could overcome these barriers.

Few studies have examined acceptability and perspectives on using VR in football specifically [11,14,26]. Thatcher et al. [11] interviewed six elite football coaches and performance analysts. The key benefits identified for use and implementation in football included opportunities to expose players to realistic game-like situations, performance analysis and player development, and to assist in recovery and injury rehabilitation. Although these perceptions point to the substantial value of VR, concerns were raised regarding the quality of the virtual environments, the limited empirical evidence base supporting its usefulness, practicality, and the financial costs of associated with use and implementation. Similarly, Dowsett et al. [26] surveyed elite football (*N = *25) and baseball (*N = *15) practitioners. Practitioners from both sports reported that the most important factors of VR training were mental, tactical, and technical on-field performance enhancements, and to aid in rehabilitation of injured athletes. The costs associated with VR training, and general negative perceptions towards the technology due to the novelty and lack of empirical research supporting the effectiveness were reported as the most significant barriers to use.

Greenhough et al. [14] added to the existing literature by surveying professional football players (*N *= 64) and practitioners working within professional football (*N *= 134) about their perceptions towards VR. Their findings revealed that both players and practitioners generally held positive perceptions towards VR and viewed it as useful for cognitive and tactical training and development. The practitioners viewed VR as useful for performance analysis, as a preparation tool for players to be exposed to unfamiliar environments, and for rehabilitation. Barriers to implementation among practitioners included financial costs, limited scientific evidence, time constraints and lack of coach and player approval. However, there was no information regarding players’ potential perceived barriers towards use and adoption of VR training.

### The Present Study

Taken together, these studies highlight the increasing interest and growing application of VR in football training, and provide valuable insight into football coaches and practitioners’ perspectives on the implementation, usefulness, and barriers of VR training [11,14,26]. However, limited research has investigated football players’ acceptability of VR, or the perspectives they have which may limit adoption of the training approach. Mascret et al. [19] demonstrated high acceptability towards VR among a sample of athletes from a range of sports that also included football. Greenhough et al. [14] included professional football players in their sample, but they did not report players’ perceived barriers towards using VR for training. Understanding footballers’ acceptability and perceptions of VR for training can contribute to a better understanding of the psychological and practical factors that may facilitate or hinder optimal adoption.

Accordingly, the present study investigated professional and semi-professional football players’ acceptability and perceptions on the use of VR in football training. Participants completed a self-report survey that was structured around the constructs of the TAM and took part in a semi-structured interview which explored the constructs of the TAM and broader perceptions of the benefits and barriers of using VR for training. Ultimately, we sought to investigate football players’ (1) attitudes, (2) perspectives on usefulness, (3) perspectives on ease of use, (4) perceptions on the benefits, and (5) perceptions on the barriers of using VR in a football training.

## Methods

### Philosophical orientation and research design

The present study adopted a qualitative cross-sectional research design to investigate football players’ acceptability and perceptions towards VR training. This study was underpinned by a relativist ontology and an interpretivist epistemology. A relativist ontological position assumes that there are multiple interpretations of reality shaped by an individual’s experiences in social contexts [27]. An interpretivist epistemology assumes that knowledge is shaped by an individual’s subjective interpretations, social interactions and lived experiences [28]. A qualitative, interpretivist approach is particularly useful when seeking to understand how individuals feel about, experience, and perceive a phenomenon [29]. It was therefore considered the most appropriate methodological orientation for the present study, as it was expected that the footballers would hold both shared and different perspectives towards the use of VR because of their different interpretations, lived experiences, and social contexts, all of which can shape their perceptions of reality [27].

### Sampling and recruitment

A purposive sample was recruited via pre-existing contacts and snowball sampling. In total, seven professional (one male and six females) and seven semi-professional (five males and two females) football players with a mean age of 25.14 years (*SD *= 3.80) participated. Eligible participants were approached and invited to participate. The majority of the athletes were recruited from football clubs in South-East Queensland (*n *= 11), with others from other regions in Australia (*n* = 2), and one from overseas (*n* = 1). The athletes were classified as Tier 3 (i.e., highly trained athletes competing at national level, *n *= 12) and Tier 4 (i.e., elite athletes competing at international level, *n *= 2), based on the participant classification framework by McKay et al. [30]. See Table 1 for further details of the participants’ demographic information.

**Table 1:** Participant Demographic Information.

Participant	Gender	Age	Playing Position	League
1	Male	26	Forward	Semi-Professional
2	Female	23	Forward	Semi-Professional
3	Male	26	Defender	Semi-Professional
4	Male	20	Midfielder	Semi-Professional
5	Female	30	Forward	Professional
6	Male	21	Defender	Semi-Professional
7	Male	25	Defender	Semi-Professional
8	Female	32	Midfielder	Professional
9	Female	21	Midfielder	Professional
10	Female	22	Midfielder	Professional
11	Female	29	Midfielder	Semi-Professional
12	Female	26	Forward	Professional
13	Female	29	Defender	Professional
14	Male	22	Midfielder	Professional

Participants were eligible to participate if they were professional or semi-professional footballers with at least one season of experience in their league. Selecting participants playing at this level was deemed as appropriate because they have sufficient experience within high-performance training and competition environments to reflect on how VR may be integrated into their regular training routines, and its potential benefits on performance. No reimbursement was offered for participation. Participant recruitment continued until no new, meaningful information relevant to the study aim was emerging. Through ongoing discussions between the members of the research team throughout all stages of data collection and analyses, it was determined that we had reached an adequate sample size to generate valuable insights related to the aims of the study. Although data saturation is a common rationale for determining sample sizes in qualitative research, Braun and Clarke [31] have criticised the concept of data saturation and argued that it does not align with reflexive thematic analysis approach because there is always potential for new insights and understanding. Of note, our sample size is similar to other qualitative studies investigating attitudes and perceptions to VR across various contexts including sport, higher education, and healthcare [15,32,33].

### Data collection and materials

The study was conducted between May 4th, 2023, and October 12th, 2023. The study protocol was approved by the Griffith University Human Research Ethics Committee (Ref No.: 2023/042). After ethical approval had been obtained, the survey and interview guide was pilot tested on two novice football players to ensure appropriateness of the interview structure and questions. The pilot participants’ responses were used to adapt the interview guide and their responses were not included in the analyses. Participants reviewed an information sheet and provided written informed consent. It was made clear that there were no right or wrong responses to the survey items or interview questions, and that the study aimed to understand their perspectives on VR training, regardless of whether they were positive or negative. All participants were informed of the possible applications of VR and its relevance in football training, and provided an opportunity to ask any questions relating to the use prior to taking part in the study.

The participants completed a survey and took part in a qualitative semi-structured one-on-one interview. The survey consisted of 15 items (divided into the subscales of: perceived usefulness, perceived ease of use, attitudes, and behavioural intentions to use) adopted from Liao et al. [34]. Some wordings of the items were amended to fit the survey to the context of using VR in football training. Each item was measured on a 5-point Likert scale (1 = strongly disagree to 5 = strongly agree), and scores were averaged for each subscale (See S1 File). Following completion of the survey, the participants completed a one-on-one semi-structured interview with the first author. The semi-structured interviews lasted up to 22 minutes (*M* = 17.04, *SD* = 3.55). Of these, 11 interviews were conducted in-person, and 3 were conducted online via a videoconference platform. The interviews were audio recorded for later analysis.

Each interview followed a semi-structured, open-ended, and conversational approach to develop rapport and allow participants to elaborate on ideas of perceived importance and for new discussions to occur. Probing questions were used to encourage participants to further elaborate on their responses. The interview questions were informed by the TAM [4]. The interviews began with introductory questions into the footballers’ broader experiences with VR within and outside football training. Following this introduction, the footballers’ attitudes and acceptability towards using and implementing VR in training were explored (e.g., ‘What do you think about implementing VR training into your existing training routine?’). The next part of the interviews examined the players’ perceived usefulness, ease of use, benefits, and barriers on using VR for football training (e.g., ‘How do you think VR could be used in football training?’, ‘Are there any particular drawbacks or challenges that you anticipate when it comes to using VR in your training? ‘) (see S2 File for full interview guide). At the end of each interview, the interviewee finished with an overall reflection of the discussion to check understanding and to ensure nothing had been missed or misinterpreted.

### Data analysis

Descriptive statistics and frequencies were calculated for the participant demographic information and the survey responses using SPSS Statistics Version 29.0 [35]. The interviews were transcribed verbatim by the first author. An independent researcher reviewed four interview records and confirmed that the content had been accurately documented. In line with our philosophical approach which suggest that researchers bring different beliefs, values, and experiences to the interpretation of participant perspectives and experiences, a reflexive thematic analysis was conducted of the transcriptions following the guidelines by Braun and Clarke [36]. The first author read and re-read each transcript to gain familiarity with the data. Next, initial codes were generated by assigning descriptive labels to each of the codes and supporting these with relevant verbatim quotations from the raw interview transcripts. The initial codes were reviewed to identify similarities, and related ones were collapsed to generate initial themes. Each theme was supported with verbatim quotations from multiple transcripts (see S3 File for the full interview transcripts). This process was repeated to ensure the codes and themes presented a nuanced interpretation of the footballers’ perspectives. Finally, the themes were reviewed and refined, and any related themes were collapsed. The themes were organised with their associated codes and relevant verbatim quotations to ensure they were supported by data.

To encourage reflexivity and enhance credibility of the findings, several steps were taken. Firstly, following each interview the first author wrote notes about their initial interpretations and reflections about the emerging patterns in the data. This process helped the researcher acknowledge personal biases and assumptions and reflect on how these influenced their interpretations of the data. Secondly, regular team meeting were held between the first and second author throughout all stages of the study including the development of the interview guide, data collection and data analysis process to encourage reflexivity. These meetings aimed to create a collaborative analytical approach and enhance the credibility of the findings by discussing the emerging codes, themes, and interpretations of the findings. Thirdly, a researcher outside of the primary research team independently coded seven randomly selected transcripts using the same analysis technique as the first author to facilitate discussions around alternative interpretations of the coding. Both the second author and researcher outside of the primary research team acted as critical friends, further supporting reflexivity by encouraging the first author to reflect on the findings from multiple perspectives [37]. The codes and themes identified by the first author and the research assistant were largely similar, and any differences in the coding interpretations and themes were discussed with the aim of enhancing the depth and richness of the analysis [37].

## Results

### Survey responses

**Fig 1:**
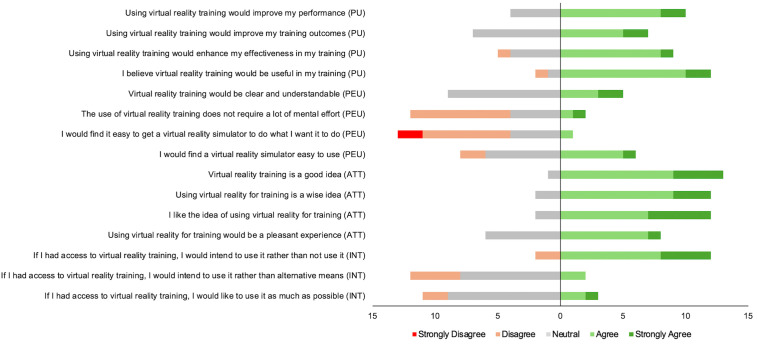
Frequencies of the footballers’ responses across the survey items rated on a 5-point Likert Scale. Responses ranged from Strongly Disagree to Strongly Agree. The central vertical line represents the point of the scale in which responses to the right indicate agreement. Note: PU = Perceived Usefulness; PEU = Perceived Ease of Use; ATT = Attitudes; INT = Intentions to Use.

Fig 1 displays the frequencies of responses across the survey items, ranging from “strongly disagree” to “strongly agree” (see S4 File for the raw survey responses). Agreement with a survey item is indicated by responses of “agree” and “strongly agree”. As can be seen, the footballers generally had a moderate to high level of agreement towards using VR for training. As a group, the footballers tended to show stronger agreement to items related to the usefulness of VR and their general attitudes about VR than for items related to its ease of use. For items that were specifically about future intentions to use VR for training, the footballers expressed high agreement that they would use it if they had access. However, there was less agreement among footballers about using VR training instead of alternative approaches, or to the idea of using it as much as possible.

### Qualitative data

The aims of the interviews were to explore football players’: (1) attitudes, (2) perspectives on usefulness, (3) perspectives on ease of use, (4) perceptions on the benefits, and (5) perceptions on the barriers of using VR in football training. In response to these aims, we identified eight higher-order themes which are presented in Tables 2–4, along with the codes from which they were generated, and examples of representative quotations. Each higher-order theme is discussed in detail below.

**Table 2:** Footballers Attitudes towards using VR in Football Training.

Higher-order theme	Codes	Number of footballers contributing data to the theme	Representative examples of footballers’ perspectives
**Favourable Predispositions Towards VR in Football Training**	Accepting of VR	14	*‘I think there’s only gains to be made from it and only positive impact to be made from it’*(P14).
	Openness to Use	14	*‘Oh yeah, definitely* [open to using VR training]. *I feel like it’s one of those 1%’s’* (P10).
	Desire for Additional Training Approaches	10	*‘It would be* [an] *awesome resource for training, you know, of course you’ve got to go out there and run and do things physically and all that - do all that. But you know, just an additional resource to reinforce things’* (P13).

**Table 3:** Footballers Perspectives on the Usefulness and Perceived Benefits of using VR in Football Training.

Higher-order theme	Codes	Number of footballers contributing data to the theme	Representative examples of footballers’ perspectives
**Cognitive Training and Development**	Decision-Making	10	*‘You know when you come um to making better decisions, sharper decisions, quicker decisions, you know, that’s what it comes down to, I suppose. Especially late in the game when you’re tired and stuff and your brain’s not working as quick. You know, you might have that, that advantage because you’ve been in this scenario before - something like that, you know. Um so yeah, I suppose like through quicker thinking is probably where it’s gonna help, I think’* (P3).
	Scanning	5	*‘Like under pressure, when you receive the ball under pressure, being able to scan and analyse the situation around you, that’s one thing I would use it for’* (P8).
	Anticipation	6	*‘Reading the game is super important, so I could definitely see that* [VR training could be beneficial here]. *Like we were saying, exposing yourself to those situations, if you could put on a headset and be seeing the field and seeing player movements and talking through that with the coaching staff - about what you’re looking at, what you should be looking at, like that’s a key part of the game’* (P7).
	Tactical Awareness	9	*‘I think definitely like tactics and positioning wise cause sometimes you just have a cone as a person, so if you have an actual person kind of thing - then you know where to go’* (P2).
	Positional Awareness	3	*‘I think it would be good for like positioning and stuff like that, because you can’t always in training get kind of game environment so, yeah, that would be good’* (P2).
**Perceptual-Motor Skill Training**	Set Piece Training	6	*‘I think um like from a deadball, like a free kick, or like, yeah, like a goal kick or a corner even, I think they’re really specific techniques that you can like do over and over and over again to get the right um... I guess the right technique and right um spot. So I think like those sort of situations would probably be the easiest to work on’* (P10).
	Technical Skill Training	9	*‘Practising free kicks... Cause like you could see where the goalkeeper is and like if you’re from this angle, you need to do this kind of shot, if you’re in this angle, need to this kind of shot. You just practise that over and over and over again I guess with virtual reality’* (P2).
	Heading Training	3	*‘If we could practice that* [heading the ball] *without actually the impact… Amazing’* (P13).
**Mental Preparation and Pressure Training**	Pressure Exposure Training	12	*‘Obviously, your bigger pressure moments, you know, where you have to step up and take them* [penalties]*. Um and, you know, no matter how many times you do it in training, it’s never the same as when it comes to the time and the actual game. So I suppose preparing yourself for that I think in virtual reality would probably be better than doing it at training. You can’t emulate that atmosphere or anything like that, you know, or that moment when you’re at training. Um, whether virtual reality could obviously do that a better, like point of that’* (P3).
	Familiarisation with Competitive Environments	12	*‘Exposing you to those, to those niche kind of experiences, like I was saying, like being within a stadium under pressure - if you can emulate that in kind of a virtual reality world um and try and get players to perform tasks, then that could be… So that when they actually experience it, if it is like a grand final, or like a, or a cup game where there’s a lot of pressure - if you can emulate that and transition and get those players to be exposed to it earlier, then they’re gonna be a lot calmer on the day’* (P7).
	Mental Skill Training	11	*‘I guess you can train another part of your mind to do something, um and just, I guess to see it and having that kind of thing in front of you as opposed to being on the field and exhausting your body. If you can train your mind, I think that could be really useful’* (P1).
	Pre-game Mental Preparation	6	*‘I think we always, everyone always does a physical warm up. You know if you could do that mental warm up all the same, whether it’s 20 minutes, half an hour and then you do 30 minutes-45 minutes on the field. That kind of all together would really, yeah, I think that would work’* (P1).
**Enhanced Training Accessibility and Flexibility**	Repeated Training Solutions	8	*‘To like, like increase repetition in that* [virtual environment] *would definitely like improve players, the, the ability of players to adapt to situations. I think for me that’s the best’* (P8).
	Individualised Training	6	*‘I think it’s just like a really good individualised sort of tool that you can use. Cause I guess a lot of training we do is very broad because like, there’s a whole, you know, team working on it, I guess’* (P10).
	Injury Rehabilitation	8	*‘It would be good for a club to have, you know, a player who’s injured, can’t be out in the field, so you put him through this virtual reality, so, you know, in his mind he’s still training’* (P3).
	Easily Accessible Training	5	*‘I think the big thing is it can be done anywhere, anytime type of thing’* (P6).

**Table 4:** Footballers Perspectives on Ease of Use and Perceived Barriers of using VR in Football Training.

Higher-order theme	Codes	Number of footballers contributing data to the theme	Representative examples of footballers’ perspectives
**Availability and Affordability**	Financial Costs	6	*‘The only barriers I could see is that I think it would be quite expensive’* (P2).
	Lack of Access	6	*‘Accessibility, that’s probably one of the biggest barriers’* (P4).
	Gender Gap for Resources	4	*‘Just that it’s expensive, I think… They wouldn’t give it to the girls, they would give it to the men’* (P2).
	Easy to Implement if Accessible	3	*‘To use for a team like ours, for example, like it would just mean that the club need to buy a set of virtual reality, or have a room accessible where you could do it in. Ummm so I don’t think that’s hard at all’* (P12).
**Technological Challenges and Constraints**	Lack of Realism	4	*‘In terms of like, we want to reproduce something real and with that* [VR environment]*, it wouldn’t be how you be in a game, you wouldn’t have that* [HMD] *on your face in the game. So then it’s like um, would you feel as comfortable as in a game?’* (P8).
	Mental fatigue	3	*‘You might use it as a preparation during the week, but before a game, might be too much. Cause you know, you can be mentally tired’* (P5).
	HMD Bulky	3	*‘Like wearing the whole thing* [the HMD]*, like it’s not really like um.. It change aspect of how you would feel in like, because you would wear something like this, in a game you wouldn’t. So potentially that could bother you’* (P8).
	User-friendly	3	*‘If I had access to it, I guess I’d probably want to know like, yeah, like how I would use it and, you know, know all those specifics. But yeah, I think it, I mean if it’s explained and pretty straightforward, I think it’d be pretty easy’* (P10).
**Time Investment**	Lack of Available Time	7	*‘The things that could stop you* [from using VR training] *is purely time’* (P3).
	Enjoyment High	3	*‘I feel like I would enjoy it too much, cause, you know, it’s so much different’* (P6).

### Football Players’ Attitudes towards using VR in Football Training

**Favourable Predispositions Towards VR in Football Training.** Only three of the interviewed footballers had prior experience with VR, yet no players had used it for football training prior to the interview. Despite this lack of experience with VR, all footballers held positive attitudes towards the use of VR for football training, showing acceptability towards the technology as a training tool and the opportunities it offers (see Table 2). Several players expressed excitement about the potential use of VR training and noted that if the technology was available, they would be open and interested in implementing it into their existing training schedules for training and performance enhancement purposes. Several players emphasised the importance of using *‘everything that you can to make yourself better’* (P1) to enhance their performance: *‘I absolutely would* [use virtual reality].. *Just because, yeah, I wanna do everything I can to be better’* (P13).

Several footballers also noted a desire for new and complementary training solutions and perceived VR as a great additional tool which could support their existing training protocol: ‘*For me I think it* [VR training] *would be a good addition to um, to support my training’* (P6). Further, the footballers viewed VR as ‘*an additional resource to reinforce things’* (P13) and described how the capabilities of VR could help them enhance their skill set and overall performance more efficiently: *‘I don’t see why virtual reality couldn’t be a tool to enhance training and performance and things in addition to what you’re normally doing’* (P7).

### Football Players’ Perspectives on the Usefulness of using VR in Football Training

**Cognitive Training and Development.** As shown in Table 3, all the footballers agreed that VR training has the potential to enhance key cognitive abilities which are essential to achieve high-level performance in football, including decision-making, scanning and anticipation, and for tactical training and in-game positioning. Specifically, players noted that practising in representative training environments that replicate realistic game-like scenarios could help enhance their scanning and decision-making under pressure: ‘*I think for me a lot of it would be my decision making. Even when to shoot, when to pass’* (P11). Several footballers also suggested that VR training could improve their ability to read and anticipate the movements of the ball and opponents, enabling them to make more proactive decisions on field. Additionally, players viewed VR as useful for enhancing tactical awareness and in-game positioning through simulations of complex tactical game situations: *’If you can emulate kind of positional awareness or like if there’s some sort of uh tactical thing that the coach wants the team to do, and if you can emulate that in a virtual reality scenario’* (P7).

**Perceptual-Motor Skill Training.** Many footballers viewed VR as a useful tool for practising various football-relevant technical skills including shooting, dribbling, and passing under game-like conditions to enhance their technique, shooting accuracy, and overall performance: *‘You could probably zone in on, say where you would need to hit the ball, or how you would need to hit the ball. You could probably tailor it to slow it down and certain - when to meet the ball and things like that’* (P1). Furthermore, footballers described that VR could be a valuable tool for refining the execution of set pieces and goal kicks: *‘Like a free kick, or like, yeah, like a goal kick or a corner even, I think they’re really specific techniques that you can like do over and over and over again* [in a virtual simulator] *to get the right technique’* (P10).

Several footballers further discussed the potential advantages of using VR for heading training. Some players raised concerns regarding the long-term cognitive impact real-world heading drills may have, and described how VR training could provide a safe alternative to heading training to mitigate these risks while still improving their heading technique: *’Practising attacking corners, practising defensive corners* [in VR] *would be awesome. Because I actually really find that like my brain capacity from heading the ball could potentially be really affecting me later in life and when we just practise one or two, just to get the movement right and it’s like rock hard, you know, it’s, it’s really affecting cognitively my brain’* (P13).

**Mental Preparation and Pressure Training**. All footballers viewed VR as a valuable training tool to aid with mental game preparation and to get in the right mindset before a match: *’I actually think even before you’re about to play, or before you’re about to train that would be a really good way to try and get into that mental state of mind’* (P1). Many players also perceived VR as a great tool for creating pressure during training to bridge the gap between training and high-pressure game scenarios: *‘You can’t recreate those kind of situations* [pressure] *at training. So being able to have this add up to your training, that will definitely be like another benefit’* (P8). Further, the footballers described how repeated exposure to representative high-pressure situations, coupled with the simultaneous practice of specific skills, could facilitate skill automaticity, enhance their ability to adapt and block out external distractors, promote more efficient decision-making, and enhance their ability to better regulate and manage emotional responses, ultimately enhancing their performance on field: ‘*I guess exposing yourself to that higher pressure situation in training could correlate to better performance on the actual day’* (P7). Other participants viewed VR as a *‘good tool for extending our mental capacity’* (P11) and to maintain their mental capabilities during periods of reduced physical training, such as injury rehabilitation: *‘When you can’t always be at the pitch, you know, that’s where, that’s something where it would probably be good, if someone is injured, for example’* (P3).

Similarly, many footballers viewed VR as a great tool for younger and less experienced players to build mental resilience and confidence in their abilities to perform and cope under pressure through exposure to representative high-pressure environments: *‘If you can get the young players that are not experienced to be readier mentally using virtual reality, that will be interesting. Just because they’re being exposed to those scenarios virtually, even though they haven’t in the real life, but when you replicate that in real life, will they, will they feel more prepared for it?’* (P5).

**Enhanced Training Accessibility and Flexibility.** Many footballers indicated that one of the major benefits of VR training is that it makes training more accessible because it eliminates the need to physically attend the training ground, and provides opportunities for training when on-field training is not possible due to factors such as poor weather: ‘*I think it* [VR training] *probably could become a very, more of a positive, um because, you know, you don’t always have access to the fields and whatever else. Same thing for wet weather’* (P3). Several footballers also emphasised the potential of VR to assist with recovery and injury rehabilitation and to reduce the risk of overtraining injuries. They noted that VR training could allow players to stay mentally engaged in football and to maintain and/or improve their mental sharpness and cognitive skills through training in realistic football environments, despite being unable to physically train. For example, P10 noted: *’It’s a way of you still improving, even when you’re injured’*, followed by *‘I guess it could improve, or like keep your, I guess, mental sharpness’.*

Other participants highlighted the opportunities for VR training protocols to be tailored to the needs of each individual player, allowing players to focus on specific skills or aspects of the game they need to improve on: *‘I guess, if you were repeating the same sort of situations that, you know, is more um fitted to what you might face in the game, um yeah, you just get better at those situations and decision-making and pulling under pressure’* (P10).

### Football Players’ Perspectives on Ease of Use and Barriers of using VR in Football Training

**Availability and Affordability.** The most commonly identified challenge to VR training was the lack of availability and access to VR equipment (see Table 4), which limited the players opportunities to use the training tool: *‘I just think the accessibility of it would be the biggest probably barrier’* (P7). Further, many players noted that the financial costs associated with the technology could further limit the access and usage further: *‘The financial means would probably be the biggest issue for a lot of teams’* (P3). Several female footballers also noted a lack of financial resources in Women’s football programmes which could further limit the access to VR training: ‘*A lot of clubs don’t have a lot of money, or especially for their women’s programs, maybe they give it to the men, but that’s just the daily battles that we face. So yeah, maybe financially’* (P13). Some players indicated that if they had access to VR training, they would be willing to use it, and viewed it as a training tool which could be easily implemented into their existing training schedules: *‘Yeah* [it could be easily incorporated]*, because it’d just be something like, let’s say, if there was a headset at the club and I wanted to come do some VR training for half an hour before the session, or after the session - that’s easy’* (P7).

**Technological Challenges and Constraints.** Some players reported a number of technological challenges and constraints of VR training, including the realism of the content within a VR environment, which they worried could disrupt real-world performance: *’Maybe it’s not fully realistic, so maybe something, if you’re training like that and then you get to the game and it’s a bit different… It can affect your performance in, I guess, the real world by using the virtual reality training’* (P4). Some participants further doubted VR’s capabilities to accurately replicate the physical sensations and pressure of a real-world match environment: *‘I think pressure is difficult* [to recreate] *because you need to have something to lose in a sense. In that moment you need to be able to give something up’* (P1).

A number of players also raised concerns regarding the head-mounted display (HMD) being noticeable or uncomfortable which could increase the difficulty of use, reduce enjoyment, or cause players to become distracted or disengaged during the training: *‘I would say some people probably wouldn’t feel very comfortable with it* [the HMD]*‘* (P8). Some footballers also identified possible side effects of VR training, such as mental fatigue and motion sickness, which could limit the use due to its potential negative impacts on real-world performance: *’The mental fatigue of it…. So like, if you do too much and then you don’t have enough time to process, and then you’re actually mentally tired for the game. So I think that that will be a barrier’* (P5). Despite these factors which could hinder optimal uptake, the footballers perceived VR as a technology which would be easy to use, given they were given instructions of how to use it: *‘It would be pretty easy, without ever using one myself, but when I’m seeing other people use it or videos, it seems pretty straightforward. But yeah, as long as it was explained to us and you know, we got to test it out first I think there’s no reason why anybody couldn’t pick it up’* (P14). Further, a player highlighted that ‘*making it easy for the players is the biggest thing* [because] *as soon as you make it difficult, they don’t wanna do it’* (P7).

**Time Investment.** Many footballers emphasised the importance of physical on-field training for team cohesion, skill development, and overall performance: *‘I feel training hours, you know, you need to be there with your team and actually experience it all at the same time’* (P3). Furthermore, some players noted that VR training might detract from real-world training due to the novelty and heightened enjoyment of the training approach: *‘For some people there could be a bit too much focus on it and I guess that could take away from other parts of training’* (P10). Other factors which could limit uptake of VR included *‘even just finding the time’* (P11) because of work and personal commitments outside of football: *‘You can always do extras but when, when you have all the like, all the stuff, it’s a bit harder. Most players study, work, so can they actually fit that in their schedule is the question’* (P8).

## Discussion

The present study aimed to gain insight into football players’ acceptability and initial perceptions towards the use of VR in football training. Overall, the players were accepting towards the use of VR and viewed it as useful for enhancing football training and performance. While their survey responses indicated more neutral perceptions of ease of use and behavioural intentions to use, many players expressed a willingness to use VR training, if it was available as an option. Collectively, the findings suggest that footballers hold favourable attitudes towards the use of VR for training and perceive it as useful for training and performance enhancements, while acknowledging a number of practical and psychological factors which could limit adoption. Our findings extend upon existing research and indicate that footballers are accepting towards the use of VR training and perceive it as useful for training and performance enhancement, indicating that VR training may be easily implemented within football contexts.

The footballers identified several areas where they perceive VR-based football training could be useful to facilitate greater on-field performance. Specifically, the players perceived VR as a valuable addition to real-world training for cognitive skill training to enhance decision-making, scanning capabilities, anticipation skills, tactical understanding, and for perceptual-motor skill training. These findings are in line with the existing use of VR among professional football clubs [3], and resonate with existing experimental research which have shown that VR training have effectively improved footballers’ real-world heading abilities [7], decision-making and visual search behaviours [38].

Several footballers also highlighted that VR training could aid in pre-game mental preparation by enabling them to visualise and gain exposure challenging game situations, help them get in the right mindset before a game, and enhance their ability to manage their emotional responses under pressure. Experimental studies have documented that training with pressure has reduced performance anxiety [39] and improved performance under pressure in athletes [40]. More recent research has documented that VR sport-specific environments have induced somatic and cognitive anxiety responses in athletes [9,10,41]. However, it remains unknown whether exposing athletes, including semi- and professional footballers, to realistic, high-pressure virtual environments can enhance their performance in real-world competition, although this has been suggested as a possibility in past research [6,42].

The footballers also reported that VR training could help mitigate injury risks and support rehabilitation by enabling safer practice of crucial skills (e.g., heading) without the physical strain and potential injury risks from repeated head impacts or potential overtraining injuries. Additionally, the players noted that VR could help injured players maintain a level of tactical understanding, cognitive involvement, and mental sharpness of the game. Professional football clubs are currently using VR for skill training and rehabilitation allowing players to safely engage in football-specific movements and perceptual-cognitive skill training in controlled VR environments [43]. The use of VR for rehabilitation aligns with football practitioners’ perceptions that VR could be useful for supporting injured or rehabilitating players [11,14,26].

Although all footballers generally had positive perceptions on VR training, some factors which could hinder adoption of the training approach were identified. The most common challenges regarded the limited availability, and the financial costs of the technology. These perceptions align with those of football practitioners [14,26], who identified the financial costs of VR as the biggest barrier towards use. The players expressed uncertainly about the costs of implementing and maintaining VR training, and noted that their clubs may not have the financial resources to invest in innovative training approaches, such as VR, particularly within Women’s football leagues.

Moreover, some footballers noted technological constraints of VR including lack of realism and perceptual differences between the real-world and the virtual environment which could limit adoption. These perceptions of VR may stem from the players lack of experience with VR simulations, as they may lack understanding of the quality and capabilities of the technology. Existing evidence has demonstrated that a football-specific VR simulator successfully differentiated between footballers who had reached various performance levels, suggesting a level of congruence between the VR simulator and the real-world [44]. Similar findings have been observed in a VR goalkeeping task, with elite goalkeepers waiting longer before initiating movement to catch a ball compared to novices [45], and in a VR golf-putting simulator where elite-level golfers outperformed novices when comparing putting accuracy [46].

Further, some players raised concerns that training using VR could induce mental fatigue which could negatively impact their real-world training and performance. To our knowledge, no empirical work has investigated athletes’ experiences with mental fatigue from VR training, and no guidelines of how to effectively carry out VR training with minimal risks of mental fatigue have been proposed. As such, future studies should examine how VR training impacts mental fatigue, and investigate the optimal training duration, intensity and task difficulty in VR training before potential fatigue onset. Guided by established principles from traditional football training, a gradual, phased approach to introducing VR training might help athletes become familiar with, and adapt to the demands of VR training. For example, coaches could begin with shorter VR training sessions consisting of simple drills that impose low cognitive load on players, and progressively increase the training duration, task difficulty and complexity, allowing players to adapt to the cognitive demands of the training. This approach aligns with the principles of progressive overload and load management, which are commonly applied in real-life football training to optimise performance outcomes whilst mitigating the risks of injury and fatigue in football players [47,48]. Building on these principles could guide effective VR training protocols and highlights the need for future research to investigate this.

The findings of the present study are in line with the TAM, particularly highlighting that perceived usefulness is a significant factor for an individual’s acceptance and intentions to use and adopt a new technology [4]. Overall, the footballers generally expressed positive attitudes towards the use of VR for football training and perceived VR as a useful tool for enhancing their performance. The participants also expressed a willingness to use any training tool with potential to improve their performance, suggesting that they were generally open towards trying new training solutions, including VR. Additionally, the players outlined specific features of VR training, such as the ability to train in representative, game-like environments, and the flexibility to train at any time, regardless of time of the day or weather conditions, as benefits of the training approach. These findings suggest that if VR training was available for the players to use, they would likely adopt the training approach. However, future research should investigate whether the players’ willingness to adopt VR training is driven by a general openness to try new training methods with potential to enhance their performance, or by the specific benefits that VR training offers.

Overall, the footballers’ perceptions of how easy VR would be to use were neutral across their survey responses. Perceived ease of use has been documented to be less influential in explaining technology acceptance compared to perceived usefulness, because it relates to the technical aspects of using a technology, rather than the practical performance benefits [4]. Further, some studies have found that perceived ease of was not a significant predictor of user acceptance or intentions to use new technologies [49–51]. It is possible that footballers place a greater emphasis on the potential performance benefits of VR training than the technical aspects of how to use the technology, as elite athletes are always seeking additional ways to gain a competitive advantage and maximise their performance potential. Another possible explanation for the neutral perceptions of ease of use could be due to the players’ lack of experience with VR, as they may lack knowledge of how to use and navigate VR training systems. As such, football players’ perceived usefulness of VR may be more important in explaining their attitudes and intentions to use VR than perceived ease of use, in line with the TAM [4].

In the interviews, the footballers noted that VR training could be easily implemented and used if they were given clear guidelines and adequate support of how to use the technology. Providing players with an opportunity to experience VR training, along with clear instructions and guidelines of how to best use the technology, may help reduce any negative perceptions of the technology and promote greater acceptance of VR as an effective training tool, ultimately increasing usage [4]. To further encourage use and adoption among players, coaches and performance support staff should inform players of the performance-related benefits VR training offers, as players are more likely to engage with training tools that are endorsed by influential figures including their coaching team [14]. Furthermore, presenting examples of elite-level footballers utilising VR training may promote positive attitudes and further encourage adoption among professional and semi-professional players, who are often influenced by the practices of higher-level players (e.g., Premier League players).

### Study limitations and future research directions

Although the findings provide valuable insights into elite football players’ acceptability and perceptions regarding VR training, there are some limitations which should be noted. Firstly, the sample consisted of primarily football players competing in Australia, which could limit the transferability of the findings to players from other geographical areas (e.g., Europe, Middle East). It is possible that football players from different leagues around the world (e.g., Premier League, Saudi Pro League) will hold different levels of acceptability and perceptions of VR training due to different levels of exposure to performance-tracking technologies (e.g., GPS trackers, video analysis technology). Studies using larger and more diverse samples of football players would strengthen transferability of the findings and provide a more comprehensive understanding of footballers’ acceptability and perceptions of VR. Secondly, the sample consisted of semi-professional and professional players. There may be differences in acceptability and perceptions of VR training based on individual and contextual factors such as performance demands and expectations, time available, access to, and experiences with novel training methodologies. As such, future studies could recruit larger samples of players across varying competitive levels to examine the potential differences in acceptability and perceptions of VR training.

Finally, the present study focused on football players’ acceptability and perceptions of VR training. While this is an important step in understanding key factors which influence initial uptake and adoption of a technology [52], there could be value in examining football players’ acceptance and perspectives of VR training after regular use. Comparing acceptance and perceptions of VR training before and after use could provide a greater insight into footballers’ perceptions change following experience with the technology, and highlight any factors which may facilitate or hinder continued use and adoption. Comparing user acceptance before and after use is a common approach in technology adoption literature employing the TAM [52]. When evaluating footballers acceptance and perceptions to VR after use, it would be good to include confirmation of the players’ expectations and their satisfaction with VR, as these factors are crucial to understanding continuance intentions towards a technology [34]. Future studies could also explore the acceptability and perceptions of VR training among athletes from other sports to examine whether their perspectives and expectations around the usefulness, ease of use and intentions to use VR are comparable to footballers.

## Conclusion

The present findings revealed that the footballers held favourable perceptions towards the use of VR training, viewed the technology as a valuable adjunct to their existing training, and expressed a modest desire to adopt the training approach. In addition, the players identified a number of factors which could limit the uptake of the training approach, including the financial costs, limited accessibility, and potential perceptual differences between real-world and VR training. These findings offer valuable implications for football coaches seeking to implement VR into their existing training protocols and suggest that the players would accept and adopt VR training if it was available. To further encourage adoption, coaches and practitioners could present evidence-based examples of how VR have been used among professional teams to highlight the benefits of VR training and facilitate discussions around the advantages of VR.

## Supporting information

S1 File Survey. (DOCX)

S2 File Interview Guide. (DOCX)

S3 File Interview Transcripts. (DOCX)

S4 File Survey Responses. (XLSX)
