# Supplementary material for: A qualitative examination of football players’ acceptability and perceptions on the use of virtual reality in football training
Source: PLoS One. 2025 Oct 9;20(10):e0334167. doi: 10.1371/journal.pone.0334167 (PMC12510607; doi:10.1371/journal.pone.0334167)
Supplement: S1 File — (DOCX) [file pone.0334167.s001.docx]

*Please read each statement carefully and rate your level of agreement using the scale below on each of the statements in relation to your thoughts regarding the use of virtual reality in football training.*

|  | Strongly disagree | Disagree | Neutral | Agree | Strongly agree |
| --- | --- | --- | --- | --- | --- |
| Using virtual reality training would improve my performance | O | O | O | O | O |
| Using virtual reality training would improve my training outcomes | O | O | O | O | O |
| Using virtual reality training would enhance my effectiveness in my training | O | O | O | O | O |
| I believe virtual reality training would be useful in my training | O | O | O | O | O |
| Virtual reality training would be clear and understandable | O | O | O | O | O |
| The use of virtual reality training does not require a lot of mental effort | O | O | O | O | O |
| I would find it easy to get a virtual reality simulator to do what I want it to do | O | O | O | O | O |
| I would find a virtual reality simulator easy to use | O | O | O | O | O |
| Virtual reality training is a good idea | O | O | O | O | O |
| Using virtual reality for training is a wise idea | O | O | O | O | O |
| I like the idea of using virtual reality for training | O | O | O | O | O |
| Using virtual reality for training would be a pleasant experience | O | O | O | O | O |
| If I had access to virtual reality training, I would intend to use it rather than not use it | O | O | O | O | O |
| If I had access to virtual reality training, I would intend to use it rather than alternative means | O | O | O | O | O |
| If I had access to virtual reality training, I would like to use it as much as possible | O | O | O | O | O |
