# Supplementary material for: A qualitative examination of football players’ acceptability and perceptions on the use of virtual reality in football training
Source: PLoS One. 2025 Oct 9;20(10):e0334167. doi: 10.1371/journal.pone.0334167 (PMC12510607; doi:10.1371/journal.pone.0334167)
Supplement: S2 File — (DOCX) [file pone.0334167.s002.docx]

**Interview Guide**

1. **What do you know about virtual reality?**
2. **Have you used virtual reality before?**

- Can you tell me about your experience with virtual reality?
- Have you used virtual reality for playing or training with football?

1. **How do you think virtual reality could be used in football training?**

- In what areas of your training do you think virtual reality could be the most beneficial?
- How about your performance during a game- in what areas do you think virtual reality training could help you improve your performance the most?
- Can you comment on any of the things that influence your likelihood of using virtual reality in your training?

1. **What do you think about implementing virtual reality training into your regular training routine?**

- Would you be willing to use it regularly? Why, or why not?
- Can you tell me about some of the things that would influence your willingness to use virtual reality training? What would need to be done to make you use it more?

1. **What specific features or aspects of virtual reality training do you think would be required for it to be useful and effective in your training?**

- Would any of these factors influence your decision to use virtual reality training, and why?

1. **Can you tell me of some of the potential benefits of incorporating virtual reality into your training routine?**

- How do you think these benefits could help you become a better football player?

1. **Are there any drawbacks or challenges that you anticipate when it comes to using virtual reality in your training? What are they?**

- What do you think could be done to overcome these challenges?

1. **Has the use of virtual reality training been discussed among your teammates, and what are the general attitudes and opinions towards the use of virtual reality in training?**

- How about with your coaches? Have they commented on the use of virtual reality in training, and what are their general attitudes towards the use of it?
- How have these attitudes influenced your ways of thinking of, or considering the use of virtual reality?

1. **Do you have any additional thoughts or comments regarding implementation and use of virtual reality-based training that have not already been discussed?**
