# Supplementary material for: A qualitative examination of football players’ acceptability and perceptions on the use of virtual reality in football training
Source: PLoS One. 2025 Oct 9;20(10):e0334167. doi: 10.1371/journal.pone.0334167 (PMC12510607; doi:10.1371/journal.pone.0334167)
Supplement: S3 File — (DOCX) [file pone.0334167.s003.docx]

**De-Identified Interview Transcripts**

**Participant 1**

Interviewer: Can you tell me about what do you know about virtual reality? Have you used it before?

Participant: I have used it once before, um it wasn't, I guess, very good at that time.

Interviewer: Yeah

Participant: Um but I think obviously it has come a very long way now.

Interviewer: Yeah

Participant: Don't know too much about it other than, other than people getting really, I guess, immersed in it and like, they think, you know - It feels very real, and then you see them doing things with their body and they flail around because they think it's very real. So yeah, I know that much of it, but I haven't used it recently so.

Interviewer: Was that for football training or outside football training?

Participant: Just like games. Like, it wasn't for football training. It was just for outside for personal use.

Interviewer: Yeah. Cool. So what do you think about using virtual reality in your training? Um would you be open to using it?

Participant: Yeah, I think, I think it would be pretty good. I think from a mental side of things. You know, there's, there’s certain things that you can do on, on the park that reaches a limit. Whereas, you know, just say you're injured for example, or, or overtraining is a real issue in sports as well. I guess you can train another part of your mind to do something, um and just, I guess to see it and having that kind of thing in front of you as opposed to being on the field and exhausting your body. If you can train your mind, I think that could be really useful. Yeah.

Interviewer: Yeah. Cool. Um do you think there are any other benefits? Um how would it um help you um perform, like during a match or just your performance in general on the field?

Participant: I guess it's hard to say because I think when you're on the field, there's certain external factors that you have to deal with. Like um.. Not just things that you can see, but things that you can feel. Things that you can kind of… I think there's a feeling to it as well. I can't, I can’t really describe it, um but I think having something is better than having nothing.

Interviewer: Yeah

Participant: Um something that you can train, something that you can use, something that helps you. I'm a big believer in everything that you can have you use to make yourself better. So I think it would certainly make you better. I think there's a space for it, I think, I think there is, yeah.

Interviewer: So you said um it could help um the mental side of the it.

Participant: Yeah.

Interviewer: Do you think um any other ways? Like could you improve your um motor skills, or like any cognitively?

Participant: Probably, yeah. I guess like the way that react you react to things, how quickly you see things. Umm so I think that way. Again, physically I'm not sure how much you can do really physically with your body um cause you got something on your head

Interviewer: Yeah

Participant: But I think when you see things and you're able to interpret things a little bit quickly, that mental sharpness which is really important in football when you can't run as much anymore and you need to always be thinking

Interviewer: Yeah

Participant: I think that's something that you can really train that probably footballers don't train enough of. Yeah.

Interviewer: Yeah. So if you had um access to it, would you be open to using it? Um as a part of your training?

Participant: Yeah, I actually think even before you're about to play, or before you're about to train that would be a really good way to try and get into that mental state of mind because sometimes I find myself personally, and in my team, a lot of players fail to, I guess, switch on for the game at an appropriate time. That's why you do a warm-up and you try and get into that state of mind.

Interviewer: Yeah

Participant: I think if you did something like that, or implemented something like that before a game, or before, before training, I think that could make a difference to - maybe not how you finish a game, but how you start a game or things like that. I think it could make a bit of a difference.

Interviewer: Cool. So are there any things like that would make you more or less likely to use virtual reality? Like any research or like benefits or barriers you perceive?

Participant: I think if there were benefits to it that would only make me more inclined to use it.

Interviewer: Yeah

Participant: I think the things that would steer me away is, I guess, the access to it- if I didn't have access, I wouldn't be able to do it. Umm if it was too difficult to do as well

Interviewer: Yeah

Participant: I think you need to make it realistic as well. So there's pros and cons, I guess, to everything, um but I would, yeah, if, I, if, if, if it was for my personal use, I think I would use it.

Interviewer: Yeah

Participant: And I think it would be of benefit to me. So it just needs to be available and I guess relevant and then… Yeah.

Interviewer: Relevant, yeah… So has the use of um virtual reality, um has it been discussed among the team members or the people you play with?

Participant: Not really, no. This is actually the first time I've kind of heard about people using it

Interviewer: Yeah right

Participant: Um for a, for a sporting point of view, so not really, no. This is the first.

Interviewer: How do you think your team members would um, would they – how would they think about it um and would they be open to using it?

Participant: Like I said, I think they would really embrace it because it can be used as a factor that.. Yeah, a different form of training.

Interviewer: Yeah

Participant: I know with footballers when we when we train a lot, say sometimes we'll train twice a day and then, you know, that, that's a lot, so you do a morning and an afternoon session and then if you have a day off, the day afterwards, instead of maybe not doing anything or if you do a bit of recovery, you do a bit of this, you know VR or whatever needs to be done and then that way you're working your mind, you're still seeing the things that need to be done. I.e., if you're taking a free kick or a penalty or, or something like that - you're able to be there in the moment, but you're not physically doing it. So you're preparing your mind, but you're not, you’re resting your body. I guess it's kind of two birds with one stone in a sense.

Interviewer: Yeah. Yeah. So sounds like you think it would be mostly useful for the mental side and sort of be immersed into these environments?

Participant: I think that's a lot of what football is. I can't speak for many other sports, but I think - the physical is to an extent, but then a lot of it is the mental. So you can only do so much training and then what your mind does after that is…

Interviewer: Yeah

Participant: Yeah. Takes over. So I think, yeah, I definitely think on the mental point that would keep you sharp and that's what it would benefit you most with, I think. Yeah.

Interviewer: Yeah. Cool. What about.. So I guess, when you saying you haven’t really heard much about it – hasn’t been discussed among your um coaching team either?

Participant: No. We do, we do quite a lot of things with, on the, you know, nutrition, health, all this different type of thing. But we don't really talk about, I guess, virtual reality or any things like that. So yeah, yeah, it's pretty new to me.

Interviewer: Yeah. Do you think they would be open to implementing it into the training?

Participant: I think so. Yeah, absolutely I think so.

Interviewer: Cool. Do you think there are any like barriers towards the use of virtual reality, um or like challenges you may perceive if you were to incorporate it into your training routines?

Participant: Umm me personally, or within like a team environment?

Interviewer: Umm.. Both, like you as an individual, but also within the team.

Participant: Yeah, well, I guess the thing is you've got 11 different players that do different things on a field. So I guess if you had a program set up for each player, I think that could be good. So again, making it relevant for each individual because what I do as an attacker might be different to what a defender might experience in a game

Interviewer: Yeah

Participant: So I think maybe those barriers is, you know, how relevant is it to each person. Um I think personally, and again it's hard because I'm not really sure, I, I haven't done too much of it and I don't know too much about it.. Uumm other than, yeah, what I've already said. Ummm any other barriers really, I guess, yeah, just the access to it and the relevance and things like that. So yeah, probably those too.

Interviewer: What do you think could um be done to overcome these barriers? You said like individualised training which is one of the benefits of virtual reality because you can create a lot in artificial intelligence and virtual reality – so it can be individualised. But um your other barriers – how do you think these could be overcome?

Participant: Yeah. Yeah. I think when we do, when we play a game, often the next week we'll do a video session of what we did right and what we did wrong. I think if you could simulate things back from the game, and if you could put yourself back in that scenario and you could see things I think that would really help. I think.. Yeah, making that individualised training that, that would really get you over the barrier. So putting everything, making, I guess setting yourself up for success in that way, I can't, it's really hard to describe, but yeah, just tailoring those things. I guess giving people access, letting as many players and coaches and things know about it so that they look to interpret it into their training.

Interviewer: Yeah

Participant: I think it's just about having people know about it.

Interviewer: Yeah

Participant: So acknowledging that it's there and then just using it and then tailoring it, you know, per person. So yeah.

Interviewer: So a lot of information and education about like the benefits and stuff?

Participant: Information... Absolutely. Yeah, I think at the moment, like I think it's a really good idea now. So. you know, I don't know too much about it. But, you know, if it's in a year or two or three down the track and it gets really good, I think there's a lot of - I think there’s room for it, certainly I do, yeah.

Interviewer: That's cool. Perfect. Umm soo can you tell me more about um any of the factors that would influence your decision to um use it? You said the individualised training, you see that as a benefit, umm and also to sort of immerse yourself into the space.

Participant: Yes.

Interviewer: Are there any other things that could be, like that you could be open to using it for?

Participant: It's a tough one because I think.. Umm, I guess, yeah I guess it depends. There, there, there are a lot of factors. I guess the more that you can recreate from the game

Interviewer: Yeah

Participant: And the more that you can kind of put yourself in that - different scenarios, doing different things umm that would kind of just be, that would be the best thing that you could do. Obviously, there's some things that you can't do. You can't connect with a ball, or you can, but in, in a certain way and I think there's a feeling to it as well. So I think there might be, maybe perhaps a degree to where it, where it, not where it kind of levels off, but I guess there's some things that you can do on the field and there's some things that you can't. So I don't really know where that line crop finishes, so yeah.

Interviewer: Yeah, and virtual reality would never, you know, replace the physical training on field.

Participant: Yeah

Interview: It's just like a, like an addition.

Participant: And added in. Yeah, exactly. And so yeah, I don't know where that kind of line, kind of starts and finishes.

Interviewer: Yeah. Yeah, yeah, yeah. And you can incorporate the physical sensation of kicking the ball and stuff

Participant: Yeah

Interviewer: But there is still the physical aspect, which is a very crucial part of sports training.

Participant: Sure, sure. Yeah, exactly.

Interviewer: Umm can you.. Are there any other factors that would influence your willingness, or your openness to using it?

Participant: Umm… Not really, like I'm, I’m pretty open to, yeah, like I said before, just having all the, like all the equipment to me.. Sorry having all the equipment um to use to make myself better. So I think being able to be given the equipment. Umm I don't know how, I don’t know how affordable it is as well.

Interviewer: Yeah

Participant: So, you know, in one team does each player have one, does someone have to share?

Interviewer: Yeah

Participant: So then that, you know that then - when you're sharing with 11 guys plus, you know, however many people sit on the bench, it's a lot..

Interviewer: Yeah

Participant: ..to kind of go through if you're doing it before a game, you have to get there six hours before the game to just use it, so, yeah, I guess the amount of people that are using it. Things like that. Yeah.

Interviewer: Yeah. Cool. So it sounds like um the benefits um you perceive are mainly the immersion, the individualised approach to training?

Participant: Yes. Yes.

Interviewer: And also, you know, there are opportunities to train cognitive skills such as scanning

Participant: Yeah

Interviewer: And perhaps the physical like drills

Participant: Yeah

Interviewer: Um perhaps heading drills…

Participant: Sure

Interviewer: Um I don’t know if you have any thoughts about how it could be used for like training, like heading, and um drills, shooting the ball and stuff like that?

Participant: I think, yeah, I think you could probably zone in on, say where you would need to hit the ball, or how you would need to hit the ball. You could probably tailor it to slow it down and certain - when to meet the ball and things like that. You can really, I guess, get into the nuance of how everything works if you're trying to really break it down.

Interviewer: Yeah

Participant: Umm from a, from a speed point of view maybe, from what you can see um point of view umm, yeah, I think those, those kind of things. Again, I, I haven't used it so I don't know exactly cause I can't imagine heading a ball with this big thing around my face. But yeah, I think, yeah, I think there's a lot of things that can be done with it, so.

Interviewer: Yeah

Participant: I certainly think so.

Interviewer: Yeah. Cool. So it sounds like you are very open towards um using it

Participant: Yeah

Interviewer: And like the barriers are mainly like the affordability

Participant: Access, yeah

Interviewer: Which it is becoming more affordable and accessible, but of course there is still a cost associated with it.

Participant: Sure. Yeah. Absolutely.

Interviewer: Cool. Do you have any other thoughts about the use of virtual reality you'd like to elaborate on?

Participant: Yeah, I guess.. I don't know how you guys um plan to like, recreate, I think you mentioned it earlier, like the pressure of things. I think that's something that's really difficult to recreate because that's, I guess, a personal kind of value to each person, how much stock they take in each situation. So, for example, when you take a penalty, you know, there's a lot of pressure on that individual person, at that individual moment and things like that. So I think there's more, that's more difficult to recreate than just something visually or just kicking a ball, if that makes sense.

Interviewer: Yeah, yeah, yeah.

Participant: So I guess that side of it is kind of - That would be really good if you could, if that's something that you could find and like tap into that, that'd be, yeah,.

Interviewer: Yeah. So do you have any thoughts about how that could be done? Any features or things you could incorporate?

Participant: I think pressure is difficult because you need to have something to lose in a sense. In that moment you need to be able to give something up, so unless the headset blows up in your face if you miss the shot. Other than that, it's, it’s really quite difficult because when you when you take a shot, the least the thing that you're thinking is about shooting.

Interviewer: Yeah

Participant You're thinking about all the other external things around you. Ummm You're thinking, you know, if I score, there's going to be great. Everyone's going to talk about me. Or if you miss, you're going to think no one was going to talk about me, I'm never going to play again, ummm I don't know how to recreate that, I don't know. You can't explain pressure other than, like a feeling in your stomach. You know, you can say what you, what you are feeling at the moment, but it doesn't exactly describe what it is so.. Yeah, it's kind of like, yeah, trying to grab air or something like that, yeah.

Interviewer: Right.. Okay. So we will getting a lot more into talking about pressure in a moment. Just wanna… I think we have covered everything. I just wanna to make a summary to make sure that I have gotten all the right information.

Participant: Yeah

Interviewer: So you generally, as we said, are positive towards the use of virtual reality and think it could be used for like immersion, cognitive training um and scanning

Participant: Yup. Yeah.

Interviewer: Open towards using it um for motor training

Participant: Yeah

Interviewer: But a little bit hesitant

Participant: Yeah

Interviewer: Umm and also um for… Umm immersion, that’s, I said that…

Participant: Yeah

Interviewer: Um and then the benefit you see are, you know, individualised training and training before going into a game, or even into training.

Participant: Yeah. Yeah, mental preparation, I guess, yeah.

Interviewer: Mental preparation

Participant: Yeah

Interviewer: So how, can you elaborate a little bit about how you think it could be used for mental preparation?

Participant: Um well, I guess the difference um when you get say out of the car and then you get into the change rooms, it's all a very slow process to when you get changed, to when you warm up and there's things that you do when you stretch and, and during that whole time you kind of are thinking about what's going to happen, or what could potentially happen. Whereas if you could see it in front of you, and you're somewhat doing it in that moment - that prepares you more, I would say, in a sense. It wouldn't prepare you fully, I don't think other than just doing it, even when you're doing it you’re not fully prepared, but I think, yeah, being able to see it in what, that cognitively, I guess in a sense, or doing it, um I think that would prepare you much more

Interviewer: Yeah

Participant: Because it's almost like you're there, but you're not having to physically strain yourself and, and things like that. So that for me personally is really good. You know, it's like, it’s a mental warm up and then you go and do the physical warm up and then you're prepared

Interviewer: Yeah

Participant: To go out and perform and do, do the best you can.

Interviewer: Yeah. So sounds like that's one of the biggest benefits you see at the moment of using virtual reality?

Participant: Yeah, I think, yeah, I think we always, everyone always does a physical warm up. You know if you could do that mental warm up all the same, whether it's 20 minutes, half an hour and then you do 30 minutes-45 minutes on the field. That kind of all together would really, yeah, I think that would work.

Interviewer: Yeah. Cool. And it sounds like you see more benefits than barriers? Like the biggest barriers you see are the affordability, accessibility?

Participant: Pretty much, yeah.

Interviewer: Um, and, you know to overcome these, you know, having access to it would be a big one…

Participant: Yeah

Interviewer: …that would overcome that barrier

Participant: Yeah, I don't think there would be like a stigma or anything around it. Like: Ohh, why are you using VR before it? You know, I don't think, I don’t think there would be an issue like that I think. Yeah, all the kind of, if you're trying to improve yourself, I think players in your team, coaching - anything that you can do, even if it's 1% to benefit yourself, I think they would allow you to do

Interviewer: Yeah

Participant: So yeah, I don't think that would be an issue. Yeah, I think it's mostly, like we said, the affordability, accessibility, things like.

Interviewer: Yeah. And then if there are any negative attitudes, you think that education about the effectiveness and application um would help overcome these barriers. Is that right?

Participant: Yeah, I think so.

Interviewer: Yeah. Yeah, cool. Um and then, you said it hadn't really been discussed among your team members and, or your coaching team, but you think they would be open towards using it?

Participant: Very much so, yeah.

Interviewer: ..and wouldn’t really be negative. Cool. Do you think.. Yeah, I think that's it for like the virtual reality. I think we've covered all the aspects unless you have anything else you'd like to comment on?

Participant: No, I think that's pretty good.

**Participant 2**

Interviewer: Before we get started, do you have any questions at all?

Participant: Um no, but I don’t know much about virtual reality.

Interviewer: That’s okay. That’s okay. We’ll just have a talk about it, I can explain…

Participant: Yeah. Okay..

Interviewer: So can you tell me a bit about – what do you know about it? Or…

Participant: Umm I've just seen it kind of like Ronaldo and people use it sometimes, um.. and. Yeah, I, I like - is it where you put the mask on and stuff?

Interviewer: Yeah, so you put a headset on

Participant: Yeah

Interviewer: And then you get immersed into a virtual environment

Participant: Yeah

Interviewer: Um and then you can, um, like it’s very immersed, immersed environment

Participant: Yeah

Interviewer: Like you can practice, like where you view the pitch

Participant: Yeah

Interviewer: Or you can be exposed to competition conditions so like with a crowd in the background

Participant: Yeah

Interviewer: You can hear like noises and stuff..

Participant: Yeah. Yeah, yeah, I think it would be good for like positioning and stuff like that, because you can't always in training get kind of game environment so, yeah, that would be good.

Interviewer: Yeah... Can you think of other ways it could be used for football training?

Participant: Ummm I think definitely like tactics and positioning wise cause sometimes you just have a cone as a person, so if you have an actual person kind of thing - then you know where to go. And that's something that I struggle with quite a lot, especially if you're playing in a new position. If the coach is like “Ohh [removed for protection], you're playing for defence today, or like right-back” and if I wouldn't know kind of what positioning. If you do have virtual reality you could see like: this is where the teams setting up on Sunday, this is where we want you to be kind of thing so I think that would work well.

Interviewer: Yeah, that's definitely um a way that virtual reality could be used.

Participant: Yeah

Interviewer: You can also, as I said, get immersed into matches and competition conditions… Can you think of any ways you could potentially use virtual reality um to improve your performance on game day?

Participant: Umm if you said you've got noises and stuff, maybe like - if you're going into a big cup game and there's going to be a big crowd. Like make sure you practise with them crowd noise and like people booing at you and stuff, I guess.

Interviewer: Yeah, absolutely. Um so um if you were to, if you had the opportunity to implement virtual reality training into your regular training. Do you think you would be open to implement it, or can you see any barriers with it?

Participant: I definitely would. The only barriers I could see is that I think it would be quite expensive and hard to set up, which I’ve would put on the questionnaire.

Interviewer: Yeah

Participant: But I think it would be really beneficial and like I've seen with football they're moving more into kind of like… I don’t know how you say it but like statistics wise on things. Like I went and did like this whole fitness test and they did so much analysis on literally like the centimetres I was jumping on the floor and like all of that and like to do with my sprinting, and there was just so much information. But I think the more information you have and those types of things, the better footballer you'll become. So I definitely would try it, yeah.

Interviewer: Yeah, that's, that’s a really good point. And that’s another thing with virtual reality. You can sort of um tailor the training environments and the specific training to each individual athlete.

Participant: Yeah

Interviewer: So say you needed some um training in like scanning, or like positioning

Participant: Yeah

Interviewer: Then like each individual virtual environment could be tailored to you, so that is a really good point.

Participant: Yeah

Interviewer: Um so you mentioned that um barriers you see, like it would be expensive and hard to set up.

Participant: Mhm…

Interviewer: Are there any other um barriers or disadvantages you perceive?

Participant: Hmmm.. Not sure… Just like, as, if you said, if it's in an individual thing as well, if you've got a team of like say like 20 girls training - it might be quite hard to be like, to give everyone 20 different sets and be like: Right, you're off kind of thing. I think it would have to be like maybe a group of like two or something do the virtual reality and everyone else do something else or something… Or come in separately cause otherwise, yeah, that'd just be too many people.

Interviewer: Yeah, absolutely. Um but that’s another good thing, you can take the headset home with you so you could practice um, you know, the mental aspect of the game at home.

Participant: Yeah

Interviewer: Like in your living room because once you put the headset on you are sort of immersed into this environment.

Participant: Yeah

Interviewer: So that’s another potential of using virtual reality. Um so you mentioned um that it could be a little hard to set up perhaps.

Participant: Yeah

Interviewer: Are there any things in this virtual environment that would need to be, you know, accessible, or um the ease of use. Are there any of these factors that would make you more likely to use it?

Participant: Umm I think if my club provided me with one, then yeah, I would 100% use it

Interviewer: Yeah

Participant: But if I had to buy one, that was like, I don't know, 5000 pounds or something, then no, I wouldn't use it.

Interviewer: Yeah

Participant: So yeah, I think each club.... If they had like let's say 5 headsets that they could give out each week or something

Interviewer: Yeah

Participant: Then I think the majority of girls would want to use it.

Interviewer: Yeah, absolutely. So very much about like the accessibility and affordability of it that you see as the barriers?

Participant: Yeah. Yeah.

Interviewer: Cool. What about some of the potential benefits… So you mentioned positioning and also practising for game day if you’re playing a cup where there will be a lot of crowds.

Participant: Yeah.

Interviewer: Are there any other benefits you can think of?

Participant: Maybe practising free kicks... Cause like you could see where the goalkeeper is and like if you're from this angle, you need to do this kind of shot, if you're in this angle need to this kind of shot. You just practise that over and over and over again I guess with virtual reality.

Interviewer: Yeah

Participant: Penalties, and as you said with the crowd and everything would be a good one to do. Ummm.. What else? Yeah, I don’t know. I’d say my main thing would be positioning, but I'm sure other people would have other things as well.

Interviewer: Yeah, so definitely positioning for you.

Participant: Yeah.

Interviewer: So how do you think it could help you improve your performance on game day? So it seems like you are talking more about just your overall performance, but how could it help you improve on game day?

Participant: Ummm… I think… Again, like, I, I, what I do at the moment is I watch like players like Haalaand cause he's a striker and like see his positioning. But then if I could do it myself like watching within like the virtual reality, it might be better cause I'm more of like a kinaesthetic learner, so if I'm doing something I'll learn it better than if I'm just watching TV or watching Haalaand do the runs. So yeah, I would like to be in this virtual reality thing, have the players around me, and then my coach, or whatever says this is the run you need to do and then I literally do it myself. And then in games when I see those players in that line up that we've already looked at because we know their line up so we've set it up on virtual reality then I could just be like - do it in real life. So.. Yeah.

Interviewer: Yeah, that a really point and definitely how virtual reality could be useful.

Participant: Yeah.

Interviewer: So you did mention that you watch videos..

Participant: Yeah.

Interviewer: Is that something you do um with your coaches and your team at the moment and to look back at your performances?

Participant: Um yeah, we watch our past performances and then – well, what we've been doing with this team, I've only been here for like 2 weeks. But um we watch the other team that we're playing against - their recent game, see how they've set up and then we'll kind of do the tactics based on that. But then with my previous team we did watch, we actually watched our own performances. So he had like all of our names listed and then three key points that he'd stopped in the video that he wanted to show us. So we'd go up to the screen one by one, and he'd be like : “OK [removed for protection], these are your three points” and show me the videos and show me what I did well, and what I needed to work on. So yeah, if you could do that within virtual reality - like he could be like: “[removed for protection], you didn't do - you turned out here when you shouldn't have in this scenario”, but then he put it in virtual reality and then I was there what would I do instead? So I was literally, yeah… And then maybe I would pass back, or I'd check my shoulder or whatever and that scenario.

Interviewer: Yeah, that’s a really good point. And that’s definitely what you can do with virtual reality – cause you can manipulate situations and variables and change things…

Participant: Yeah.

Interviewer: That’s a really good point. So um in regards to the use of virtual reality has it ever been discussed among like your team members, either here or in [removed for protection]?

Participant: No, never.

Interviewer: Nah... How do you think their attitudes would be towards the use?

Participant: Just that it's expensive, I think… And that they wouldn't give it to girls, they would give it to the men.

Interviewer: So do you find there's differences in the way you get resources and stuff um?

Participant: 100%, especially in [removed for protection]. Yeah, cause… Well, I don't know really about [removed for protection] because obviously their men's are lower down, but I used to play it for [removed for protection] and their Men's are in League 2. Um and we didn't really get any resources from them to be honest. Apart from like our kits and stuff, but we just… We played once in their ground, but I wasn't there, and then the rest of the time we played on this AstroTurf for our matches and things. But I know [removed for protection] who are also in our league, they've got a lot of support from the men's and fans, and they actually had 28,000 people come and watch the game.. Yeah, against my team, but I was over here so I couldn't play it. But yeah

Interviewer: Oh wow

Participant: It was a really good turnout and that's just like… That's a club that's really pushing for Women's football so I wouldn't be surprised if perhaps they were the one of the first ones… Kind of that type of club to bring in more resources for the girls, such as virtual reality.

Interviewer: Yeah, right. So what about with your um coaches… Um have they, have you spoken, have they ever mentioned anything about virtual reality?

Participant: No, never.

Interviewer: No… How do you think their attitudes towards it would be?

Participant: Ummm I think, I think it again, if someone was like: “Here’s 5 headsets to use on the girls” and everything, they'd be like buzzing by it. Cause they'd want the most resources they can for us because they struggle to get resources for the girls most of the time.

Interviewer: Yeah

Participant: But if they were like: Ohh yeah, you have to pay, as I said, 5000 pounds, or whatever or find your own money, they just wouldn't be able to.

Interviewer: Yeah.. So all about accessibility and affordability?

Participant: Yeah.

Interviewer: And that’s unfortunately one of the barriers, but it is becoming much more accessible

Participant: Yeah.

Interviewer: Um to, like, you know, affordable

Participant: Yeah.

Interviewer: But also um, just like, you know, easier to use and stuff.

very much. Most teams now have got... I know they never used to have it, but you know those little pods you put in the back of your vests um and they measure like your heat map and your max speed, amount of tackles and things you've done.. But that like a couple of years ago that would have never happened, but now it's…. I’ve only really seen it last season and this season clubs, like a base… Most clubs are doing that in the League I was playing it at in [removed for protection]. I don't know about this league, I don't think [removed for protection] do it but yeah.

Interviewer: That's another good um measuring tool

Participant: Yeah, yeah.

Interviewer: To help you see how you're performing and stuff. Cool.. Um do you have any other things you can think of, how you could use virtual reality? We’ve covered the main ones such as positioning, preparing for gameday, um, and like inserting crowds and crowd noises and stuff.

Participant: Yeah… Um.. Maybe like your, I don’t know how to say it but connection or relationship with other players when you're playing.. Like if you come into a new team and you say like, you need to play off like the winger, or something and you haven't played with them before.

Interviewer: Mhm

Participant: Like instead of testing all that out in the actual matches you can test it out before so you can build that connection - so that when you're in games you already have that connection. Instead of just coming into a new team then like you don't know where they play and everything.

Interviewer: Yeah. Yeah. That's a really good point. Thank you so much - That's sort of all I have about virtual reality.

Participant: Okay cool.

**Participant 3**

Interviewer: To start off, can you tell me a bit about what you know about virtual reality?

Participant: Umm yeah, look I don't really know too much. I don't really have... I haven't done it personally

Interviewer: Yeah

Participant: Um, but I mean, I suppose I just know that it involves and putting you in a place where you believe that, you know, is relative to where, you know, where, I suppose, you wanna be, and you know in a way that, you know, can make your body feel things, you know, in the sense of living in that reality that, you know, isn’t, isn't in today's life. So yeah.

Interviewer: Yeah cool. So can you think about how it could be used um for football training?

Participant: I suppose it could help mentally. Like I think mentally it would help a lot, put you in different scenarios through your head

Interviewer: Yeah. Yeah.

Participant: Try and you know...Like I can see how, you know, if you actually feel like that you're there in your mind, like I suppose I could see how it'd help football in that sense. Because just like any sport it's very mental. Um like sports come down to mental, just as much as it is physical, really, so I can see how it would help in that scenario. So as much as you have to do just as much as the physical side, because I mean at the end of the day your body's gonna be out there, but yeah, mental wise I think it would be a decent idea. So…

Interviewer: Can you think of any um ways it could help you improve your performance? Like any specific um ways you could use it?

Participant: Yeah, I can see the um pressure side of it so like, you know, I suppose and you know when you come um to making better decisions, sharper decisions, quicker decisions, you know, that's what it comes down to, I suppose. Especially late in the game when you're tired and stuff and your brain’s not working as quick. You know, you might have that, that advantage because you've been in this scenario before - something like that, you know. Um so yeah, I suppose like through quicker thinking is probably where it's gonna help, I think.

Interviewer: Yeah, so um really use it to sort of immerse yourself into a virtual environment of a football field and then improve your decision making?

Participant: Yeah

Interviewer: Yeah

Participant: Yeah, I think that's probably, yeah, the best way it would definitely help so…

Interviewer: Yeah. Can you think of any other ways it could be used in football training?

Participant: Umm.. I think, I think it's hard to um say other ways like it'll help because, like I said, obviously sports come down to like your physical side as well.

Interviewer: Yeah

Participant: But yeah, purely like I said, mental side – pushing yourself through certain physical attributes, so pushing your brain further than you thought you could go, I suppose

Interviewer: Yeah

Participant: Um, you know, through like running, strengthening those kinds of things. Um it would just trying to help you push through those moments I suppose is where it's gonna help, not just decision making.

Interviewer: Yeah

Participant: But yeah, kind of pushing your brain further than where you thought you could get, maybe on the pitch so..

Interviewer: Yeah cool. So um what are your thoughts about implementing it into your training?

Participant: I don't umm... I mean, I don’t… For me, I mean, I’m like pretty old-fashioned with most things, but um I don't think it's a necessarily bad idea. But the last thing I would want is, if I was a younger player like would be to just, be using that a lot more than actually being out of the grounds and stuff

Interviewer: Yeah

Participant: Cause there's nothing more you want to do than go to the football and actually play yourself, you know. Like I can see if you're at home and stuff and it's like, you know, if you're an inspiring footballer and stuff. And I can see it being like a homework kind of thing in the sense that if you want to become better, it's something that you should do at home rather than take up actual training hours to do it.

Interviewer: Yeah

Participant: Because I feel training hours, you know, you need to be there with your team and actually experience it all at the same time. Although, you know, I'm sure there's ways you could do it all together at once on virtual reality anyway. But, you know, being together and being that comradeship as a team is, I think, where it might be a little different. So, yeah, in that sense, I feel that I would prefer to be more out there than virtual reality, but I suppose using it, like you know, your spare time to try and focus on things that where you know you could be better.. Um pressure moments, key decisions, things like that I think would be good. So…

Interviewer: Yeah. Cool. Um so if you had a chance to use virtual reality, as part of your training, cause it is never meant to replace physical training…

Participant: Yeah

Interviewer: …but more as an addition. Would you be open to using it?

Participant: Um.. I don't know if I would, like me personally, where I'm at now, probably not.

Interviewer: Yeah

Participant: Um maybe if, you know, this was more of a thing when I was coming through younger, possibly.

Interviewer: Yeah

Participant: Um but probably not now. Um, like I, I think I'm pretty set in my ways and I don't, I don’t think there's too much more I can go on that part of it. I think more so with me it's definitely my physical side is where I've gotta try and focus now and keep it good, rather than my mental side. But um, but yeah, I definitely think, not just myself, but knowing younger players and stuff, I definitely think it's a good way to go in the sense of trying to be strengthening their minds and everything like that. I think it'll be a good key thing to add to football, I suppose so. Um, you know, especially if they’re all in and around football and stuff, and at academies and all that. Like um, yeah it could definitely be something that can be added to it. So for sure.

Interviewer: So can you tell me of some of the barriers you see of using it, and why you would not necessarily implement it in your own training?

Participant: Um, like I said, I think it, I think age-wise depends as well. Um, like I wouldn't want, I don't think I'd want to start it too young, but also, I, I feel like it needs to be, you know, as a collective decision that you need to make for your team and stuff. You can't just have, you know, a couple players using it and a couple not. Um, but yeah, like I said, I think I can see it working well in the sense to grow your abilities in football and not just, you know, not just football but in all sports. But um, yeah and just key timings and stuff, so um.. Me personally, like I suppose if I had a younger crowd I would probably try and do it, um or, you know, with professionals, yeah, I definitely could see that and trying to push for that kind of stuff, so.. But at the end of the day I still think, especially football, it’s a.. It's a quite an older age game so they're very stuck in their ways with certain things so trying to push for that might be always a tough thing. But I mean, I can see where you could get benefits from it for sure in the sense of mental wise, so..

Interviewer: So can you comment on- you mentioned the mental side a lot

Participant: Mhm

Interviewer: So decision-making and making quicker decisions…

Participant: Yeah

Interviewer: Can you talk about any other ways it could be used? You said something about pressure…

Participant: Yeah. Well, I feel like, you know, we’ll talk about those pressure moments and stuff, so you know, your free kicks, your set pieces, you know um, penalties - those kind of things. Obviously, your bigger pressure moments, you know, where you have to step up and take them. Um and, you know, no matter how many times you do it in training, it's never the same as when it comes to the time and the actual game. So I suppose preparing yourself for that I think in virtual reality would probably be better than doing it at training. You can't emulate that atmosphere or anything like that, you know, or that moment when you're at training. Um, whether virtual reality could obviously do that a better, like point of that

Interviewer: Yeah

Participant: You know, trying to emulate an actual game scenario, you know, where it's later on in the game, like 93rd minute, and you're tired and all this and you got a penalty to win the game or something like that. So I suppose I could see that working really well um for the pressure moment. So I think that's a really big and key thing for the virtual reality thing for work, I um, yeah, I could see that working really well for pressure moments, so…

Interviewer: Yeah.

Participant: Yeah

Interviewer: Cool. So you mentioned you would not necessarily be wanting to use virtual reality in your training mainly because of your age, and you know, you have your ways of training and preparing for games.

Participant: Mhm

Interviewer: Are there any other like drawbacks you see, or barriers of using virtual reality for training?

Participant: Yeah, um, yeah so like I said, like I, yeah, purely for me, I think it's just like where I'm at.. I wouldn't see myself doing it from now. Um, but yeah, I think the things that could stop you is purely time. Um so depending on in what um area you are in life, I suppose.. Like for myself, I'm a semi-professional footballer with a full time job so, you know.. If you're also getting additional time to do this virtual reality, you know, that's less time you have away from the rest of your life, like, you know, other things that you want to do in life.

Interviewer: Yeah

Participant: But you know, if you're a professional footballer, like that’s an extra hour a day isn't going to change you because, you know, that'll be part of their regime and things like that so they've got plenty of things they gotta do. Um, but I suppose it's just how committed you are and where you are in your life to how that would fit, I think is the biggest thing as well. You know, like I said, trying to, cause like you were saying, you have to do the physical side still. So then you're pretty much adding on the virtual reality. So I think it's more so the timewise to try and, you know, emulate these moments and stuff in the virtual reality world to make it beneficial for yourself, so.. Yeah.

Interviewer: Yeah. So for you it's mainly a matter of time and you have so much physical training and other stuff going on in life?

Participant: Yeah, for sure. I think it's just hard to… You know, life is always busy and moving and I think it's just hard to try and fit everything in. So I think that's where I might struggle with like semi-professional footballers. Um but same sort of thing, it depends what everyone wants to get out of it, um and how much further they want to push their game. Um but in the sense of like being younger and wanting to actually be a better footballer it would definitely be very helpful I think in that sense. But yeah, it's, it’s more so time and, yeah, like things you are just.. And that's where I think it'll be the struggle is like, like I said, mainly just for semi, semi-pros, because yeah, it's just that, it’s just that gap where it's like, it's not quite all your time into football um whereas the professionals it’s obviously all about that kind of lifestyle. So yeah.

Interviewer: Yeah. Can you think of any ways you could overcome these barriers um to the use?

Participant: Um I think the way to overcome it um… I think schools can be a big part of it, um depending how young, I suppose your age group is same sort of thing. But you see schools these days have football academies and things like that.

Interviewer: Yeah

Participant: So, you know, instead of teaching um a whole class just physical every single time, you could do a virtual reality thing, or even if you give them the homework to go home and do that and put them in these scenarios. Um especially if you're running football academies, like you know, and I think that would be not a bad idea tom instead of just send them home and you know, um say work on this work on that, you know, like you can make them do that, I suppose, but in a virtual reality world, um and I suppose you do that same at a club level when you get higher out of football, out of school, sorry

Interviewer: Yeah

Participant: And you know, same sort of thing, you give them these progression steps that they wanna take, if they want to become better and I suppose the coach will be: These are the things you need to work on, so whether or not it's, um it comes down to that player then to then become better at that. So you know if they don't show any improvement, it's kind of their own decision but, you know, most players that are in these kind of levels want to get better so if a coach can pinpoint exactly where they need to improve, I think a virtual reality can help with that. So they just need to, you know, go home and I suppose do that, or find the time to then work on those specific things. So…

Interviewer: Yeah. So that's another benefit of virtual reality that you can individualise these um training environments.

Participant: Mhm. Yeah, yeah, for sure.

Interviewer: So it sounds like overall you have um quite positive attitudes towards virtual reality and you do see the benefits, but there are some barriers when you are a semi-professional player, or a player at a lower level um with time and, you know, life responsibilities. Is that right?

Participant: Yeah, for sure.

Interviewer: And um has the use of virtual reality ever been discussed among your team members?

Participant: Uh, no, actually not at all. Certainly not at my level. Um, I can't, yeah… Yeah, most, most players I've played with and stuff um not really something that we've spoke about. Um, but yeah, like I said, I think football is a very more of an old-fashioned sport, um in the sense that they don't um… Well certainly not in the clubs that I've been around they have, they don't even think about using the kind of technology in that way. Um, but yeah, I haven't... It's definitely not something that I've heard talks of with my teammates or clubs or anything like that I’ve been to so.. There's other ways, you know, that we do data and stuff, but certainly not virtual reality wise.

Interviewer: So it hasn't been discussed with you coaches either?

Participant: No, not at all, no.

Interviewer: How do you think their attitudes um or thoughts about using virtual reality would be?

Participant: Personally, I honestly think most coaches wouldn't wanna go for it.

Interviewer: Yeah

Participant: Um, and I can, yeah, and I definitely see why.. Um.. Yeah, I think, I think most coaches probably aren't gonna go for it because it'll take their players away from them actually coaching them at the fields and, you know.. Coaches always like to, um, I suppose it’s way, in the ways that they coach them, they can get, I think they can get, they can get mental and physical out of them out of a pitch rather than the virtual reality side. Um, so for example, if they, you know, had them at training, they could run them through these scenarios over and over, which is then, you know, triggering their minds to get better at it, but also their body is adapting to these scenarios as well, physically. So I think that's where they think they can probably get a better edge. Um, but also because, like I said, they want them there at the grounds and I don’t think most coaches, I think most coaches probably won't be too on board with the virtual reality. That's like, most coaches I played under, yeah, I definitely think that will be the case.

Interviewer: Yeah right. So you think they'll be more inclined to just want their players um do the physical training?

Participant: Yeah, I think there'll be a bit of pushback if they were, you know, asked about virtual reality. Um, yeah, I definitely think most coaches would probably go against it.

Interviewer: Yeah. That's a very interesting point. Um so just to um sum up… The ways you see virtual reality could be the uh most beneficial are through um training decision making and um maybe a little bit of tactic, like, you know, where to go on the field..

Participant: Yeah

Interviewer: … through immersion. And then also through preparing for high pressure moments.

Participant: Yeah

Interviewer: Um are there any other ways you can think of?

Participant: Um, not really. I, I think, um, yeah for those kinds of moments, like, it’s, they're, they’re your biggest moments. Um, you know, in, in the sense of football, like um, mental wise and that… You know, cause it’s, and that’s, I honestly think um, the more I think about it, the more I do think that it would be very helpful in that mental state. Um, you know when you can't always be at the pitch, you know, that's where, that's something where it would probably be good, if someone is injured, for example.

Interviewer: Yeah

Participant: Um so I could bring that in… So it would be good for a club to have, you know, a player who's injured, can't be out in the field, so you put him through this virtual reality, so, you know, in his mind he's still training. Obviously you still have to do some sort of physical to keep some sort of fitness, but you know, if you can't do most things out on the pitch then that's one way they could probably adapt to that. Um, but yeah, like I think, I think mental wise the big pressure moments is where it's going to help the most

Interviewer: Yeah

Participant: And just repetition in the same, same areas where I said, like a player can improve, or should improve.

Interviewer: Yeah

Participant: That's where I think it's going to help them just going over and over and repetition to become better at those kind of areas. So um, yeah, I think mental wise is where it will really help um rather than anything in those key points.

Interviewer: Yeah. Cool. Um just before we finish off this part of the interview, do you have any additional thoughts um about the use of virtual reality that we haven’t touched upon?

Participant: Um, yeah, not really, just that last one, like saying, you know, when a player is, you know, sick or away or things like that, um injured..

Interviewer: Yeah

Participant: Like that's where I think it probably could become a very, more of a positive, um because, you know, you don't always have access to the fields and whatever else. Same thing for wet weather and whatnot, so they’re probably times that you could try and use those kind of things, so yeah.

Interviewer: So say you became injured, would you be um interested in using virtual reality?

Participant: I, I think so. Yeah, I think um, if I was injured um, and I really wanted to get back out there and that, I, I think that would be a good key thing to kind of get you back. Um, but like I said, at the end of the day obviously it's still a physical injury so you still need to get over it.

Interviewer: Yeah

Participant: But you know, as long as you keeping your mind active and about football, you know, at a similar time that, you know, training is on so you're still kind of, you know, living in that part where, you know, you're still at football and stuff, and keeping that brain active and keeping up with what you should be. Um, you know, if a coach could pass on what he's working on at that training night, you know, you can then try and um re-enact or emulate that in the virtual reality and, you know, try and keep up with him so you don't lose anything in that, you know, in the way of tactics or things like that so you're not so far behind the rest of the team when you come back.

Interviewer: Yeah, absolutely. That's a really good point. How about - you did say virtual reality could be good for preparing for pressure moments… Given you are the assigned penalty taker

Participant: Mhm

Participant: Um would you be open to using virtual reality for preparing for these?

Participant: Ummm probably not, probably not.

Interviewer: Yeah

Participant: Purely because, like I said, I think I'm at an age where I don't wanna try anything new.

Interviewer: Yeah

Participant: Um, you know, I don't know how much longer I'll be playing semi-professional, um, but I'm…. It's, it’s not something that I'm willing to try and change now

Interviewer: Yeah

Participant: I've got my set way and how I do it now, um, and yeah I just, I.. The last thing I wanna do is try and change how I do it, you know, it kind of ruin it in that way

Interviewer: Yeah

Participant: But I definitely, like 100% for younger kids, I could see how it would definitely help, if you start from a younger age. Um, or even if clubs do it, um with, yeah, younger players um as they come in and stuff, you know, and already decide who's gonna be their free kick taker and their penalties, and they can see how these are, they can see how their players are in those moments in the virtual reality and then build on them so…

Interviewer: Yeah

Participant: So yeah, I definitely think it could help to try and recreate those moments in games, because I mean in football it happens, it happens all the time so yeah…

Interviewer: Yeah. Perfect. Yeah that's a really good point. Um and it does sound like you are very positive towards virtual reality and the technology – um, just not for yourself because um you are at a later stage in your career?

Participant: Yeah.

Interviewer: Yeah

Participant: Yeah for sure.

Interviewer: Yeah. Cool. Do you have anything else about the use of virtual reality?

Participant: Uh, no, that's pretty much it. I think we touched on everything that you can think of.

**Participant 4**

Participant: So with virtual reality, is it, like what does it actually do? Because I haven’t done it before…

Interviewer: That’s okay. I was going to ask you about what you know about it, but I’ll give you a brief summary.

Participant: Yeah. Yeah.

Interviewer: So essentially it’s a virtual simulator that you can enter, so you put on a headset

Participant: Yeah

Interviewer: And then you um, you can be immersed into a training environment, or like, you know, a performance environment. Um so you can see like, you know, a field, um could be like, just like a training field or like a stadium

Participant: Yeah

Interviewer: And you can insert lots of different variables. So like different opponents, and um a crowd behind the goal, you can insert noises

Participant: Yeah

Interviewer: Um, and you know, you can sort of – depends on what you wanna to train in it, um but you can sort of insert many different variables.

Participant: Okay

Interviewer: Does that make sense?

Participant: Yeah, makes sense.

Interviewer: Cool. Perfect. So um I was gonna ask you – do you know anything about virtual reality?

Participant: I know about virtual reality, but not much to do with training.

Interviewer: Yeah

Participant: So I know what it is, but ..

Interviewer: Can you tell me just like the base of what you know?

Participant: The base of what I know about it… Well, essentially, from my experience - I've played games using it..

Interviewer: Oh cool

Participant: Soo… Yeah, just put the headset on, you're in there. You've obviously got the controllers or whatever and the motion and just play games

Interviewer: Yeah cool

Participant: So it's pretty cool. But I haven't used it for training at all, no.

Interviewer: Yeah fair.. Has uhh… Can you think of how it could be used for training?

Participant: Ohh I could picture like…. Relating to football, it's like um, you could put yourself in high pressure situations, cause when you're training you can't have like 10,000 people in a crowd, like watching your train, obviously. But obviously with virtual reality, I'm sure you could put those variables in that you wouldn't be able to get on a day-to-day basis training. Where...

Interviewer: Yeah. Can you think of other ways?

Participant: Other ways to use it…. Uhh maybe if it's like really bad weather outside, you could do it - cause it'd be an indoor thing.

Interviewer: Yeah

Participant: So it could replace it so you're not missing out on a day's training because of the weather….

Interviewer: Yeah

Participant: So you could use it for that. Not sure what else you could use it for too much.

Interviewer: That’s okay. So do you think it could help you improve your performance in any scenarios?

Participant: Well, I wouldn't know till I tried it but I'm sure in specific scenarios it would be good to use, you know, because you’d be able to do stuff you wouldn’t be able to do in training with it.

Interviewer: Can you talk about some of the things that you could be able to do, or you picture you could be able to do?

Participant: Umm maybe more so…. Like, I’d say, like free kicks. Even games, having a crowd, you know, let's say that yelling at you, giving you… Being nuisance, and even.. Maybe making certain parts realistic where you'd have… Like, do you know, set, set pieces, like a free kick, let's say

Interviewer: Yeah

Participant: You could put a full man wall that would jump and a goalkeeper… That's properly going a goalkeeper, maybe you wouldn’t be able to get that in regular training.

Interviewer: Yeah, yeah, yeah. And do you think that could improve your performance during a game?

Participant: Definitely – yeah, I think it could.

Interviewer: Yeah. So that exposure to these variables that you can't insert in training you think would be beneficial?

Participant: Yeah, definitely would be - mentally beneficial so yeah….

Interviewer: Yeah… Um so if you had the chance to use virtual reality, in as a part of your training, because it would never replace the physical…

Participant: Yeah. It would never …

Interviewer: Would you be open to using it?

Participant: Yeah, definitely. I'd be open to using it and trying it. Seeing how it works. So I think it would be good

Interviewer: Yeah

Participant: But.. wouldn't know till I tried it.

Interviewer: Yeah. Yeah, of course. So overall you think it's a pretty good tool?

Participant: Yeah, I think definitely could be, yeah.

Interviewer: Cool. Perfect. Are there any specific things that you would need virtual reality to be able to do to use it?

Participant: I think you'd have to be… Because obviously you'd have to kick so having it realistic in a way… So, you know, what I would do, let's say in real life, would have to be able to be replicated in the simulation, as in…

Interviewer: Yeah

Participant: Because I'm not sure how it works, is it physical ball you kick?

Interviewer: Yeah, so you would have a physical ball um, and then you would have a headset on… So of course you're a little bit restricted in that aspect

Participant: Yeah

Interviewer: But you'll be immersed into like the environment that you're training in.

Participant: Yeah

Interviewer: And then you'll have a physical ball to kick and you'll be able to see the ball move in the virtual environment.

Participant: Okay. Yeah so obviously if it's like, I guess, clean whereas, where the ball moves, you can see it on the headset, there's no sort of lag I guess between what you see here and what's happening down there.. Yeah, so definitely.

Interviewer: Yeah. Yeah. Do you see any potential like barriers or disadvantages of implementing this?

Participant: Uuuhh obviously like what I just said before, maybe it's not fully realistic, so maybe something, if you're training like that and then you get to the game and it's a bit different… It can affect your performance in, I guess, the real world by using the virtual reality training

Interviewer: Yeah

Participant: Barrier, probably getting to use it. I don't know how many of these VR things are out there for the training, I assume… Would you know?

Interviewer: Umm there are headsets out there. I think it's just like a question about accessibility and affordability.

Participant: Yeah…. Affordability. And yeah accessibility, that's probably one of the biggest barriers, but I guess as technology improves, they'll get easier as it comes.

Interviewer: Yeah

Participant: So that's probably the biggest one, I'd say.

Interviewer: Yeah. Yeah. And of course like, you know, it's getting bigger and bigger

Participant: Yeah

Interviewer: …and as it develops, it becomes more accessible

Participant: Yeah

Interviewer: But yeah, nah, there’s definitely lots of virtual training environment and stuff out there. Umm what are the major benefits of using virtual reality?

Participant: Major benefits… Probably the biggest one when it comes to training is…. Putting, getting game like situations in training.

Interviewer: Yeah.

Participant: Because training and games are two completely different things. You know, you haven't got… I guess factors of, I guess, pressure as well, and you people watching.

Interviewer: Yeah.

Participant: There’s obviously a lot more on the line when you're playing a game rather than training. You know, if you miss, no one really cares

Interviewer: Yeah.

Participant: If you do it in a game it's obviously a bit more of a big deal

Interviewer: Yeah.

Participant: When it’s… Then there's risk on the line, I guess, you know.

Interviewer: Yeah… So really getting put under that pressure in a training scenarios you think would help you?

Participant: Yeah, definitely cause I think…. If you wanna get to a high level in a sport, you gotta be able to be put on the pressure, you know.

Interviewer: Yeah.

Participant: You know, there's a difference between just playing with your mates, no one's watching, and then playing in a stadium where there's thousands of people, watching and yelling and screaming

Interviewer: Yeah absolutely. Yeah, nah, that's a really good um point… Um.. And has it ever been discussed among like, the use of virtual reality in a training environment among your teammates or anything?

Participant: Nah, I haven't really heard... I don't think anything at all of it. So.. Until you kind of mentioned something to me, I didn't really know people were using it.

Interviewer: Yeah right

Participant: But I can see how it would be beneficial and I looked… I didn't do a lot of research but I looked a little bit at like how it could help and I was like: Okay, interesting.. But nah, has never been discussed.

Interviewer: Yeah, wow… Not about – with your, with your coaches either?

Participant: Nothing… I guess it's kind of new coming in. And my coach is pretty old school, so I don't think he’d be down for that… But maybe he would, I don't know.

Interviewer: Yeah, right.

Participant: Yeah.

Interviewer: How do you think um your teammates and your coaches, for that matter – you said your coach is a bit old school.

Participant: Yeah

Interviewer: But how do you think your teammates would think about the use?

Participant: I don't… I'm not too sure. I feel like it might be… Like I said, in specific, I guess, scenarios where you'd be able to replicate stuff that you couldn't do in training that you'd need, I guess, it to be a game-based situation - it'd be good more for like mental pressure - that'd be the biggest thing.

Interviewer: Yeah

Participant: Cause I don't think there's really anything you'd be able to do in virtual reality that you couldn't do in real life besides adding factors that apply pressure onto yourself.

Interviewer: Yeah. So overall the biggest benefit you see would be like sort of like the mental preparation for high pressure moment?

Participant: Yeah, mental prep... Yeah, exactly.

Interviewer: Yeah, cool. And just to sum it up - you don't see any like big barriers… Like the biggest barrier would be the differences between a virtual world and the real world?

Participant: Yeah, I think that would be the biggest barrier… Cause at the end of the day, even still… When you're probably in, immersed in it, you still know that it's not real, it's still a simulation. So like all those factors that you'd want to get from a game. Like I’d say, like a crowd, the pressure of - you have to score, you couldn't really replicate that unless it was an actual game fully.

Intrviewer: Yeah

Participant: But you'd be able to…. It'd be a lot better than just training with no one around.

Interviewer: Yeah

Participant: So… That's probably the biggest, yeah.

Interviewer: So you… Definitely see the benefits of it?

Participant: Yes. Yeah, absolutely. Could see how it would work

Interviewer: Cool. Um and the biggest like barrier towards using it at the moment is mainly like the accessibility?

Participant: Yeah, accessibility. Probably affordability…. I'm not sure how much it costs, but definitely - cause I haven't heard of it, so I'm not sure what’s around…

Interviewer: Yeah.

Participant: …how much access you get from it and how it works. But I'd say that's probably the biggest one.

Interviewer: Yeah. Cool… And if you had the chance to use it, you would um be willing to use it, given it had the benefits that you see, such as the pressure?

Participant: Yeah, definitely. I think… 100% if it was.. what I think it would be, because I'm not too sure. But if it would do what I think it could do, then it'd definitely be beneficial, especially for high pressure situations.

Interviewer: Yeah. Cool, perfect. That’s sort of everything I have about virtual reality.

Participant: Yeah

Interviewer: Do you have any other thoughts or comments or anything towards the use of virtual reality in training?

Participant: Ummm I don't think it could ever fully replace training… Like....

Interviewer: Yeah.

Participant: Cause… I just don't think it could ar the end of the day. Even if it got really, really good, I still don't think, yeah, it would be able to fully replace it.

Interviewer: And it wouldn’t um ever replace physical training

Participant: Yeah

Interviewer: Cause you know of any elite sport, or at least like where there’s some kind of endurance involved um

Participant: Yeah

Interviewer: it’s.. you need the physical aspect, but it's just to aid with the mental training.

Participant: Yeah. Yeah, supplement your training, yeah.

Interviewer: Yeah, and also, you know, if you have an injured athlete, they can still be immersed into this environment.

Participant: Mhm..

Interviewer: Um but yeah, that’s really good points… Thank you

**Participant 5**

Interviewer: So to start with, can you tell me just what do you know about virtual reality?

Participant: Um, I mean not a lot I would say, like I have some... I mean, I'm not sure about, so I have some patients that they kind of use and they talk to me about it - about the things that they do. I know they have some simulators and you can simulate like, you know, a lot of driving, um

Interviewer: Yeah

Participant: But I never even thought about soccer, when you said it, I was like: Wow, that actually would be pretty cool

Interviewer: Yeah

Participant: Uh, but yeah, I don't understand how it works or how you could use it, but I think the idea of it is pretty cool…

Interviewer: Yeah

Participant: Cause if you think like, I'm just thinking about, you thinking about the game, and if you have… We always get told to visualise, but if you can actually visualise and physically see it, I think that that would be pretty effective.

Interviewer: Yeah

Participant: So I think, yeah. But I don't, yeah, I don't know much about it, but I can, like when I think about it, I was like, it makes sense.

Interviewer: Yeah. So you haven’t um done anything with virtual reality at all?

Participant: Nah. Never. Never.

Interviewer: Nah… Um so can you tell me, like, you say it’s hard to visualise how you could use it in football training

Participant: Yeah

Interviewer: But can you tell me some of the ways you do think it would be beneficial in your training?

Participant: I think just, ment.. Like, cause you know, if you're preparing for a game you are meant to like visualise, you know, what movements will you do, what sort of things you need to do, game positioning, so you think about the training is like: Okay, we're doing this movement, I need to be here, I need to be there. But if you can physically like think about it and see where yourself is going to be on the field, that would be sick. Like, you know, that would be so cool. So kind of just, yeah, like in a game sense, in like… Set pieces in a sense as well, so I think yeah, just recreating that moment so I think it's pretty cool.

Interviewer: So sort of for mental preparation would you say?

Participant: Yeah absolutely, yeah, yeah.

Interviewer: Yeah, cool. And how do you think that would improve your performance during a game?

Participant: I think it's kind of, you know, if you think about it, the more you practise, the readier you have for any situation.

Interviewer: Yeah

Participant: So if you're practising and, you know, you would expect outcomes from it, then I think it would just improve, cause you know, you're reacting, you’re not just thinking, you're just reacting because you know that that could happen. So I think you, your actions will be easier because you're not really thinking, you're just reacting, so it's just natural

Interviewer: Yeah

Participant: …if that makes sense.

Interviewer: So it could sort of help you like, the way you anticipate the movements of your opponents?

Participant: Yes

Interviewer: And maybe decision-making?

Participant: Exactly, absolutely, yeah, yeah, yeah.

Interviewer: Yeah, cool.. So a lot of mental aspects. Um can you see any, um, like with your motor performance, or like, you know, your drills, or anything – could you?

Participant: I mean thinking about like um mental connection to the brain is like the same thing as like, you know, if you're practising the moment, like it’s just that sort of that signage through your body, it will be so much easier. So like if you're doing drills and things, so yeah that’ll be, that’ll be interesting to see like how would that affect in like.. I don't know. Thinking about like, yeah, that, that, I think that that could definitely improve, absolutely.

Interviewer: Yeah

Participant: You still need to do the physical part of things, but yeah, the mental I think it will be easier, cause you know, you're already thinking so when you do it, it will be a lot easier

Interviewer: Yeah, yeah, yeah

Participant: So yeah, I do think that that could, yeah, that will be very interesting…

Interviewer: Cool

Participant: …if that could happen.

Interviewer: Yeah. So overall you think quite positively about um the use of virtual reality?

Participant: Yeah, absolutely. Yeah, I think that that could be very, very beneficial, absolutely.

Interviewer: Cool... Um so if you had access to using virtual reality, as part of your physical training, cause it would never replace it.

Participant: Yeah

Interviewer: Would you be open to using it?

Participant: Absolutely, yeah, absolutely. Especially if you start seeing results and things, yeah

Interviewer: Yeah, yeah, yeah

Participant: Yeah, I think I would definitely be open to it, absolutely

Interviewer: Cool…. So would a virtual simulator need to have any specific features for you to use it?

Participant: In what aspect do you mean, like features like?

Interviewer: Um like would you need to, like would it need to be able to simulate game situations, or like, you know, put a crowd in the simulator, or…

Participant: Yeah, I think so. Like the more realistic to the game scenario, I think, the better. Cause then you're literally like just in the zone and, I think, yeah, that that would, yeah, that that would be amazing

Interviewer: Yeah

Participant: Cause then you're just replicating what you expect in a game.

Interviewer: Yeah, yeah, yeah

Participant: So that'd be pretty cool.

Interviewer: Cool. So a lot about the immersion?

Participant: Yeah

Interviewer: Um, would, um, are they any um, like do you see any barriers to the use of virtual reality in your training?

Participant: Like the mental fatigue of it…. So like, if you do too much and then you don't have enough time to process, and then you’re actually mentally tired for the game. So I think that that will be a barrier. So like knowing how far can you push

Interviewer: Yeah, yeah, yeah

Participant: Especially before a game. So you might use it as a preparation during the week, but before a game, might be too much. Cause you know, you can be mentally tired, and that can be worse than physically tired. I think that that'll be a barrier. Uh another one, I don't know, I’ve heard that people sometimes you get sick doing like, motion sick, depending on things like that, so I think that could be something. I don't know how it'll be in a soccer scenario, comparing to other things. Um so yeah, definitely not something that you do before a big game, I would say

Interviewer: Yeah, yeah, yeah

Participant: You know, introducing pre-season and get used to it, um like anything else. Um so those would be the main thing, just like how do you expose there, how often, and I think that's gonna be more the barrier. And then yeah, I don't know… I don't know visually if this causes you, you know, being close to the screen or things like that, I don't, I don’t know if that's any impact or not, if it affects in your eyes.

Interviewer: Yeah, so there could be some differences between like the virtual world and the real world.

Participant: Yeah.

Interviewer: Yeah, yeah, yeah. So with mental fatigue aspect of the use it, um can you see how that could be overcome? You said, like just using it um, you know, leading up to a game, but not just before…

Participant: Yeah, yeah.

Interviewer: Um are there any other things you can think of that could sort of overcome that limitation of using it?

Participant: Ummm… I think just the exposure to it, I don't know, it's like with fatigue, you know, if you're training with fatigue, you eventually build the resilience. I don't know if mentally that's exactly the same thing

Interviewer: Yeah, yeah

Participant: But that's what I would expect. So I think that, the more exposure you are, then the better you get at it. So that would be kind of similar… Umm, yeah, I don’t know.

Interviewer: Nah, but that’s definitely a good point, you know, you don't just go running um a marathon, like you sort of build up…

Participant: Yeah, so I don't know if it's the same with the mental aspect, but yeah, if you think about that principle, then I would think that, yeah, just like the gradual exposure and then just, yeah... And then I think having a training plan, like you're not just gonna do for the sake of doing, but like, what is your purpose by using it.

Interviewer: Absolutely

Participant: So I think that can be more effective so you're not just, you know, using it for the sake of using it.

Interviewer: Absolutely. Um so what would be some of the benefits of incorporating virtual reality into your training routine?

Participant: I think just being mentally ready, or like, you know

Interviewer: Yeah

Participant: I think everyone deals with games differently, so like, I feel like the more experience you have, you have more experience cause you've been exposed to more scenarios. I just wonder if you can get the young players that are not experienced to be readier mentally using virtual reality, that will be interesting. Just because they're being exposed to those scenarios virtually, even though they haven't in the real life, but when you replicate that in real life, will they, will they feel more prepared for it? So like, get a young player or like a young Matilda, she's like eventually gonna play a World Cup and she's, you know, you'll be shitting her pants before getting on the field, but if mentally you've been exposed to that in the virtual reality, will that make it any easier?

Interviewer: Yeah, yeah, yeah

Participant: So those could be things that I think it could be beneficial

Interviewer: Yeah

Participant: But yeah, I don't know. That's kind of what I think that it could potentially help with

Interviewer: That’s some really good points, and that is the sort of the benefit of virtual reality, as opposed to real world training, like because you can simulate all these environments like international stadiums and crowds and noises…

Participant: Yeah

Interviewer: …and you know, positioning and…

Participant: Because it's all about how it makes you feel, and that's the thing, you feel that, and you shake and you're like… Where if you're being exposed to those emotions, it’s like, your brain's thinking, you're there, so it's like, is that something that's going to prepare you when you go there, so you're actually like: Oh, yeah, I got this, I’ve been exposed to this before so that would be interesting. Some things there.

Interviewer: Yeah, nah, really good points. Um has the use of virtual reality ever been discussed among your team members, or your coaching team or anything??

Participant: Never, ever, ever. I don't even think I haven't heard about any, like we do, I feel like the older I'm getting, the more like use of videos and, you know, different sort of like.. A lot of more visual stuff instead of just the board and things like that, or just on the field. And I think that just the visual, seeing what you're doing and, you know, putting thoughts and things has kind of make it so much easier for you as a player. But I think like, I don't know, it's just a completely different like world that would be like… I think that will, could be something that could be a game changer for a lot of people.

Interviewer: Yeah

Participant: But yeah, no one ever, ever, ever spoke about it.

Interviewer: Yeah wow. So you do a lot of video training um now?

Participant: Yeah, yeah, like, yeah, analysis, and yeah, personal ones and collective ones, we always do, like at least once a week.

Interviewer: Yeah, wow

Participant: Yeah, just like in the specifics, so you do one preparing for the game and then you do one after the game. So it'll be 2-3 times a week at least

Interviewer: Yeah, wow

Participant: And then at least 20-30 minutes every time. So you just get videos, clips, and photos, and just to see what you're doing, what can you do better, like what did we do good as a team, what we didn’t. So yeah a lot of, yeah, which is good

Interviewer: Yeah wow… So do you think virtual reality could be used um as an addition to this kind of training?

Participant: I think so, yeah, I would think so…

Interviewer: Yeah. Cool… And um how do you think that your teammates would think about virtual reality?

Participant: I don't know, I guess it depends… Cause it is such a new thing, like, I don't think we've been exposed to it so I don't think a lot of people will know what you think of it.

Interviewer: Yeah, that makes sense.

Participant: That's kind of the main thing. I think some people will react like positively towards it, some others will be like: Well, what is that going to do for me?

Interviewer: Yeah

Participant: So yeah, I'm actually unsure. I think some positive, like in a positive way, some others, yeah, not so much.

Interviewer: Yeah, yeah, yeah. How about your team, coaching team. Do you think they would be open?

Participant: The ones that I have now, I don't, I don’t know. If you do, if you do give them like some research and say: Look this can help, then they would use it.

Interviewer: Yeah

Participant: But if it's something new and you just wanna introduce, I don't know if they'll be a bit sceptical about it.

Interviewer: Yeah

Participant: It depends…. But if you put some evidence in front of them, I'm pretty sure they'd be like: Yeah, let's try.

Interviewer: Yeah. So it’s all about the evidence and whether it can help to improve performance?

Participant: Yeah. Yeah, absolutely. Cause yeah, cause I think it's, especially like, they already, you know, you’re always under the pump, you need to perform, so, like, I don't know. Like you need to sell it really well, the idea, for them to buy in, I would say, ccause it's like: Oh, let's just try this to see if it's gonna help, but at the same time it's like: Oh we don't know if actually works or not, so they would be like: Oh, do I bring something that could make them worse or something that, that’s not. They're going to spend time on and then they're not going to get any benefits from it. So that’s always like, the juggling

Interviewer: Yeah, yeah, yeah

Participant: Cause we don't have a lot of spare time because unfortunately a lot of us are still work, so it's kind of, yeah, so it's just the hard part of things. But yeah, I think it depends. Sorry it’s not a really clear answer.

Interviewer: Yeah, no that's completely fair and it's hard when you haven't had any experience with it before, like even from your perspective, it seems like you are quite positive towards it

Participant: Yeah

Interviewer: Like implementing it and trying it out, but when you haven't tried it, it’s a little bit harder.

Participant: Absolutely, absolutely. But yeah, I don't know, I'm always, I don’t know, I feel like that everything is evolving. So I'm always like: Oh, yeah, that sounds like a really good idea. But yeah if it works or not, you know…

Interviewer: Yeah, yeah, yeah. Alrighty, so I just wanna sum up everything we've spoken about.

Participant: Yeah

Interviewer: So overall you are quite positive towards um the implementation of virtual reality into your training?

Participant: Yep

Interviewer: And the areas you see could be most beneficial are sort of for like decision-making, um anticipation and for mental preparation for a game.

Participant: Mhm.

Interviewer: Um the barriers you see are um the like mental fatigue that could come as result

Participant: Yeah

Interviewer: Um and also perhaps some motion sickness and some differences um between like the virtual world and the real world.

Participant: Yeah

Interviewer: Um but um overall, super positive and open to using it. Is that correct?

Participant: Yeah, absolutely. Yeah, that is correct.

Interviewer: Perfect. Do you have any other thoughts or comments towards the use of virtual reality?

Participant: Uumm… I don't think so. Not that I can think of right now, I'll probably go home and think about some.

Interviewer: That’s okay. Perfect. Alright, we’ll move onto the second part of the interview to talk about your experiences of performing under pressure…

**Participant 6:**

Interviewer: You said you haven’t really done virtual reality before, can you tell me just what you know about it?

Participant: Umm look I suppose… Not sure about this. Um so what I know about virtual reality, it’s, you know, it’s supposed to give you like a similar type of effect um except it's not the real thing, but in a way, it sort of does the job, I guess… I think…

Interviewer: Yeah. Nah, you you definitely have the right ideas. So essentially you put the headset on

Participant: Yeah

Interviewer: And then you get immersed into an environment

Participant: Exactly, yeah.

Interviewer: and that will be like, you know, on the field, or during a match or during a like specific situation and then then you can insert different variables. So you’re definitely right…

Participant: Um.. That would actually be pretty cool. Wow…

Interviewer: Um, so um have you used it at all, not for training, but have you used it for gaming or anything?

Participant: Nah, never. Never used it, unfortunately.

Interviewer: Nah… Can you tell me about how you think it could be used in football training?

Participant: Umm.. I reckon… Obviously uh we love to play out, you know, but sometimes, for example, when it's bad weather

Interviewer: Yeah

Participant: You know, that could come in handy indoors. So if something like that was set up indoors, I think it would, it would be perfect for using it in that way.

Interviewer: Yeah

Participant: You're still on top of your training, you know, in a way, and you're still getting something out of it. Um, yeah so I think virtual reality will come in handy if indoors if like um… If there's bad weather outside

Interviewer: Yeah

Participant: So I think that's one way to actually use it, yeah.

Interviewer: Yeah, absolutely... Can you think of how it could be used to improve your performance?

Participant: Um.. Yeah, I mean…. Essentially, you’re sort of doing the same thing. Uumm I think it's… So like you're sort of doing the same thing and I think in a way you're still getting out what you need to get out if you're, if you're doing training outdoors, for example.

Interviewer: Yeah

Participant: Um you’re still thinking the same, you're still moving the same. So yeah, I think it will be quite effective in that way.

Interviewer: Yeah… So you can um sort of um do different things, so you could like get inserted in an environment where you were taking a penalty

Participant: Exactly, yeah.

Interviewer: or practising passing the ball, um or, you know, heading drills… Can you talk about how you think any of these things could be effective?

Participant: Yeah. Again I think if those, if those variables were inserted and you're doing what you need to acquire, I think in that way I think it, it'll improve you because initially you are doing the same thing that you would do outside, outside of it so... Again, just the longer you do that, the more I guess you'll get better. I don't think there'll be any much difference

Interviewer: Yeah

Participant: In terms of like improving cause you're initially just doing the same thing - isn't it?

Interviewer: Yeah

Participant: But obviously it depends with um the variables and everything

Interviewer: Yeah

Participant: And yeah, so I think it will be very effective in that way.

Interviewer: Yeah. Can you think of any situations or skills where it would be more effective than others?

Participant: Like football skills?

Interviewer: Yeah

Participant: Um I think like a... For, for example, I, a, what do you call it? A player who plays in the midfield, for example.

Interviewer: Yeah

Participant: You know, most of the time he needs to work on like scanning, you know, locking his shoulders... So I think for virtual reality

Interviewer: Yeah

Participant: I think that will come in handy for someone like a midfielder just because he is required to do a lot of things, and obviously if it he can't do that outdoor, he can do it indoor

Interviewer: Yeah

Participant: with virtual reality. Yeah, he can… I’m pretty sure for a midfielder, yeah… I think… Like for example scanning, like just to see like just the type of pressures that's around him in, in virtual reality, I think that’ll come in handy and then when he's outside, he's sort of like used to it in a way.

Interviewer: Yeah

Participant: And yeah, I guess it could improve that way, really.

Interviewer: Yeah. Can you think of other ways?

Participant: Um that… Um only like penalty kicks, free kicks even… Like set pieces. Umm.. Goalkeepers, you know - why not?

Interviewer: Yeah

Participant: Um so I don’t think just to do with shooting and like passing - all the typical normal stuff you'd see in football I think it would, I think it will be like just a good fit, really

Interviewer: Yeah

Participant: If everything was to come in -if all those variables were to happen.

Interviewer: Yeah... So overall, you think um it could be pretty useful in your training?

Participant: I reckon, yeah…

Interviewer: Um so if you had access to it, um of course it would never replace the physical training, it would just be an addition...

Participant: Yeah

Interviewer: Would you be open to using it?

Participant: I definitely would, yeah… I definitely would. Um, you know, but I just have to be careful that… I feel like I would enjoy it too much, cause, you know, it's so much different in terms of um like pressure. Like you're obviously yourself in that game. But you know, in, when you're outside, there's so much going on, I feel like… Um you know, there's pressure from everyone, you know - actually seeing you, actually telling you what to do but rather like when you're in virtual reality it's like about you type of thing - it's about improving yourself

Interviewer: Yeah

Participant: without any pressure around. You know, you fail - like I'll go again. And um, yeah, you’re just in your own world, you know. But obviously if you're outside and there's people looking in. It won't, it won’t be the same… Like you're just too... It's just too much pressure, I guess. I don't think, I don’t think you'll improve in a way. But when you're alone, I guess, and you're doing virtual reality I think you'll just do it more until you actually get used to it. You're just like playing video games where, you know, the more you play, the better you get, I guess…

Interviewer: Yeah

Participant: I think that’s what virtual reality could sort of, you know, help with

Interviewer: Yeah… So really help you get used to the conditions and improve your skills?

Participant: Exactly, yeah.

Interviewer: Yeah. Cool… So um if these features were available within a virtual environment um for you to train outside your physical training - would you implement it as part of your training routine, you think?

Participant: Yeah, I definitely would. I definitely would. Yeah, in my spare time. You know, I mean, someone like me who really loves football. Like, I play FIFA, so I would probably take that time to get into virtual reality.

Interviewer: Yeah

Participant: Um and like just cases during off-season as well, you know - because there's not much training going on off-season.

Interviewer: Yeah

Participant: So I would definitely implement that as well

Interviewer: Yeah

Participant: In my off-season training, just to get better… and yeah, like it helps a lot of things like when you don't have people to train with, you could use that as well. So, yeah, it's something that

Interviewer: Yeah

Participant: I would, you know, get into it, definitely.

Interviewer: Yeah… So it sounds like the benefit you see are being able to train off-season um to improve your skills and just getting used to the conditions?

Participant: Exactly, yeah.

Interviewer: Are there any other benefits you see of using virtual reality?

Participant: Umm.. I think, I think the big thing is it can be done anywhere, anytime type of thing, you know. I think that's the biggest thing and… I think just in general just to improve with your game, you know. I guess that's the two biggest um, you know, benefits.

Interviewer: Yeah.Yeah. So improvement, and then training anywhere at anytime?

Participant: Yeah.

Interviewer: Um do you see any um barriers of using virtual reality, or challenges with the use of virtual reality?

Participant: Umm… That's a good question. Uh… I'd like to think there would be, um you know, I think… Again, obviously it's not as close as is, as it is if you're, if you’re doing it in real life.

Interviewer: Yeah

Participant: Again um, um, you know, I don't think, because as footballers, when you when you are training, you know, sometimes you need… Like for example, sometimes you need someone to actually, um like coach you, or whatever, you know. You, you're always gonna need that second opinion of someone when you're training. And I think for uh virtual reality, you don't really get that, you're just your own player. So you're thinking this is what I need to improve on, but it's always important to get that second opinion where someone can see what you're doing

Interviewer: Yeah

Participant: And like this is what you need to improve on.

Interviewer: Yeah

Participant: And I guess that way you become a better player, I guess.. I guess that’s one negative, yeah.

Interviewer: Yeah… So do you mean sort of like a lack of feedback?

Participant: Yeah, that's it. Yeah, lack of feedback, lack of feedback. Um unless obviously there was a way where you're in virtual reality and someone else can see what you're doing is with you

Interviewer: Yeah

Participant: If someone else can see what you're doing, then I suppose that will sort of be good, I guess. But yeah I guess with just virtual reality when you're training on your own, you're on your own, and sometimes you need that second person to tell you what you're doing…

Interviewer: Yeah

Participant: To tell you where you're wrong, where you're going wrong, where you can improve on, just that feedback.

Interviewer: Yeah… So in the established football virtual training platforms there are, you know, if you're training a specific skills, say your passing or your scanning skills, they can sort of give you feedback and say how you go.

Participant: Oh, they do? Ohh, that's brilliant. That's brilliant. I see.

Interviewer: Yes, so you can sort of like see how you're going and if you're improving… Of course, it's a computer

Participant: Computer, yeah

Interviewer: …and not a person, but it does give you feedback and information of how you’re improving, or how you're performing relative to your past performances if you have done it before.

Participant: Oh well there you go… Never mind then

Interviewer: So there is um feedback if you are using one of those ones.

Participant: Oh wow, that’s brilliant. That's brilliant. That’s pretty cool.

Interviewer: Yeah, it is pretty good… So no other benefits you see, or barriers sorry.. Barriers you see?

Participant: Um… I don't know. I can't honestly think of one, you know… It's different when I haven’t. Like if I did, if I had experience with it, then I would probably say more than I would. But, I have no experience with it, so yeah. I've got nothing at the moment.

Interviewer: Yeah. Absolutely… Cool. That’s so understandable. Has it ever been discussed among your team members to use virtual reality?

Participant: No actually, no no.

Interviewer: No?

Participant: No, it’s just something we see on TV, hey. Like, you know, you’re watching TV and think yeah like that would be pretty cool. But you know, it's sort of just... The add was there and that's it, when it's gone, it's gone. It's not talked about anymore…

Interviewer: Yeah…. Can you think of what they would think about it, or do you know what they would think about it?

Participant: Um… Just another, I guess, just another way of having fun, I guess, you know

Interviewer: Yeah

Participant: Obviously there would be like those, those benefits, but I think for most of us, it would just be like… Just, yeah, just tough one really, another tough one really.. Umm.. because, I guess at this very moment, it might feel like it's not needed in a way.

Interviewer: Yeah

Participant: Like for example, I guess, when Covid happened and everybody was out of football, I guess, if that, if that was like a thing

Interviewer: Yeah

Participant: If everybody had virtual reality, then yeah, it would definitely be good. I think everybody would be using it, you know, because you know, everybody missed football. Everybody missed going out, except for, you know, everybody missed the competitive side of football. So I guess virtual reality would have sort of helped with that, I guess, I reckon, um yeah.

Interviewer: Yeah. Yeah, that's fair... And it was actually used by the Premier League players who have access to all these things..

Participant: It was? During Covid? Oh wow

Interviewer: Yeah, they used it during covid to, you know, be able to at least maintain their skills while away from the field…

Participant: Ooohh it must be nice… Must be nice

Interviewer: What about with your coaching team – I guess they haven’t um discussed anything about virtual reality with you either?

Participant: Not the players, not the coaches.. All the players and the coaches are quite split…. Um nah, nah. just that, it's never been discussed about just in the team in general or amongst the coaching staff, I think.

Interviewer: Yeah

Participant: But honestly, I think it would be pretty cool, just in general having it. You know.

Interviewer: Yeah. Do you think your coaches would be open to um their players using it as an addition to the training on field?

Participant: Um… I, I don't see why not, you know. Um, yeah, but then again I think it depends on like what type of coach it is. You know, if it's a coach that's, you know, pretty… Who bases his playing style on back in the days, he's going to want the real thing, you know…

Interviewer: Yeah

Participant: You know, like.. But obviously if it’s a young coach, I guess, then he would say why not, you know.

Interviewer: Yeah. Absolutely.

Participant: But yeah, I guess it just depends on the type of coach it is, really. Um yeah.

Interviewer: Yeah, absolutely. Cool…. So overall it does sound like that and you are very positive towards virtual reality

Participant: Oh yeah.. Oh sorry go on, sorry-

Interviewer: And just open towards using it if you had access to it?

Participant: Yeah, definitely, I’d definitely give it a go.

Interviewer: Yeah

Participant: Yeah, 100%. I think just in general, anything that has to do with football I would give a go. Umm and yeah, it's just another way to sort of improve your skills, I reckon.

Interviewer: Yeah

Participant: So yeah.

Interviewer: Cool… And the main use and benefits you see are really just like being able to practice away from the field and at any time, um an also being able to improve your skills?

Participant: Yeah.

Interviewer: So you mentioned that it could be really good for a midfielder to sort of improve their scanning ability…. Are there any other skills you've thought of later on?

Participant: Um, yeah, I guess for example, every sort of, every position requires different things.

Interviewer: Yeah

Participant: So for example, midfielder – scan, could be scanning, for a defender, because I'm a defender

Interviewer: Oh yeah

Participant: So for a defender could be like, you know, heading the ball away, clearance, you know, that comes in the box and just clear out. Clearing out danger really..

Interviewer: Yeah

Participant: You know, um playing out under pressure or whatever. I guess for strikers, yeah, just to score, I guess

Interviewer: Yeah

Participant: So I think there's lots of benefits for different power positions, so I think would be good in general, yeah.

Interviewer: Yeah… So overall it’s just like of great benefit it sounds like?

Participant: 100% yeah…

Interviewer: And you don't really see any barriers um towards the use?

Participant: At the moment, no - I don't, I don’t see a barrier really. That would, that's really like very effective that would be like: Okay this is not good.

Interviewer: Yeah

Participant: Um but yeah, for me I think it would be a good addition to um, to support my training, I guess.

Interviewer: Yeah, yeah, yeah… Ohh, that's great. Um before we move on to the second part of the interview, do you have any other thoughts or comments or questions in regards to the virtual reality?

Participant: Mmm nah. No, no, not at the moment…

Interviewer: No, that's Okay…. If anything comes up later on you can always just ask me later on.

**Participant 7:**

Interviewer: Can you start off by telling me, what - do you know anything about virtual reality and what do you know about it?

Participant: Umm I don't know a whole lot. I mean I’ve only seeing like bits and pieces, maybe online and stuff, but obviously like see that there's normally a headset that kind of is meant to immerse you in some sort of virtual scenario.

Interviewer: Yeah

Participant: And that's probably about it, yeah. Like I probably know more about it like in like video games and stuff

Interviewer: Yeah

Participant: I know it’s meant to kind of situate you in a certain situation. But yeah, not a whole lot to be honest.

Interviewer: Nah, that's okay…. Have you had any experience with it um in training or in video game scenarios?

Participant: Not really to be honest - it's not something I've ever used.

Interviewer: Nah

Participant: Definitely not from a training in like my sport, sport perspective. But yeah, not even video games. I've never actually experienced it, no.

Interviewer: Yeah. Nah, that's completely fine. Um can you think of how it could be used in football training?

Participant: Umm probably to put you in certain situations of like stress, or certain situations that are typically difficult to emulate. So like a lot of the times, like in training, it’s, you know isolated events or in like a training scenario where there's no kind of outside pressure. I mean sometimes in training you'll do things, or games where it's like, there'll be rewards, so then you get a punishment if you lose and stuff so that adds a bit of pressure.

Interviewer: Yeah

Participant: But it's hard to get that real environmental pressure of like being in a stadium or um in an environment where there's like people yelling out and stuff like that so I think there could be a space for it there

Interviewer: Yeah

Participant: Because that's really difficult to emulate

Interviewer: Yeah absolutely

Participant: from like a training perspective on a field. So yeah, I could probably see it there, um but yeah, I don't really.. Yeah, know what else

Interviewer: That’s okay…. So being put in these situations in a virtual simulator. How do you think that could help you improve your performance on the field?

Participant: Umm I think… Yeah probably just exposing you to those, to those niche kind of experiences, like I was saying, like being within a stadium under pressure - if you can emulate that in kind of a virtual reality world um and try and get players to perform tasks, then that could be… So that when they actually experience it, if it is like a grand final, or like a, or a cup game where there's a lot of pressure - if you can emulate that and transition and get those players to be exposed to it earlier, then they're gonna be a lot calmer on the day

Interviewer: Yeah

Participant: when actually undertaking that task and that high pressure situation, yeah.

Interviewer: Yeah, yeah. Yeah. So mostly about like, you know, getting the exposure to the situations that you may be exposed to in a game later?

Participant: Yeah, yeah. I feel like that’s when you, when you, when kind of get stuck as a player - if you're trying to do something for the first time, or even if you're playing out of position. Um you're not used to kind of the movements and what that position requires so.. For any event that you're undertaking if it's unfamiliar to you, you're probably likely to perform worse if that makes sense.

Interviewer: Yeah

Participant: So I guess exposing yourself to that higher pressure situation in training could correlate to better performance on the actual day.

Interviewer: Yeah absolutely

Participant: Potentially… That's my thoughts anyways.

Interviewer: That's cool. That's good points. Um do you think it could be used for anything else during training or in preparation?

Participant: Um.. Yeah, I'm not really sure. I mean - can you… With these VR set-ups, can you… Like do you actually play with the ball, like?

Interviewer: Yeah. So you'll have a ball and usually you'll wear like some monitors on your feet so when you kick the ball, the ball actually moves as if you kick it.

Participant: Right… Okay. Yeah um… Yeah. I’m not really too sure, to be honest. I'll have to experience it first. But I mean just putting yourself in a situation - whether it like, even just from a training point of view, if you can emulate kind of positional awareness or like if there's some sort of uh tactical thing that the coach wants the team to do, and if you can emulate that in a virtual reality scenario. So although you can do that on the field as well, it's like, it could just be something that players could use, like at their at home or something even just to kind of go through and walk through that pattern if it's recorded or something - I don't know. Umm.. But yeah, of the top of my head, I'm not 100% too sure to be honest… It hard when you haven't experienced it yet.

Interviewer: That’s fine. It is super hard when you haven't experienced, but that is like some of the benefits and things that you can do in virtual reality. Like it's used a lot for like improve your scanning and your decision-making on field - again because you can get exposure to it and, you know, training in your own time.

Participant: Yeah, yeah. Yeah. Yeah, yeah. And I think that like those points you just made there, like decision making and scanning, particularly with football it’s a huge thing depending on where you play. If you're a midfielder, you need to have 360-degree view, right so then you know everything is, so scanning is so important. Yeah, it could be just refining little things that you may have done during the day on the training field. But then if you can incorporate that into some sort of virtual reality, or even have that on the screen so that coaches can see what the players are seeing and then talk about what they could do better or potentially improve upon and that, I don’t see why that wouldn’t be beneficial, yeah.

Interviewer: Yeah. So you think that could be used um for improvement?

Participant: Yeah, yeah, potentially. Because a lot of the times it's like, if you’re... I know myself as a player you can be watching a game from the sideline, whether it be if you're injured, or if you're on the bench and then when you're on the field it’s very different…

Interviewer: Yeah. Yeah.

Participant: … to actually being there in the positions. So I think even if you could somehow kind of, yeah… Make it work so that the coaches or people external could see what it's like on the field. Because then they could have their perspective or their perceptions about what that player should have seen in this pass but maybe that player was looking at something else or… I don't know - I see how it could be used and it could kind of bridge the two between coaching and like the player themselves. So yeah, potentially.

Interviewer: Yeah. Nah, that's a really good point. Again - it is hard when you don't have any experience with it but just like with the information that you do have… So if you had access to using virtual reality, of course as an addition because it would never replace the physical training. Would you be open to using it?

Participant: Yeah, I don't see why not. I think any athlete or player that wants to improve themselves will always try other avenues, but I think yeah for sure. Like I, even just play FIFA like on the PlayStation. I think there's times there where it's, you're still correlating that to training and trying different formations and things and…

Interviewer: Yeah. Yeah.

Participant: You're always thinking about how this can kind of relate to performance. But I mean, I feel that virtual reality would be a lot more um realistic than playing FIFA as an example. So I don't see why players wouldn't be open to it.

Interviewer: Yeah. Yeah.

Participant: Yeah, I definitely think it's probably a space for it somewhere there.

Interviewer: Yeah, cool. So you mentioned like exposure to pressure and situations you aren't usually in and also decision making and scanning. What um things would be the most beneficial, or would you find the most, like the best in a virtual simulator?

Participant: Probably.. Probably that… I would think that that like scanning, decision-making, like in the game is probably the most important thing cause it's what happens the most, like the amount of touches you'll get in the game, depending on what your position, but particularly like a midfielder, if I use them as an example, you're probably looking at... 100 passes a game, and every one of them a decision has to be made, they have to scan. So if you can refine those moments within a game

Interviewer: Yeah.

Participant: …those players are gonna get better, they're gonna improve, they're gonna have better performance for the of the team, essentially. I think those moments are probably like key if there was a way to improve the performance of those key, key things

Interviewer: Yeah

Participant: and things that happen a lot in the game. Like I know with the penalty kick as an example, it's a high pressure situation but it's pretty rare, if that makes sense.

Interviewer: Yeah

Participant: Like it's something that you're only gonna, like in a, even in a normal league game the penalty kick, it's… there's a bit of pressure on, but there's not as much pressure as if it's like a cup final or a cup game, which they're few and far between. You're lucky if you're taking a penalty in a cup final.

Interviewer: Yeah

Participant: Um so while that's important I do... I would suggest that if you, if there was a way to kind of make virtual reality realistic to the commonalities of the games and things like that with what the example I used as a midfielder I could definitely see where it would be used as a tool

Interviewer: Yeah

Participant: in addition to normal training to enhance performance and improve your team outcomes, I guess, for sure.

Interviewer: Yeah, yeah. Cool. So you play as a defender, right?

Participant: Yeah.

Interviewer: Yeah cool. So for you, as a defender, where would you see the greatest benefits?

Participant: I think um… Like as a defender, reading the game is super important, so I could definitely see that, like we were saying, exposing yourself to those situations, if you could put on a headset and be seeing the field and seeing player movements and talking through that with the coaching staff - about what you're looking at, what you should be looking at, like that's a key part of the game

Interviewer: Yeah

Participant: and something I've tried to develop over the years is actually reading the games – is what they'll call it. So if the ball is out on the left hand side of the field, you need to know where your defender is, but you gotta anticipate where that ball is coming to make sure you can either intercept it, or be there - that if he's gonna get it, he's not gonna have time to get a shot off and score a goal.

Interviewer: Yeah

Participant: So umm... Yeah, I think particularly from a defenders point of view, it would be that understanding the game, what's happening, reading it and just putting yourself in that scenario because we can talk about it all week, at training about: Ohh, they're gonna do this formation, or this is what it might be like and you might be able to experience that in training but if you can refine it in a virtual reality scenario where you're adding it and it’s supplementary to your normal training.

Interviewer: Yeah

Participant: I think, yeah, it could be really beneficial in that. Cause you're not going to be able to get the contact, I guess, or the defensive kind of tackling side of it and refining that - that you can leave that to the actual training park. But from your decision-making point of view, or just exposing yourself to that scenario, um I could definitely see it's beneficial there. Because that's probably the hardest thing is in training you're trying to expose yourself to it, but in the game it’s, it is always different because you only get that one chance, and if they, if you mess it up and they score, you're in trouble. So yeah, from that point of view, defending like a decision-making, but then also, yeah, like on the ball as well. So like if you're a defender, you want to be cool, calm and composed and be, and be making the right passes at the right time because you don't want to pass it to your player who's under pressure, you turn it over, then they score. You wanna be able to make the right pass that could be recognising – you’re in a situation, there’s multiple options, but which one is the best one? This is the best pass because he's under pressure, we've got an overload on this side, so we can use our numerical advantage to progress down the left flank. As an example, you know what I mean?

Interviewer: Yeah….

Participant: So yeah, I can see it beneficial from that point of view, yeah, for sure.

Interviewer: Yeah wow - that's a really good point, and very good insight into what you see and how you sort of could see it work as a football player and improve… Um so overall it does sound like you are quite um positive towards the use of virtual reality as part of your training?

Participant: Yeah, yeah, I think I'm… Like I said I’m open to kind of anything that's gonna make you better as a player. And I don't see like, well, I haven't experienced it, but I've seen bits of pieces. I don't see why virtual reality couldn't be a tool to enhance training and performance and things in addition to what you're normally doing. Cause a lot of the time too during the season your training loads are so high that you’re, you're kind of battling with this balance of not training too hard that you're going to injure yourself, but then also you want to get more out of yourself, so it's like a really fine balance of what can you do extra

Interviewer: Yeah

Participant: and this way it's not as such a... I guess a physical, physical like task of running around a field, sprinting, tackling - that you could just put yourself in kind of the reality of playing and get closest to actually doing it um were not having to, yeah, physically be running around - so I think it could definitely be in addition to training and like I would be open to doing it.

Interviewer: Yeah

Participant: Yeah, I don't think I've heard anything bad about it. I mean no one really experienced it, but at the same time, yeah like I've never heard kind of bad connotations or thoughts about it anyway, so.

Interviewer: Yeah, yeah, yeah. No. Ohh that's really good to hear. And so lots of benefits that you have touched upon. Are there any like barriers or challenges you see with using virtual reality?

Participant: Umm potentially the logistics of how it works. Like I'm not too sure if it's just a headset, or if you need to be in a certain room or how it all works. Um.. But yeah, apart from the logistics side, I mean, not really. Aah potentially it's not emulating like game and a game environment if you're wearing a headset.

Interviewer: Yeah

Participant: But I mean, there's always gonna be a.. You're like, there’s always gonna be something that's gonna have to impact. So like you're not gonna wear a headset in a game, but if you can emulate that kind of game reality via a headset then maybe – like it’s a catch-22 you’re gotta sacrifice something to be able to do it.

Interviewer: Yeah

Participant: So yeah, maybe the logistics side of it, maybe that it's not as comfortable, especially if you are moving around in it. But yeah, I'd say probably logistics, mainly… like if you need to, I don't know, do you need to be in a like room to do it?

Interviewer: No, not necessarily a… like you can't really run around with the headset too much.

Participant: Yeah

Interviewer: Because it's like - you don't know where you're going, and you're immersed into this environment. But you may be, maybe not in a room like this, but a bit of a bigger room.

Participant: Space, yeah, yeah.

Interviewer: But, like, you could be in your living room doing it… Because you just put a headset on and then, you know, you could practise the scanning or decision-making, or positional awareness…

Participant: Yes.. Yeah, yeah, yeah. Yeah, for sure. And that's where I think it's probably the most beneficial. So if I picture myself, if I was a professional athlete, and I was at training all day, like we did in my a morning session and afternoon. And I wanted to come home and refine some points like that. Maybe that's something I can just do in my living room, pop on the headset for half an hour and I work on scanning, I work on my decision-making.

Interviewer: Yeah

Participant: That type of thing is where I could see that it would be beneficial. Um I think if it was something where you were, yeah, wanted players to put a headset on and run around the field

Interviewer: Oh no- absolutely not. That would be way too dangerous.

Participant: I think that where it might get difficult, or actually the logistics of it, like players would have to go to a facility, put on all these like, I know it was like biomechanical testing you put all the points on like that takes time.

Interviewer: Yeah

Participant: So those type of things I would say would be a barrier.

Interviewer: Yeah

Participant: But if you can have it where they put on a headset at home, then that's super easy - I don't see why any athlete wanting to perform better wouldn't encourage the, the use of it so…

Interviewer: Yeah, absolutely. Yeah, that's really good point. And also one of the other benefits, you know, like it's just training at a time and place that's convenient for you.

Participant: Yeah, for sure. Yeah, because, I mean, I, like particularly the highest level like they would be flat out training, as they are anyway, so when they come home they may not want to

Interviewer: Yeah

Participant: But I mean for those players who really wanna progress it could be a tool that they use in their like downtime. It's not as, like I said, not as intensive that they're going to be exhausting their body anymore when recovery is so important.

Interviewer: Yeah

Participant: It’s something they could just refine little points with. So yeah I definitely see it could be a tool in that aspect, yeah…

Interviewer: Absolutely. Cool… That's really good. So has the use of virtual reality ever been discussed among your team members?

Participant: Not really in my experience, I think. I think it's something that ah maybe potentially, like it's something that you've kind of always thought of, but like I've never heard of anyone doing it or anything. So it’s not something you kind of labour on… It's like maybe a throw away thought of like: Oh, I'd be cool to do this and then you just go about your day so.. Yeah, not, not really too much that I'm aware of, cause I mean from my point of view, it's not something I've ever experienced or heard any more about until I'm kind of speaking with you now.

Interviewer: Yeah, yeah, yeah.

Participant: So I think if it was available to players, it'd probably be spoken about a lot more.

Interviewer: Yeah

Participant: Like even at the semi-professional level now, I know, it's getting a lot more um data-driven and things with like…. We've got all the four stacks and stuff or like the green bars for like physical anatomical testing and stuff.

Interviewer: Yeah wow

Participant: So that's all being incorporated too. So any kind of way to develop training and things is encouraged. I think if it was available and people were aware about it I think it would probably, yeah, get a lot more discussion around using it and utilising the capabilities of it.

Interviewer: Yeah

Participant: I just don't think it's very well-known at this point in time.

Interviewer: Yeah, absolutely.

Participant: Among like the players anyway.

Interviewer: Yeah, yeah, yeah. But um do you think they would be open towards, like say it was available for you all to use. Do you think your teammates would be open to using it?

Participant: Yeah, I think so. I think um, I think too because it's new originally like everyone would be keen to have a go at it.

Interviewer: Yeah

Participant: Um so yeah, I don't think… Yeah, I don't really think anyone would be like: Oh, I don't want to use it, like there's some sort of conspiracy theory here – tracking eye data… I don't know. But um, yeah, I think, I think um in my experience anyway, a lot of players would be open to it. I think anyway.

Interviewer: Yeah…That's excellent. Um how about your coaching team? Has it ever been brought up? I'm guessing no based on what you said…

Participant: Yeah, yeah… Probably, probably not brought up to my knowledge with the coaching staff. Um but yeah, I'd probably reiterate that coaches are always looking to benefit their players and benefit their team's performance, right? So some coaches in my experience are um, are quite open to new technology, new methodology and things. So they would probably be receptive of something like this, whereas some coaches are quote on quote old school

Interviewer: Yeah, yeah, yeah

Participant: And you rock up to pre-season and you're just doing laps of the field to get fit, where like the other ones are kind of taking that sport science approach and you're kind of periodising programs and things and they're a lot more open to kind of new technology and how it can influence performance so..

Interviewer: Yeah

Participant: I think with those coaching groups you, you'd probably get a lot more um buy in to something like this, whereas some coaches may just say: Ohh no it won't do anything and not interested to listen, to the benefits it can.

Interviewer: Yeah

Participant: But I think to even just from my experience that old school coaching mentality is kind of starting to diminish - all the coaches coming through now have a respect and um and want to learn more about what they can do to benefit their team. So I think, yeah, I think it'd be receptive of, received a lot better by a lot of the coaching staff.

Interviewer: Yeah, yeah, yeah

Participant: Like, they're always looking for ways to improve performance. And at the end of the day, what they want to get their team to win so they look good. So yeah, if there's a way that it could benefit performance I don't see why, why not they wouldn't wanna try - they'd be silly not to.

Interviewer: Yeah, cool - that's a good insight. So do you think your current coaching team um would be receptive towards it?

Participant: Our, like my coaching staff?

Interviewer: Yeah.

Participant: Um… Yeah, potentially… Yeah, potentially.

Interviewer: Yeah

Participant: I think that like they’d be, be keen to give it a try at least, hear out, hear you out if it's an option. Um yeah, I definitely think that, that could be, could be something that'd be interested in.

Interviewer: Cool. Perfect. Alright so mainly just want to sum up everything to make sure I've understood everything you've said correctly. So overall you’re positive towards it and would be willing to use virtual reality?

Participant: Yeah

Interviewer: Um and um the main benefit you see are for exposure to game situations, um scanning, decision-making and positional awareness

Participant: Yeah

Interviewer: Um and overall you think that both coaching teams um and the players that you play would be open towards it?

Participant: Yeah, for sure, for sure. Yeah, all those points I think, yeah, is what I would say, I would feel like just from um my perception of virtual reality and what I know about it anyway.

Interviewer: Yeah

Participant: That they would be the things that um you'd probably most benefit from. And yeah, I don't see a reason why players and coaches wouldn't want to, to have a go anyway and see if it is beneficial and... I mean it's, it’s… you got to try it first, right.

Interviewer: Yeah

Participant: And then if you like it, you don't like it, then that would be your decision from there - if you continue with it. But yeah I definitely see that there's a, an area to give it a go anyway and it could be beneficial. Yeah, for sure.

Interviewer: Yeah, cool. Perfect. Alright so before we move on - just wanna ask if there's any other comments or thoughts or concerns you have regarding virtual reality. Any questions?

Participant: Yeah not really, I think I’m pretty, I think I've kind of - yeah, I kind of have said everything, or my thoughts on it anyway. I just think, yeah, I definitely could see it being used. I just think the accessibility of it would be the biggest probably barrier.

Interviewer: Yeah right

Participant: As in like what we spoke about, the lounge room example, that's probably the biggest thing, I think any, I think anyway, that would, that would um, that players would use, and coaches would probably try and implement - is just making sure, it's obviously accessible, it's easy and it's not gonna kind of take away from any current training regiments - it's just like an addition that they can do and making it easy for the players is the biggest thing. As soon as you make something difficult, they don't wanna do it, yeah.

Interviewer: Oh yeah.. Do you think it would be easy to incorporated as a part of your training, like say you've got a headset provided – do you think it'd be easy to incorporate into your training routines?

Participant: Umm… Yeah. Like easy, yeah - I'd say, yeah, because it'd just be something like, let's say, if there was a headset at the club and I wanted to come do some VR training for half an hour before the session, or after the session - that's easy. I'm already at the club, so I'm just gonna book in, make sure - because if there's only a number of headsets, there's normally 20 people in the team. So...

Interviewer: Yeah

Participant: I think if yeah, it was accessible in that point of view and you could walk in and do it and just yeah, like I said, if I wanna improve my performance, I'm gonna go out of the way to do it. So if it was like that scenario where I could just go in and use the, the headset pre or post training, or even on a, on a, on a rest day - go in and use it then, yeah, for sure, for sure. Cool.

Interviewer: Cool – perfect…. Thank you so much ☺

**Participant 8:**

Interviewer: Can you start off just telling me what do you know about what virtual reality?

Participant: I do… I don't know much.

Interviewer: Don't know much?

Participant:: I don't know much apart from.. Um.. Other than what we use in entertainment wise, but not for training purposes.

Interviewer: Yeah, that's okay. Um so have you had any experience with it in video games or anything?

Participant: Mmm not myself. No.

Interviewer: Not yourself.. Can you think of how it could be used for football training?

Participant: Well I used to do, back in the day, in [removed for protection] some like uh mental visualisation.

Interviewer: Yeah

Participant: So try to live the moment again of like, yeah taking a penalty or whatever, like a situation like.. So from my position, cause I'm a striker, a more offensive player

Interviewer: Yeah

Participant: Would like put myself in like strike, like situation where I could score and, and visualise how I would um.. Like try to remember when I kicked the ball properly, how it felt in my body, what I could see, the visualisation, visualisations in general

Interviewer: Yeah

Participant: And I really like that experience.. Um.. But like, like that, so I would, I would imagine um.. Especially to, in a high level, at a high level you have pressure

Interviewer: Yeah

Participant: And being able to, I mean, you have the example with the World Cup at the moment. I think talking about penalties, everyone is missing their penalties and it's just the group stages

Interviewer: Yeah

Participant: That's the stats I was pulling the other day and it, it went.. I think the first six penalties, only one got scored, and after it was, two got scored on the second penalty, but it got retaken

Interviewer: Yeah

Participant: But like the pressure of that, like being able to like, yeah, work on that cause you can't find this um atmosphere at training. Like it would be very different, so recreate those kind of situations, I think for high performance that could be interesting, yeah

Interviewer: Yeah… So sort of getting exposure to future situations?

Participant: Yeah. Yeah, yeah, yeah.

Interviewer: Yeah

Participant: Stressful situations, um.. Potentially like pressure, like, yeah, like even by yourself. Like a lot of people are doing like one V ones and stuff like that

Interviewer: Yeah

Participant: But when you can actually potentially like include pressure or things like that through VR, um virtual things like, why not?

Interviewer: Yeah, absolutely. Um and some people also use it for, like scanning and decision-making to improve those abilities. Do you think it could be beneficial for that?

Participant: Definitely

Interviewer: Yeah.. In what way?

Participant: I'm gonna say something bad for Australia, but I think that's something that is really lacking in the Australian game

Interviewer: Yeah, right.

Participant: Um.. Especially for younger players. But um where I would struggle in some way like I'm.. Virtual reality is pretty new

Interviewer: Yeah

Participant: And it requires also adapt, adaptation, I think, for the body and the mind to be able to, you know, be like: Okay this is potentially.. Like this is a real situation and it's actually not a real situation.

Interviewer: Yeah

Participant: But um.. Sometimes, sometimes you can't recreate those kind of situations at training. So being able to have this add up to your training, that will definitely be like another benefit… like that, as an extra, that could definitely be like, a yeah.. Another benefit

Interviewer: Yeah.. So just getting, you know, exposure to all situation that you may..

Participant: Yeah… I, I try to see it as a as a striker how I would benefit from that

Interviewer: Yeah

Participant: And situations like, or even as a, as a midfielder when you're in the middle, like you play a 360-degrees game, like it's coming from everywhere

Interviewer: Yeah

Participant: And one of the issues, I would say with women's soccer, is that we don't scan enough

Interviewer: Yeah

Participant: Like if you look at players like Iniesta back in the day and like, the guy, his neck is like this constantly, and being able to read what's happening around you. So practise that through virtual.. Yeah, it, it could definitely be interesting. Yeah.

Interviewer: Yeah. So you think.. How do you think that all of this could help you on field? You said like it could help you to like maybe get better awareness?

Participant: Better awareness and reproduce like it's like repetition. So technical repetition at training, like um, same same, they don't do that much. But like passing repetition in terms of like this technical stuff, you have to repeat it

Interviewer: Yeah

Participant: Awareness you have to repeat, but it's harder to do that in the same.. Like higher repetition, like you do with technical things, it's harder. So definitely, if you can create like a situation, virtual situation when a player is coming and pressure you. Um, how do you adapt? How do you um, how do you adapt? How do you see where your players, like know what you're going to do with the ball before you receive the ball, that's the basic of football. But that's something that you could definitely develop on that. Receive the ball knowing where the players are, where the options are and how you adapt and then re-do that situation all the time

Interviewer: Yeah

Participant: To like, like increase repetition in that would definitely like improve players, the, the ability of players to adapt to situations. I think for me that's the best.

Interviewer: Yeah. That makes sense. That would be a really useful tool, and that is one of the ways that virtual reality is being used.

Participant: Yeah

Interviewer: Particularly in Europe at the moment and like with the English Premier League

Participant: Oh they’re using it there?

Interviewer: Yeah, they are using it already.

Interviewer: If you had access to using virtual reality, as part of your like physical training cause it would never replace it.. Would you be open to using it?

Participant: Definitely. After um.. Like if I see like my three seasons in Australia with the time that we have, and like, you know, like employment situation, that's hard to fit everything in.

Participant: Yeah

Interviewer: But like for full professional players that have the right, you know, facilities and employment situation, that's definitely that.. Personally, I would use that. I would use everywhere, anywhere that I can, like I can benefit, whatever thing that can benefit my training I would, I would you use it and I'm quite a fan of that type of stuff, so I would definitely use it.

Interviewer: Yeah, yeah, yeah. Absolutely. Um so if you could use it what do you think would be the greatest benefit?

Participant: Mm.. For myself it would be repeat situations that um I can't, like I'm struggling with in a game.

Participant: Yeah

Interviewer: Um so what I just said before, um like under pressure, when you receive the ball under pressure, being able to scan and analyse the situation around you, that's one thing I would use it for. And another thing, as a striker would be um.. Like a one V one situation, or with the keeper or um.. How to like um make a quick decision on um how the defender is reacting to you, how the goalkeeper is reacting to you, and where to put the ball to finish. So like in a game, it's like few seconds, it goes very quickly, and you don't have that many, many occasions in the game to, or training, to actually work on that. So repeat the situation.

Interviewer: Yeah. So overall just getting like expose you to the experiences and like through repetition and in scenarios you can't recreate in training?

Participant: Yeah. Yeah, yeah, yeah.. And then... I, for me, repetition is the most important and I’ll give you like a real story about that. When I was in [removed for protection], we have the Cup that happen in a season. So, and Cup games, and back in the day we didn't have extra time, so it was going straight to penalties after.

Interviewer: Yeah

Participant: And so we had a coach that every time it was a cup week, every training we were doing penalty shoot, penalty kicks at the end. And it was one per player, every player had to go through and if you were missing a penalty, you had to go like to do some runs, like fitness runs. If you score, you just go to the changing room and see ya. And we are doing that um like every session, for every week. Like so, it was like, I don't know, 4 sessions a week, 4 sessions we had penalties and we ended up.. So we were playing the Second league at that time, and we ended up winning two rounds on penalties. And, and we ended up playing semi-final of the [removed for protection] Cup against [removed for protection] and we were a bunch of nobody

Interviewer: Oh wow

Participant: But we beat teams and it was, we were so confident in our penalties so like our goalkeeper would save penalties, we would score penalties and go like, like top corner, whatever, no stress, like everybody was confident. And back in the day when I was doing it, I was just like: Okay, cool. I was negotiating once, I missed my penalty, I love penalties. I missed one and I negotiated with the coach. I was like: Okay, if I score.. So if you missed, you had to try again

Interviewer: Yeah

Participant: But say you missed three times, you have three times the amount of fitness, and if you missed only one, it's only one. So until you score, you had to, to shoot and then whatever happened. And so I negotiated with the coach, I was like: Okay so I missed the first one, if I score, I'm out, and I don't do anything. If I don't score, I do triple whatever amount at the end. And I knew I would score because I don't miss often my penalties, I love penalties, and so whatever, it happened. But my, my, my point is repetition, stress, like people are stressed by penalties because it's the one thing, whatever.. And, and that coach had the perfect idea of making us doing that all the time, and we were very confident and very like um stress-free when we're taking our penalties and I think that's why repetition under those stressful situation make a big difference.

Interviewer: Yeah right. So really training like through repetition, but also just like the stress element of getting exposure to that stress element is important?

Participant: Yeah. Yeah. And especially at a high level.. The Matildas are not used to playing in front of 50,000 people at the moment. That's loud, I could see the players trying to talk to each other. I was super close. And I could see them trying to talk to each other, and they couldn't, they couldn't hear anything, they couldn't like.. So you have definitely the pressure like, them when they scored, when they had the ball, just crossing the midfield, you can hear everybody and it's like, you can't hear, you can't hear your friend, your teammates, but you can hear everything, the pressure, the lights the this, the that.. It's, it's something that they can definitely not reproduce at training, so yeah.

Interviewer: Yeah, absolutely. That's a really good point - just like that whole exposure element. Um would you see any… It sounds like you do see a lot of benefits and like positives of using virtual reality. Do you see any barriers or challenges that may come up?

Participant: Uuh.. I would say some people probably wouldn't feel very comfortable with it. Also like wearing the whole thing, like it's not really like um.. It change aspect of how you would feel in like, because you would wear something like this, in a game you wouldn't. So potentially that could bother you

Interviewer: Yeah

Participant: And so would be um.. Not real, I guess.

Interviewer: Yeah

Participant: Uuhh.. And then um.. I don’t know. Like it's a screen thing also. So potentially screen.. Like, I spend too much time on the screen already so I wouldn't want to spend more time on the screen. But yeah.

Interviewer: Yeah.. So it's just because it's not so real that may be a challenge?

Participant: Yeah, yeah. Yeah… Well, it's more in terms of like we want to reproduce something real and with that, it wouldn't be how you be in a game, you wouldn't have that on your face in the game. So then is it, like um, would you feel as comfortable with your body and running whatever, as in a game?

Interviewer: Yeah

Participant: So that that could bother you, you know what I mean, in, in terms of…

Interviewer: Yeah, I mean, you wouldn't be running around a lot.

Participant: Nah, yeah, yeah, yeah.. But still like…

Interviewer: But still having that thing, absolutely.

Participant: But it would still a bit of, it would still change a bit of things. But you know, that’s all.

Interviewer: Yeah. And do you think um the benefits would outweigh the barriers?

Participant: Oh, yeah. Yeah, yeah. I'm just thinking, not just for myself. I'm thinking in general

Interviewer: Yeah

Participant: Like me, I would, I would definitely like do it and stuff. But yeah, I'm trying to think about other people and what they would think.

Interviewer: Yeah, yeah, yeah. So with you teammates has it ever been discussed to use virtual reality in training?

Speaker: No.

Interviewer: No. Not at all.. What do you think that your teammates, or the girls that you play would think about using it?

Participant: Mm.. In [removed for protection], they would definitely love it. Here uh, some, some players would be interested, yeah.

Interviewer: But do you think some players would be..

Participant: Most of the foreigners…

Interviewer: Do you think some players would be drawn back by it?

Participant: I think it's also, nah, it's not especially that this, I think it's more like people being aware that every tool they can have to help them should be tried.

Interviewer: Yeah

Participant: And sometimes people are like: I don't need that, I'm good enough or whatever.

Interviewer: Yeah, yeah, yeah.

Participant: Or like, what is this extra thing? Oh, it's extra something, it's too much. Me, I'm always keen on whatever.

Interviewer: Yeah, yeah, yeah.

Participant: And I know players, like the more you get older, the more like, you know, you want to improve your like, you’re mature, you want to improve yourself, you're like open to things, it's a bit different so..

Interviewer: Yeah

Participant: Here, it's very young, that's why I'm saying, I'm saying that but.. Players are more and more, like you, you see career opportunities in soccer. So I guess you understand younger that you need to actually work harder and eventually do other stuff to achieve what you want. So that, I think that could be that could work.

Interviewer: Yeah, yeah, yeah. So a time issue as well, do you think?

Participant: It's more like the club. What, what the club can afford, how they can, how they can organise that around training. Because already training is very um time consuming in terms of like when you see your days plus the players have to work.

Interviewer: Yeah

Participant: With the, with the current situation, not many players can afford to.. To do, like for example like in my seasons, I always worked in my life, always, always and, and the three seasons here, two seasons I didn't work, I just worked this season, but two previous seasons, I didn't work and felt so good on the body. Like it's so nice.. Actually didn't work for two months during the season here and same same, the two months I was like: It's good, and I'm happy to do extra stuff. And I would be always the last one at the facility, doing recovery or stuff because I didn't have work after.

Interviewer: Yeah, yeah

Participant: So you can always do extras but when, when you have all the like all the stuff, it's a bit harder. Most players study, work, so can they actually fit that in their schedule is the question

Interviewer: Yeah, right.. So there's an issue with like the money that goes into the sport?

Participant: Yeah. Because currently the, the contracts in A-League are not good enough for the players to, you know, do that only full-time.

Interviewer: Yeah.. It's unfair to like, be asked to work and..

Participant: We are woman. What do we expect?

Interviewer: Yeah, yeah, yeah.

Interviewer: What about your coaching team, um do you think they would be open towards implementing it?

Participant: Um.. Really depends. That’s a good question.. A coach at [removed for protection] would have said no straight away, not even a conversation. Um.. This year, [removed for protection], I don't know. But then like, I don't know, I can't, I can't tell. But…

Interviewer: That’s okay.

Participant: Like I'm also a coach, like I'm coaching also and I'm like.. I would try, like I would try things, and I would actually like um.. Yeah, try it myself and see what the benefits are and stuff before implementing in the team.

Interviewer: Yeah

Participant: To.. Because it's already a lot of mental focus, physical focus during the season

Interviewer: Yeah

Participant: Yes, you want extra tools, but then you don't want to bring stuff that are annoying, or that are not working, or that.. It's time consuming and you waste time and like, you know, so you would want to implement something that is actually working and helping.

Interviewer: Yeah, yeah, yeah.

Participant: And so.. I, I think one of the issues potentially will be to prove that it actually improve skills of the players or whatever

Interviewer: Yeah

Participant: And then, it's like everything. Something is working, ooh I want it. Something is new and it's working. Is it working? Is it good? Is it? You know, it's, it's, it's marketing your stuff.

Interviewer: Yeah, yeah, yeah. So like by presenting research, or like just evidence that it works, they would be more receptive, do you think?

Participant: Yeah

Interviewer: Yeah. Cool. Alright, well, I'm just gonna sum up everything that you have said to make sure I've understood everything correctly.

Participant: Yeah

Interviewer: But overall, you are quite positive towards the use of virtual reality?

Participant: Mhm

Interviewer: and you would be open towards using it if you had access to it. Um and the biggest benefits you see are through repetition, recreating situations um outside of a game that you can't really get exposure to during training, and just like, you know, being able to practise under game situations and pressure situations.

Participant: Mhm…

Interviewer: And the biggest um barrier would be like having a headset, and just spending more time in front of a screen.

Participant: Yeah.

Interviewer: Is that right?

Participant: Yeah.

Interviewer: Cool. And with your teammates, some would be open to it using it, some wouldn't, um and with the coaching staff, it's more like, you know whether it works or not. Is that, is everything correct?

Participant: You're listening very well.

Interviewer: Cool. Do you have any other comments um in regards to the use of virtual reality, or questions, or concerns anything?

Participant: No, no more questions.

**Participant 9:**

Interviewer: Firstly, can you just tell me what you know about virtual reality?

Participant: Yeah, so um.. So like my first encounter with virtual reality was, I think, maybe two years ago, my uncle had this kind of VR, um at his home, and I remember trying it, remember like having the kind of, I don’t know what you call it, but almost the googles on. And um.. So what I like found was that you could enter different spaces and it felts so real, right, which I thought was quite fascinating. Um but to be honest, like since then I haven't really haven’t had any like experience with it. Um, but I do think it's very interesting, and yeah, excited to hear more.

Interviewer: Cool. So thinking about entering different spaces by putting on that headset. Can you think of how it could be used um in football training?

Participant: Yeah. So I think especially for um, in the context of football, you have moments where you just need to react. You need to be intuitive and there's like situations that you cannot really prepare for, right? That's just like, the stuff that you, depend on your training. But I do also think that there's these um set pieces. So for instance, when you do a corner kick, when you do a um penalty, when you do a free kick - those kind of situations, I guess, could perhaps be um.. Using like VR to maybe.. Um.. Promote those feelings under these types of situation where the player who, who performs the kick just need to know that it needs to be in a certain area. Um and particularly thinking about penalty – there is so much like psychological um aspects to the execution of the kick as well that I think would be very interesting to, to yeah, look into if VR could actually be an opportunity and possibility as a player to, to train, um yeah.

Interviewer: That's very good thoughts... Um so it sounds like um the main things that you see it used for are like the set pieces and just getting exposure to different scenarios in football. Is that right?

Participant: Yeah, that's exactly right.

Interviewer: Cool. So can you talk more about how you think it could help improve your performance during a game?

Participant: Yeah so during a game, I think obviously it depends on what level you will be at. I think just looking, now I'm at the World Cup, and I think there is um, these kind of games in the large stadiums, they demands.. They demand different things of you in a sense because there's more spectators, it's, it's a different setting in terms of the media attention too. So I think the VR definitely needs to be able to create that space as well. With the sounds as well and like, you enter the field and you cannot even like hear your own thoughts, right, because it's just like screaming. And I think that's, that's a very like different scenario compared to the scenario with playing at [removed for protection], which I think is, is great too, but it's just like there's only maybe up to 1000 in the stands and you can hear your coach from the sideline, there's many subs um and um... And, and therefore I think the VR needs to be able to really be specific in terms of the context of the player, like is it a player that is semi-professional, professional, like with the national team or whatsoever, um in order to actually have a more like realistic effect. I think that would be my, my take on it.

Interviewer: Yeah. That is really good and that is actually what you can do with virtual reality. So you can simulate like all different scenarios like big stadiums, but also just training fields. Um so you definitely have some really good points there.

Participant: Okay, awesome..

Interviewer: If you had access to virtual reality, would you be open to implementing it into a part of your routine? Of course, it would never replace the training on field but just as an addition.

Participant: Yeah I think like especially being like a player in 2023, like you have been the generation being exposed to lots of technology and just like currently thinking about where um.. So when we're running, we're playing with like kind of a build around our um.. Our body to track like all our distance and sprints and so forth. And I think adding that element into just like, your preparation from a brain perspective almost I think it's very appealing and like, it makes so much sense right, because the brain is so much coordinated into our physical movements and so forth.. So um, and, and the cool thing like, I like, I just texted you as well, there's this like other company who's also doing like this video game whatsoever, where there's maybe, maybe it, maybe that's kind of a thing that is more.. I don't know, but geared towards like situations and movement um, but um regardless of that, I'm very much open as a player to integrating that technology um.. As yeah, as like, but keeping in mind this distinction that, yes, it can be like an add-on, but it will never be a replacement right, and um, and also think, like an add on point to it as well its like, if you're injured as a player, it's a way to keep up with your training, perhaps. Um and I think that is opportunities that hopefully like in the next 10 years, as a player, to see how the sport is going to evolve, develop with also just the general like investment in technology um, and AI too, that's um, something, yeah, I'm, I'm like eager to see where the development is going.

Interviewer: Yeah, absolutely. Again really good points. Um so would you say if you were to implement this, you said it would never replace the physical training, which it absolutely would not... But do you foresee any like challenges um of using virtual reality?

Participant: Yeah, no um, I think that's a very good question. And I think like as a player, you're very much used to like your usual routines in a sense. Like you go on the training ground, you do your like pre-activations to get your body right um, and I think, I think some of the challenges is like this implementing, I guess, transition that needs to be in order for the players to adapt to the technology. I think, and I, and like, that's probably not just like specific virtual reality um challenge, that's like in general when you as a human need to certainly like, or start adapting to new things. So I think that's probably one, and maybe like specifically, it would be to know when the VR probably could have the best effects in a sense. Like should you do it in the morning when you have a training in the afternoon, or should you do it just prior to training, is there like any limits on how much you can do it? Like all these sorts of information as a player because also this level, like you're very much sensitive to what you do, and what you don't do, like what I just wrote you - let's see if we can like touch base later, right, because I need my sleep, that's, that's kind of how it is. So in terms of that, there's different like barriers I think in the implementing like process. But I do think that it is something that is going to be a thing that more players than just me would be interested in at least like trying.

Interviewer: Yeah, absolutely. Nah, that is really good points. And again, it is super hard when you haven't had any experience with it, like you don't really know what the barriers would be before you have experienced it, but really good points.. Um so coming back, you said that for the World Cup it would have been good preparation to sort of get exposure to being in these big stadiums with crowds and stuff.. Um and then you also touched upon like set pieces and just like the moments within a game. So for you as a midfielder, and as a player at a very high level… How do you think the biggest benefits would be to using virtual reality?

Participant: I definitely think like.. So taking like my leg of exposure into account, just from like my initial thoughts would be that it would have such a like mental relaxation kind of aspect to it in the sense, because you know that you've kind of been in this situation before, it's like repetition. Like when you do something you feel more comfortable doing it again, it's like when you train a penalty as well. Yes, you can really train like your structure, how you go to the ball, how you walk towards the ball, like, you know, there is this like time and space where you're like, um but... So, so I guess like, it's more so like the psychological benefits I think that would be interesting for me, especially now I'm a player that is like one of the younger players in the team and some of these new um situ, these situations are completely new for me right. So already have like got some of real-life situation training with it, I think that would be a very cool um benefit and not something I really can see like a challenge to..

Interviewer: Yeah, absolutely..

Participant: Was that like a full like, was that like an all-around answer?

Interviewer: Yeah, that makes really good sense. Um and I think that is how many people and many players that I have spoken to have said that like sort of mental preparation and just gaining exposure before being in the moment.

Participant: Cool…

Interviewer: So that's really good... Has it ever been discussed to use virtual reality with your teammates, either at [removed for protection] um or at the National team?

Participant: No unfortunately not, actually. Like it's, it’s something that I think um.. I don't think people think about it like as a possibility to be quite honest, just because you don't as like.. I guess as a player, especially professional players, very much like into their own space in a sense. It's like, you do your own routines, you know what works, you test different things, but it's something that you try to do that's all within your control in a sense. Where when you get new technology, it's like, it's the coaching staff. Or like something within the staff that's suddenly presented to you and you're just like: Okay let's adapt, let's try it on. You don't really have a choice. So um.. So I think this should also be something that needs to be kind of coach um initiated. Or like I don't, I don't know who actually would have that like.. I guess the tracking team, it depends obviously of the professional like um resources there are within those certain teams, but um.. Yeah, I don't think, I think players like they needs to be, be like presented to it in a in a very like positive way in order to also like.. Have that curiosity to, to try it.

Interviewer: Yeah, absolutely. Um so um the players that you play with at [removed for protection] and at the National team - do you think they would be open towards using it, or do you think they would need sort of that encouragement from the coaching team?

Participant: Mhm, I think my [removed for protection] team would be more curious towards it than my [removed for protection] teammates. I think my [removed for protection] teammates, I think they're like exposed to so many different types of technology in their own like professional clubs, so um.. It would, I think they would need like more encouragement, but still like.. And then like that encouragement should probably be like many days before game day too. Um but like recently, we just kind of implemented new technology to the team for this World Cup. We've been doing Trackman, I don't know if you know it, but it's like technology, Danish-developed, and it's essentially like a simulator too that can track set pieces, specifically

Interviewer: Oh wow

Participant: And like show to you as a player if you hit the ball, how you hit the ball, if the ball would like have gone in so they kind of know the movements of the goalkeeper, for instance on a penalty too. So what we do is put this trackman behind, behind the goal, and then you as a player like shoot and then on the screen, on the iPad, our coach would be like: Yeah, that would have gone in on like a high professional level. Um which would actually be cool, like imagine if like Trackman and this project could be kind of merged because they have so many simulations um about all sorts of different, both in like the Women's team, on the Men's team on the professional level, and like lower, lower level. I tried the technology like many years ago, when it kind of started to um develop and it's originally inspired from golf, like when you do your drive for instance it's like, it can come up with different, I guess, almost like recommendations.. If you have a proper coach, who knows how you hit the ball and like what you need to do differently. Um so like definietly something maybe to look into and something to maybe like reach out as well.

Interviewer: Absolutely. That sounds really cool. Um and it really just shows how many different technologies there are, like Trackman and then virtual reality, and then also the brain technology company you sent me, um and there would be so many more. It's very interesting.

Participant: Yeah. Yeah.

Interviewer: And so within your coaching team - do you think they would be open towards implementing virtual reality?

Participant: Yeah I think like, I can't really see like a reason why they wouldn't in a sense. Um I still think they're at the point where they are only integrating technology if it's been tested for like a good period of time and so forth. Um just because of the nature of the team, um however, I do think like if you go to certain like club teams it would probably be more like, the barrier I guess would be probably lower. That's just like, I don't know if I'm biassed, but that would be my like initial thoughts about that.

Interviewer: Yeah, that makes sense. And that's really good insight. Um alright, so I will just sum up everything that you have touched upon. Um so overall it does sound like you are quite positive towards virtual reality um as an addition to training, not um, you know, it would never replace anything. Is that right?

Participant: Yes

Interviewer: Cool. And the major benefit you see are like exposure um and then mental preparation for, you know, situations you may encounter in games. And then also if you're injured you can sort of keep the mental training going…

Participant: Correct.

Interviewer: Cool. And the um barriers you see is like, you know, just getting used to being in a virtual environment and getting used to using it so you wouldn't implement it like a week before an important game or something. Is that right?

Participant: Yeah, that is right.

Interviewer: Cool. Perfect Do you have any additional thoughts or comments and or questions in regards to the use of virtual reality?

Participant: Um.. No I don’t think so

**Participant 10:**

Interviewer: Can you start off just telling me a bit about what you know about virtual reality?

Participant: Umm probably not a whole lot. Umm, oops sorry, my laptop has just had a moment.

Interviewer: That's okay.

Participant: Um yeah probably not a whole lot. Um, I just sort of, I guess, I just think of like, I don't know, things wired to my brain and like, I don't know. Training, like obviously not physically, um but in another, I don't know, conscience or whatever, but um, yeah, I actually don't know a tonne about it, to be honest.

Interviewer: That's okay... So essentially you can put on this headset and then you can get immersed into this virtual environment.

Participant: Yeah

Interviewer: Um, and then, um yeah, you can get immersed into this headset and then you can be put into a different environment. So for example, you could be training at home, um but then like, by putting the headset on you could be immersed into.. The football field or whatever.

Participant: Mhm…

Interviewer: So can you think about how it could be used in football training?

Participant: Um.. I guess.. I mean, you could probably program it to be like different situations based on like what you'd wanna work on. Um, so yeah, it could be like a penalty kick, it could be, like if you're a goalkeeper it could be a goal kick, focusing on that, it could be, I don't know, going up for a header, or being a player one V one. Yeah, I think like isolated like situations would be good based on like, yeah, your position and things you'd wanna work on.

Interviewer: Yeah, absolutely. Um so can you talk about how you think that could help you improve your performance in football?

Participant: Um.. Well, I guess like, I've tried visualisation, like visual, visualisation... Yeah, it's a word. I've tried that before but I'm, honestly like, sometimes when I shut my eyes like it just goes blank. Like I'm not very good at it.

Interviewer: Yeah

Participant: But I guess, it probably is a bit like that, and it's probably like, yeah, I probably wouldn't have to visualise too much cause it’s already in front of you. But I guess you're training your brain to know what to do. So I guess your body can just follow suit when you actually have to perform it, like on the field.

Interviewer: Yeah, absolutely. So a lot of like mental preparation and exposure to future situations, would you say?

Participant: Yeah. Yeah, definitely.

Interviewer: And can you talk about how that could help improve your performance during a game?

Participant: Um.. Well, I guess like.. What I've found, especially since I was a young player to like a bit more experience and stuff, the more games and situations I put myself in, the better I'm equipped to performing under pressure. So I guess if you're using that virtual reality, you've seen those situations before, you sort of don't have to think, and there's probably less pressure and you kind of know what to do and to perform properly, I guess, yeah.

Interviewer: Yeah, nah that makes sense. Um and that is really what you can do with virtual reality, like that exposure to situations that can't really be recreated during training..

Participant: Yeah, 100%, yeah.

Interviewer: So um, if you had access to using virtual reality, of course it would never replace the physical part of training, but as an addition. Would you be open to using it?

Participant: Oh yeah, definitely. I feel like it's one of those 1%’s that like, I guess, you know, it would, maybe it'd be better for some people and some people don't like it. Like it's, I guess it's a preference, but I mean I'd be definitely open to, yeah, 100% if I had access to it.

Interviewer: Yeah, cool. And if you had access to it, so you were talking about like visualisation and exposure and stuff.. Would you need the virtual reality to have any sort of like capabilities or like any specific situations it would need to be able to expose you to if that makes sense?

Participant: Um.. Yeah, I guess, well I'm a midfielder, so I guess there's certain stuff that comes with that, that I wanna work on that I'm probably wanna get better at, so.. Um, yeah, I guess so.

Interviewer: Can you talk about and some of the things that um you would need as a midfielder?

Participant: Yeah, I gu, I guess like.. You know, turning, you know, 360 degrees, like you know, cause pressure can come from any angle. So I guess, you know, those sorts of situations where you have to turn and play a pass quickly, or, I guess you're passing range as well.. And a lot of it with midfield can be decision-making, like when to play a pass ball, when to keep a ball, when to know when there's pressure. So I guess, yeah, those are sorts of situations I'd look at.

Interviewer: Yeah. So a lot of about improving your decision-making would you say?

Participant: Yeah, definitely.

Interviewer: Absolutely. Um so we've spoken a lot about the benefits and like the potential use of virtual reality. Would you see any barriers with implementing this into your training?

Participant: Umm.. I mean for me I know it's like a sort of supplement way of training because obviously you can't really beat the physical part of training. But I guess for some people there could be a bit too much focus on it and I guess that could take away from other parts of training. If people think that like: Yeah, I'll just do this and I'll get really good. I guess that's not really how it works. It's sort of, sort of backs up what you do um on the field as well. So I think, yeah, if too much focus was put on it, um it could, you know, not improve you as much.

Interviewer: Yeah, absolutely. So just finding the right balance between the two?

Participant: Yeah.. Of course

Interviewer: Cool. So um, what about um, like among your team members - has it ever been discussed to use any kind of virtual reality?

Participant: Umm.. Actually no, I’ve never really had a conversation with my teammates. I've...The only thing I've sort of seen was um.. I'm a Liverpool fan and um, there's a player for Liverpool, he plays right-back but a bit in the midfield too and he's like a really good free kick taker and before the last Champions League Final we played, I think it was Champions League. He was practising free kicks with like this headgear on and I was like: That’s pretty cool.. So that's probably like the only time I've really.. I mean, I haven't talked really much with to my teammates about it, but I've seen sort of, actual, sort of that sort of thing being used.

Interviewer: Yeah, that's really cool.

Participant: Yeah...

Interviewer: What do you think the attitudes among your team members would be towards the use - do you think they would be open to using it?

Participant: Um.. Yeah, I reckon so. I guess.. It's worth, like a lot of my teammates would probably be worth, like trying it and see how they like it. Um, but yeah, yeah, I think it would be, yeah, good.

Interviewer: Cool. So I guess it hasn't been discussed with your coaching team either… But um how do you think they would think about the use of virtual reality?

Participant: Umm.. I don't know, it's funny, I find some coaches are super old school these days as well. Um so some of them might think not much of it. Um but I think there would be some coaches that, that would be open to it as well. But yeah, I'd say there's like a few coaches that just think, you know, their way is the only way, so that's sort of what some coaches are like.

Interviewer: Yeah right

Participant: I think players would be probably more open to it than coaches to be honest but.

Interviewer: Yeah, right. Nah, that's cool. Alrighty, so just to go back to talking about - you know, the use and the application… So you said that you have tried to do some visualisation and that virtual reality may be used um in, like instead of this… Can you talk more about how that could help you on field and your performance?

Participant: Um.. Wait sorry, could you repeat the question again. Sorry, my phone was like pinging the whole time…

Interviewer: Ooohh yeah that’s okay, no stress. So you said that it may be used to like expose yourself to situations and to sort of, like instead of visualisation…

Participant: Yeah

Interviewer: Can you talk more about how that could help you um on field and improve your performance?

Participant: Yeah... Well, I guess, like we said, like expose.. Like I think it is hard sometimes to recreate the same situation over and over again cause in training, especially, in, mainly team trainings, there's, you know, you're working a lot of different things on different people. So it's not as specialised. So I guess, if you were repeating the same sort of situations that, you know, is more um fitted to what you might face in the game, um yeah, you just get better at those situations and decision-making and pulling under pressure and stuff. So I think it's just like a really good individualised sort of tool that you can use. Cause I guess a lot of training we do is very broad because like, there's a whole, you know, team working on it, I guess.

Interviewer: Yeah. Absolutely. Um that’s some really good points. And that's another, like you mentioned another good benefit of, like individualising the training, rather than training as a group because you can tailor it to you specifically…

Participant: Exactly..

Interviewer: You also mentioned decision-making... Can you talk more about how that could be improved through the use of virtual reality?

Participant: Yeah. I think.. When, when I've made a mistake in a game, you know, you go back and look at it and then, you know, you wanna fix it next time. But I guess, you never know when you're going to be faced to that situation again. It could be, I, you know, chose the wrong pass, or like I overhit a pass that was supposed to be to someone’s feet. I guess um, the virtual reality, I could pick when I was gonna work on that, and then, you know, get better at it through that. Um, but yeah, I think like, yeah. Does that make sense?

Interviewer: Yeah, yeah, yeah…

Participant: Sometimes I waffle words and I just start talking…

Interviewer No, no, it makes perfect sense. And like, you know, you can sort of go through the situations, but from a first-hand perspective um rather than “just” looking at videos - not that there's anything wrong with that….

Participant: Yeah... And I guess you could like, pretty much recreate, I don't know how good the like technology could be, but you could recreate that exact sort of moment um that you faced in the games because you, I mean, you don't really get re-do, like a re-do when you make a mistake, but I guess this is a tool that you could actually recreate like a, the same situation and work on that, and then when you're put in that situation again, you know, you're probably putting yourself, you know, in a good position to make a better decision.

Interviewer: Absolutely. Nah, that's excellent points… How about, you said that that player from Liverpool was practising free kicks, and you also mentioned um that you could practise heading… Can you talk about how that could help improve your performance?

Participant: Yeah, I think um like from a deadball, like a free kick, or like, yeah, like a goal kick or a corner even, I think they're really specific techniques that you can like do over and over and over again to get the right um… I guess the right technique and right um spot. So I think like those sort of situations would probably be the easiest to work on cause they're very like standards set-up, you know, kind of know what you're getting out of them. Um and obviously, they're big moments in games that can literally win or lose you a game. So I guess they're really important things that would be, yeah worked on that. And probably, yeah, like the easiest to set up, I guess. yeah, absolutely, yeah.

Interviewer: Yeah absolutely. So out of all these benefits so like decision-making, getting exposed to pressure situations and like set pieces - free kicks, corners, penalties. What do you think would be of the greatest benefit for you to use as a midfielder?

Participant: Um.. I guess like.. I mean, for me personally um, I've always struggled with like long passes, um especially under pressure. Like, you know, when you're really relaxed you can do a lot, but when you're actually, yeah, when there's, you know, pressure to perform something um, I guess sometimes your, everything goes out the window um and you can make more mistakes. So I guess that's sort of like long passing um sort of, through ball stuff. I guess, from a dead ball perspective, like obviously like penalties would be important for, I guess, any position. Um.. And corners as well, probably for a midfielder more so. But yeah, I think from a midfielder point of view there would be a lot of, more in depth situations, I guess, because there's so much that you know comes with being a midfielder. Cause I guess with, with defenders, like centre-back, you could always work on one V one defending and stuff for goalkeepers. But, and strikers one V one attacking, or finishes, like shooting and stuff, but I think there's a lot of like, intricate sort of situations that midfielders faced with. So I guess, yeah, those, it would be mainly around decision-making, I'd say.

Interviewer: Decision-making… Yeah.

Participant: Yeah.

Interviewer: Can you talk about some of the other moments or situation as a midfielder that it could be useful for?

Participant: Um.. I guess your positioning.. Um I think like working on, you know, how close you have to be to certain players or further away, I guess maybe coaches could use it as a tool to, instead of just showing your video where you need to be you actually can see where you need to be, I guess. So yeah, from a positional point of view it could be really helpful, I think.

Interviewer: Absolutely. Cool, that are some really good points of how it could be used and really good insight into how you see it could be used.. So talking about all of this, um you mentioned that the only barrier you foresaw was like if some players may struggle with like finding the balance between them... Have you thought about anything else as we, as we've spoken about the use of virtual reality that could come in as a barrier, or you know, might take you away from wanting to use it?

Participant: Um.. I mean the only thing that really comes to mind, I guess, could be, if.. You wanna use it that, you wanna use it to benefit you, so I guess like, if you are putting yourself in unrealistic situations, or, I guess, it's the way you use it as well. Um, you'd want to be using it, you know, that's fitted to your sort of position, or what you wanna work on. So I guess, it would be important, I think, to have some guidance of what - you’d need to know what you wanna work on and like that sort of thing. Otherwise, you don't want to use it just for the sake of using it, I guess. You probably wanna get a lot of it out of it so I guess that could be a barrier if you actually don't really know what you wanna do with it.

Interviewer: Yeah

Participant: So it can probably be, yeah, you're just using it for the sake of using it, I guess, that's - you're not really getting much out of it. So I guess I could see that as a barrier as well.

Interviewer: Yeah.. So just some more guidance of what you need to work on, you think?

Participant: Yeah, of course.

Interviewer: Cool. Um and if you got access to a headset, and you said you would be open towards implementing it. Do you think it would be like easy to implement into your current training routines?

Participant: Um, yeah, if I had access to it, I guess I'd probably want to know like, yeah, like how I would use it and, you know, know all those specifics. But yeah, I think it, I mean if it's explained and pretty straightforward, I think it'd be pretty easy, yeah.

Interviewer: Yeah, cool. Perfect. Alright, I'm just gonna sum up everything that we have spoken about to make sure I've gotten all right information.

Participant: Okay.

Interviewer: So overall it sounds like you are quite positive towards um the use and potential implementation of virtual reality in your training. Um and you see many benefits such as exposure to pressure situation, training specific set pieces, um decision-making and positioning... Um and the only barriers are like the lack of guidance and also like the balance between the physical training versus the virtual training… Um and it hasn't been discussed among your teammates or your coaches, um but you think your teammates mostly would be positive to the use um and coaches depends on what kind of view on training they have… Is that correct?

Participant: Yeah, definitely.

Interviewer: Cool. Perfect. Do you have any other, before we move on to the second part of the interview, do you have any other like thoughts or comments or questions in regards to virtual reality?

Participant: Um I guess one thing I thought of was like.. You know it could reduce injuries… Cause I guess with, you know, athletes wanting to train as much as possible, we can tend to exert ourselves a bit too much.. Um so I guess if this was introduced then it would be a way of training without pushing your body too far.. So yeah, I guess it could reduce injuries as well.

Interviewer: Absolutely. Um cause you can still get that like mental kind of training.

Participant: Yeah, of course. And you're not, yeah, pushing your body too far, I guess.

Interviewer: Yeah… And do you think that if you were injured - do you think that could help you like maintain your head in the game kind of?

Participant: Actually, yeah.. I just, yeah, that's actually a really good point, I think so, yeah. And also I guess.. Yeah, it's a way of you still improving, even when you're injured cause, you know, some people are out… You do an ACL, you're out for a year, like, that's a long time away from it. So I guess it could improve, or like keep your, I guess, mental sharpness, um you know, really good. So maybe when you come back you’re not as like behind, I guess, cause I guess when you see players coming back from injuries they're always like a little bit a step behind you can notice because they haven't played for a while. So yeah, yeah, definitely.

Interviewer: Yeah, absolutely. Nah, that's very good insight. Um was there anything else in regards to virtual reality you thought of?

Participant: Um.. Have you seen Black Mirror? Have you seen that TV show?

Interviewer: No, I haven't.

Participant: It's make me think of that, it's just like random, like, there's just like, some horror stuff on it where it's like someone tries a virtual reality and then it like has a brain of its own and then takes over your mind and stuff. So I'm just, I'm thinking of that right now. But apart from that no…

Interviewer: Cool.. Um, alright, well that was really good insight. Thank you so much.

Participant: No worries.

**Participant 11:**

Interviewer: To start of with can you just tell me what you know about virtual reality?

Participant: Ummm I think the best way to describe it is that it's using alternative means to perform something we can't physically do in person.

Interviewer: Yeah.

Participant: So it can be used, you know, obviously people play for video games a lot of times or high stressful situations - that sort of thing. So it's a good tool for just extending our mental capacity.

Interviewer: Yeah, yeah, very good. So do you, have you had any experience with using it at all?

Participant: Just like little video games - that type of thing. Nothing too crazy, you know.

Interviewer: Yeah, right. So you've never used it in - for football training or anything?

Participant: No

Interviewer: No... What are your thoughts about using it in football - how do you think it could benefit your performance in football?

Participant: I think it would help out heaps, especially - I already visualise on my own.

Interviewer: Yeah.

Participant: But I think that actually get a clear picture in front of my mind and my eyesight. It would just help tenfold, you know, especially cause... You can already picture so much, but when you have other outcomes coming through that you are unpredictable, you know, it makes you have those quick seconds reactions to be able to do it out on the actual pitch.

Interviewer: Yeah. So sounds like it's a lot about that exposure element

Participant: Yep…

Interviewer: ..and getting exposure. Can you talk about some of the specific skills that you feel like you could improve using virtual reality training?

Participant: I think for me a lot of it would be my decision making. Even when to shoot, when to pass, umm being able to see certain passing lanes open up last minute and even reading the game just overall.

Interviewer: Reading the game overall… Yeah. Cool. So what about your performance on field and during a game? How do you think virtual reality could help improve that?

Participant: I think it would just exponentially make me a better player. I've always found actually playing Call of Duty - the more I played that actually growing up, it actually translated more to the field. Just because I would have to have that quick decision making

Interviewer: Yeah right

Participant: So already know how important like the video games are

Interviewer: Yeah, yeah, yeah.

Participant: Yeah, so I know that it, it definetly helps out.

Interviewer: Yeah, right. So just really - firstly get you exposure to situations you may encounter during a game.

Participant: Yeah.

Interviewer: But then also like, you know, quicker decision making through exposure. Is that right?

Participant: Mhhm. Yeah.

Interviewer: Yeah cool. So if you had access to using virtual reality - of course, it would never replace the physical training, but as an addition - would you be open to using it?

Participant: Ohh yeah massively. I think it needs to be implemented more in training so…

Interviewer: Yeah… So has it ever been discussed to use virtual reality - you know, among your teammates at all?

Participant: Not with my team - I don't know... I don't know any team really, actually in, in [removed for protection] that uses it.

Interviewer: Yeah

Participant: I don't know how much it has gotten exposure as well.

Interviewer: Yeah

Participant: It’s still probably quite new in Australia, but I think our team would benefit from something like that.

Interviewer: Yeah right…And what about with your coaching team - has it ever been discussed among them?

Participant: No it has not…

Interviewer: Nah, na.… Do you think that your team, you know, if they got access to it - do you think that they would be open to using it?

Participant: Ohh yeah, I think they definitely would. A lot of the girls are still quite young so I think it was just help out with their soccer IQ as well.

Interviewer: Yeah - yeah right. So it sounds like you are quite positive towards, you know, the use and the implementation of virtual reality.

Participant: Yep.

Interviewer: Would you see any barriers to what using it?

Participant: I think starting off - obviously the financial means would probably be the biggest issue for a lot of teams. And that type of stuff to run the programmes, get multiple players using it on a consistent basis. And even just the programme as well - that whole development. I think.. The first couple of years is always going to be hard for something like that, but then the more, you know, you kind of will deal with that things and everything - I think will become a lot easier and then a lot of people will start to use it even more so.

Interviewer: Yeah absolutely.

Participant: So I don't see any major barriers - that I can think of - like I said, more of that funding, resource type stuff.

Interviewer: Yeah, yeah, yeah. So it's like having access to it is a bit hard?

Participant: Yeah.

Interviewer: Yeah, absolutely.

Participant: And even just finding the time

Interviewer: The time, yeah…

Participant: You know, depending on… Let's say the different levels of leagues - whether it's more community, if it's like semi-professional, pro - that type of thing, how much time is actually allocated per week that someone can do as a team, or just like collectively that type of stuff.

Interviewer: Yeah absolutely… Cool. So just coming back - you said like - it could help a lot with decision-making, but also like reading the game. Can you talk more about how it could help you better read the game during a match?

Participant: I think... It's one of those things where the more you have repetition of something - it becomes muscle memory. So if I'm constantly seeing a certain picture come and play on my mind - I become more comfortable at making that decision - where it becomes a lot quicker.

Interviewer: Yeah.

Participant: And so if it's - let's say, consistently... Holding on to the ball too long and not seeing that pass, passing lane open up.

Interviewer: Yeah..

Participant: The more I see that happening I can be able to read subtle body languages of the opposition, my teammates, that sort of thing… So then it becomes a lot quicker.

Interviewer: Yeah. Cool. So through repetition and exposure?

Participant: Yeah - the repetition, the muscle memory is massive.

Interviewer: Yeah, yeah right...

Participant: Yeah… Yeah, it's the... It's like the ten thousand hour rule. That's where it comes down to.

Interviewer: Yeah right

Participant: You know to really master your craft.

Interviewer: Yeah, yeah right. What about that exposure - would there be any situations that you feel like would be very beneficial to get exposure to compared to others?

Participant: Uuummm.. Definitely probably like you said that the penalty shots.. I think that's good - a lot of people can't handle that pressure when it comes to it and they don't want to be the one taking it.

Interviewer: Yeah.

Participant: I think for me as being more of attacking centre mid it's more even just.. Finding time to shoot - I think if I got more of that repetition of being outside the box - of just understanding the dimensions of the field, that - and just, just shooting. You know, just seeing that awareness a lot more and trusting myself and... Using the virtual reality to help with that.

Interviewer: Yeah, cool. So do you feel like that training in a virtual environment could help you gain like a greater confidence in your performance?

Participant: Ohh yeah. Exactly. I think because… At training out in the fields, you know - we take so many shots and sometimes becomes very frustrating.

Interviewer: Yeah.

Participant: And everything - so kind of - you beat yourself up.

Interviewer: Yeah.

Participant: And so I think if you keep continuously doing that. In that manner - through the virtual reality is that…. It wouldn't hold as much context every time you miss as it does when you actually do it in person. But I think at the same time it's, it's like a little paradigm where it's still - you're still going to get more... More confidence from missing though too. It's not as heavily weighted in a way.

Interviewer: Yeah, right.

Participant: So it's like a... What’s a good word? Kind of like a feel for it type scene, but there's not as many, I guess, consequences in a way.

Interviewer: Yeah that makes sense. Yeah, that's really good. And so you mentioned in the beginning that you do a lot of visualisation. How do you think virtual reality could be used as a mean to carry out visualisation?

Participant: I think just more so it's.. You're getting more of the.. The surroundings, the feelings across the board.

Interviewer: Yeah.

Participant: Like I said, it's… I personally have a very active imagination in, in everything and I know some people don't have that. And so they can't be as creative - that sort of thing. Where it’s like - I've watched the game enough to understand, you know, how defenders move - you know all that type of stuff.. Whereas people who maybe aren’t at that level - it's able for them to fully be in those situations and not, let's say - have those consequences as well… So…

Interviewer: Yeah… That's really good. And that makes sense. So if you were to have access to virtual reality - what would be the biggest thing that you would use it for? So we've talked about like exposure, decision-making, reading the game and stuff like that through exposure.

Participant: Yep.

Interviwer: And repetition.. What would be the greatest benefit for you do you think?

Participant: I think for me it would probably actually boost my confidence.

Interviewer: Yeah..

Participant: More than anything – just… Being able to back myself and, and have that quicker decision-making. Where it's only, you know, one or two touch - that sort of thing where it's.. You know, play and go - a little - like they call like Tiki-taka football in Spain and everything - that type of movement. And I think that's all like the plan. Being able to back myself using the virtual reality as well and see it's happening - would just increase my confidence even more so…

Interviewer: Yeah absolutely… Cool. Alright, so I'm just going to sum up everything that we've spoken about and correct me if I'm wrong or if there's anything you want to add, but overall it sounds like you are very open and positive towards the use of virtual reality?

Participant: Correct.

Interviewer: Cool. And the biggest use you see - it is just to get like exposure and repetition and like improve your decision-making, to be able to make quicker decisions and also to read your game and essentially - like in the end because you get repetition it could improve your confidence.

Participant: Yep correct.

Interviewer: Perfect… And the biggest barriers or drawback you see are really just the accessibility and affordability aspect.

Participant: Yeah…

Interviewer: Yup… And that could be overcome by, you know, just implementing it slowly over the years?

Participant: Yeah, 100%. Just like I said in that funding and you know and clubs backing and seeing, you know, the scientific research and, and all that come through and show that it's a good tool to, to be used. You know, I think it's gonna be... Helping heaps of teams, you know, just progress football across the world and all that - and a lot more teams probably will start to implement stuff like that when they do get their hands on it, so…

Interviewer: Yeah absolutely… It’s just about making it more accessible.

Participant: Yeah, exactly.

Interviewer: You know, it’s slowly becoming more affordable but there’s still a lack of funding and stuff..

Participant: Yeah…

Interviewer: Yeah cool. Perfect. And then - it hasn't been discussed with your teammates and your coaching team, but overall you do think that they would be positive to the use?

Participant: Oh yeah, I know a lot of them would love to do something like that.

Interviewer: Yeah.

Participant: To even get in and even do some trial stuff - that type of thing. I think my team would definitely be all for that.

Interviewer: Ohh cool. Perfect. That’s good to know… Alright, so before we sum up this part of the interview.. Do you have any other like thoughts or questions, or anything you want to add about the use of virtual reality?

Participant: Ummm I think it definitely is like a great tool. Like I said, I've played video games, you know? So I understand how much that has helped me, personally, especially with that decision-making. You know it's... It's sometimes just those seconds, you know, that make all the difference. That's what it is. So if you already have that's in the back of your mind, you know, you’ve played it thousands times through the virtual reality. Why not – it becomes easy… It's like the back of your hand, you know it. So it's definitely a good tool, I think it should be used more.

Interviewer: Yeah, absolutely. And hopefully it can be implemented more over the next few years..

Participant: Yeah, that'd be nice.

Interviewer: Cool. Well that's really good that you are super positive towards it…

Participant:: Yeah, definitely..

**Participant 12:**

Interviewer: Can you just tell me what you know about virtual reality?

Participant: I know a few things, or like a lot of people, or I've seen, like you put on the goggles and it's like… A reality, but it's not your real reality, like it's a made reality in front of your eyes. So it's kind of like being in this world, but it has no limits, no boundaries, kind of - and you can do it for a lot of things.

Interviewer: Yeah.

Participant: Yeah, use it for, yeah, a lot of different things. I've seen obviously different games and stuff where it's like crazy fantasy, but you can also make it very realistic. I've seen people like talk to their dead daughters and sons like through virtual, AI reality and stuff, so it’s just… It’s a crazy new world, I would say, like technology building reality.

Interviewer: Yeah, absolutely. Have you had any experience with it yourself?

Participant: No. I actually have never, like I've only seen other people do it, or seen videos - I have never had the goggles on myself, I'm pretty sure.

Interviewer: Yeah, right.

Participant: I think I would be remember if I had, yeah…

Interviewer: Yeah for sure. Can you think about how it could be used for football training?

Participant: Mmmhh.. Your project, like the penalty, I think it's a really good idea with the pressure.. And just like that specific situation freaks a lot of people out. I think it could be used in a lot of like 1V1 situations as well. You could make it kind of like - I don't know if you could call it mirroring, but when you have a person in front of you, like you could probably probe.. I don't know how you technology-wise were going to do it but like have a person in front of you - like if they go to your right, you go to the left, like kind of the motion idea - do you get what I mean?

Interviewer: Yeah absolutely

Participant: Like kind of - you have one person coming up and depending on how they move you, you need to make a decision on how to move.

Interviewer: Yeah, yeah, yeah.

Participant: Yeah - I think that could be a pretty good idea because that's a lot of the situation, I would say, freak people out like in games, if you're like: Okay, there's a person standing here. How do I, like, get around them most effectively?

Interviewer: Yeah

Participant: I would say that's a pretty good one... Hmmm…. 1V1... I don't know if you can use it for like shooting in general - I don't know how it works, but like shooting targets, like penalties, like one situation, but also free kicks and corners - like more specific things

Interviewer: Yeah, absolutely.

Participant: Just like target… Yeah, just like shooting in target. Drills as well would say is pretty good – that’s always something you can be better at. And yeah, again, I don't know what the boundaries are, but like it would be smart if you could go all the way down to the details and be like tactical. I don't know if you can see the whole team in front of you or something in virtual reality, but really just be like: Okay we're working on these patterns - if she goes there, you need to go here - like kind of make everyone see what their position is supposed to do.

Interviewer: Yeah, absolutely. Like you can do so many things with virtual reality…

Participant: Yeah

Interviewer: …it's hard when you don't have experience.

Participant: Yeah

Interviewer: You know like you can practise like 1V1, you can practise like shooting, different drills, like heading drills which is really good.

Participant: Yeah, yeah.

Interviewer: And then you know - having players in front of you or like one to left, one to right – like where do we go and stuff?

Participant: Yeah - even technique drills would be nice as well, like juggling and stuff to keep the ball close, I would say.

Interviewer: Yeah

Participant: Yeah - those are the ones on top of my head, yeah.

Interviewer: Yeah. Cool. So how do you think this could help you become a better player during a game?

Participant: I would say like obviously we do a lot of different things in practise

Interviewer: Yeah

Participant: But I would say this could add like another aspect of like, like in reality different - like even though you're doing a drill, it can turn out to be different in reality because of the grass you're playing on, because the ball is jumping, because the coach wants you to do different stuff, whatever, but I feel like this can be more like specific and probably help you concentrate in one area and really just be focused there.

Interviewer: Yeah.

Participant: And just doing things over and over again - I would say like the penalty is a good example, but like you can it's not that often that you do it in a game because all games don't go to penalty and maybe you're not the first shooter - I don't know, but.

Interviewer:

Participant: It's just you can do it all over again and train for this situation so when you're actually end up in that situation in real life, you're like: Fuck, I've been here before. What did I do in - like what worked in the program?

Interviewer: Yeah… So sort of through repetition you think that could help you improve?

Participant: Yeah, a lot. A lot, a lot. And maybe even, yeah, trying new situations - like maybe you have a team that doesn't play a lot of long balls, or a lot of high balls or whatever. But you could train that to still like keep that fresh in your mind. So yeah.

Interviewer: Yeah, absolutely. That's really good. So mainly you would say like through like practising drills and like shooting the ball. So like your skill in general and then like through exposure to different situations and then through reputation and just like tailored like training. So like you can practise the same thing over and over.

Participant: Yeah. Mhhhmm. Yeah. Yeah. Yeah. Yeah, exactly, yeah.

Interviewer: Cool. So if you had access to virtual reality, would you implement it? Of course, it would never replace the physical, but just as an addition – would you be open to implementing it?

Participant: Mmmhm, definitely. Why not? Like obviously there's not a lot of research and the program isn't ready yet.

Interviewer: Yeah.

Participant: But I would say like soccer is so - or football is so technical right now anyways, like with our GPS's, like blah blah blah - pulls up how many sprints do you do in the training? So it's like - and things like that have shown to like help people actually push them, push themselves and stuff so it's like - why wouldn't you try some - like I don't know if I would be a big fan of it because I haven't tried it, but I would definitely do it. Like why wouldn't you take an option that's there in front of you and really give it a go? Like it sounds interesting so far. So… Definietly.

Interviewer: Yeah. Yeah cool. So sounds like you're quite positive towards it.

Participant: Mmhmm. Yeah, definitely

Interviewer: Cool. So what do you think would be the greatest benefit of using this technology? So you said a few things it could help with, but the greatest…

Participant: Like if I have to choose one?

Interviewer: Or just a few – like how do you think it could help you the most?

Participant: Yeah. I would definitely say get used to… Like how you react in specific situations. So like my brain keeps going back to like penalty obviously, but also the 1V1 - like depending on what type of player you have in front of you and what they're doing - how you're going to react. So I feel like their reaction and the confidence inside yourself is definitely two of the things where it could help a lot, I would say - confidence wise and just like believing it - I know like it's not real life, it's still a program but like, if you - if it works here, hopefully it works in real life as well. And yeah, just give it a go - I would say confidence a lot and just getting used to… Yeah - the things you don't necessarily train every day.

Interviewer: Yeah, yeah, yeah. So can you talk about how it could help your confidence?

Participant: Mmmhmm – yeah. I think a lot of players - like I would say I'm not the most confident player, I'm not not-confident either, I'm kind of like in the middle - you have good games, you have better games. But I would say if you can train, or be prepared for specific scenarios - maybe even train your weaknesses and be like: Okay I know if I get the ball on the back line, I usually lose the ball here, but what can I do to actually keep it in, or do something great with it - I just feel like working on specific scenarios. I would work a lot of my, on my left foot as well, because I'm really right footed - so stuff like that would really help me to just... Yeah, do it a lot of times, so hopefully I could build up like: Okay I can do this - kind of like that thought in my own brain. So when I'm actually in that situation, I just go with it and believe in it instead of – having, - like hopefully the reality, or the virtual reality could be like: Okay I have the thoughts while I'm doing it in the program, and when I'm on the field I'm just going to do it - so like the thought-process from thoughts to action is shorter and you just believe in what you're doing. Does that make sense?

Interviewer: Yeah, that makes perfect sense and that's - no, that's a really good point that, you know, you can like practise what you feel like you're not so good at.

Participant: Yeah.

Interviewer: And also just like improve like, you know, through repetition and succeeding in the virtual environment may make you feel like you can do it on field as well.

Participant: Yeah. Definitely.

Interviewer: Cool. So do you see any like challenges that could come up, or like drawbacks with using virtual reality?

Participant: Drawbacks - is that like negatives it could bring kind of?

Interviewer: Yeah.

Participant: I don't... Like for me the biggest negative would just be if it was a shit experience and then I would just never do it again.

Interviewer: Yeah, yeah, yeah.

Participant: So I wouldn't say it could do anything bad to your game - like it couldn't give you less confidence, it wouldn't be able to like… Do something that's so major that I'm going to be a less better player. Does that make sense?

Interviewer: Yeah, yeah, yeah.

Participant: Like, I don't think it would have any impact like that - it would more be like: Okay, I take the goggles on, I don't feel like this is realistic at all so I'm just going to not do it. Like, it's confusing me more than it's giving me something.

Interviewer: Yeah.

Participant: Yeah, I think that would be the biggest negative if it wasn't working. Like if it was too complicated and you thought more about the technology than actually what you benefit from doing it.

Interviewer: Yeah, yeah – that makes sense. It is hard when you don’t – haven’t tried it, so you don’t know how it feels, but that’s a good point.

Participant: Yeah. Yeah. Yeah, but if it runs smoothly like I can't see anything bad coming from it.

Interviewer: Yeah, yeah, yeah. Absolutely. Do you think it would be easy to use and easy to implement as part of your training?

Participant: Depending on - obviously the technology and the program - I don't know how easy that's going to be. But I would say like - to use for a team like ours, for example, like it would just mean that the club need to buy a set of virtual reality, or have a room accessible where you could do it in. Ummm so I don't think that's hard at all. Like if that program shows to really do something, or can help people - and just even like that little 1%, I feel like that's definitely worth it. And a lot of clubs and teams would take advantage of that. So I don't think - like if it's easy to use the little technology by itself, I would say that a lot of clubs would definitely just get it in and get going.

Interviewer: Yeah. Yeah, absolutely, yeah. I know it is a new club and like you have only been here for a week, but has it ever been discussed like with your teammates - either here or at, you know, your previous club - the use of virtual reality at all?

Participant: No, I’ve never – not before you texted me. I've never heard of virtual reality in soccer. Like, obviously, a lot of FIFA computer games and whatever - like we've all seen that, and…. I wouldn't say like you trained via FIFA, cause obviously it's not you, and it's not that realistic, but it's like... I, I just think it's an interesting thought, but I no one have ever mentioned like training through virtual reality, no.

Interviewer: Yeah, right.

Participant: So that's not normal… Yet.

Interviewer: Yeah, hopefully it will come in… Do you think your teammates would be open towards using it if they had access to it, of course?

Participant: Yeah. Definitely. Like I wouldn't… I think everyone's thought would be right away, like: Oh virtual reality is so fun. Like, let us - like let's get in, let's do it. But I think that everything that can help us, especially maybe female athletes in general, would maybe be more open because we haven't tried a lot of things. So I feel like... It would just be like - start as a more like maybe fun thought and a lot of people would probably just try, kick around, whatever you can do in the program.

Interviewer: Yeah

Participant: But if you actually like sat down as a team and the coach, and you came out to show us and you were really like: You can really benefit from like these things, I think… I can't like - I've only met the girls a couple of times now, but I have like no one in mind that would just be like straight up: No – like no, I don't want to do that. I think that... Like kind of just like us, like obviously I talked a bit to [removed for protection] about it, but I feel like people would definitely be open if it can - sometimes it's those small, less things that can make you better. So why wouldn't you try and give it a go?

Interviewer: Yeah, absolutely. Like it's all the extras. Like you all have the skills.

Participant: Yeah. Yeah, exactly. Yeah. No, that'll be good.

Interviewer: Cool. And how about your coaching team? I'm guessing it hasn't come up, but do you think they would be open towards implementing it, or letting you girls use it?

Participant: I feel like. Girls, or the team - the players would probably be more open right away because it's not our money and not like, yeah, our - or not our planning and whatever, but I would say that… I feel like some coaches - I don't know him that well, my new coach, but I would say some coaches would probably be very open and just be like: Yeah, we want to try everything that makes us better. Like it's a good thing. But I could also see the other side of it, from a coach perspective, that they want to wait until there's actually some results of it helping - to get like actually some… What do you say like - some search in some… What's that word?

Participant: Evidence…

Participant: Yeah, evidence. Evidence of like it actually being like super helpful. So I feel like some people would be like: Oohhh yeah, let like let's give it a go. Why not? We have nothing to lose.

Interviewer: Yeah, yeah, yeah.

Participant: But I can… Yeah, I think there would be some sceptic coaches out there, being a little like: Ooooh let's wait for a couple other teams to try it and then we can jump on board.

Interviewer: Yeah… Which is fair

Participant: So I guess that could a 50/50

Interviewer: Yeah, which is fair… And some coaches are very much like – just go and run around the field.

Participant: Yeah - and if you get like an older coach - older traditional coach, maybe it's more like: Yeah - let's just do what we've always done, like that shit is not going to work. But I think - yeah, I think a lot of people would be extremely positive towards it though. Yeah..

Interviewer: Yeah. Cool. Perfect. Ummm so I'll just sum up everything that we've spoken about

Participant: Mmhmm. Yeah.

Interviewer: And then correct me if I'm wrong, or if there's anything you want to add.

Participant: Mmhmm. Yeah.

Interviewer: But overall it sounds like you're very positive towards the use, and you would implement it, you know, if it was presented to you, if you had easy access to it

Participant: Mmmhmm. Mhhmmm. Yeah, definitely.

Interviewer: And the main things you think that it could be used for - well, there's a lot of things, but like 1V1.

Participant: Yeah

Interviewer: Umm like scenarios and exposure to penalties, but also corners and free kicks - and just practising your drills and shooting the ball in general.

Participant: Mmmhm. Yeah.

Interviewer: And then, you know, gaining greater confidence because you can repeat different situations.

Participant: Situations… Yeah, definitely.

Interviewer: And you don't really have any negatives because you haven't had any experience with it?

Participant: No... Like, yeah, as I said, the only negative would be trying it and then not trying it again, cause you're like: Nah, not for me. Yeah.

Interviewer: Yeah. But you don't think it could like harm your performance?

Participant: No – no damage. I wouldn't say... Nah. I don't know how physical active you would be in the program. Like, I guess there is a little aspect of injury as well, depending on what you're doing, but yeah.

Interviewer: That's the thing - you can't like because you have this headset on, so you can't run around. So it would be like standing - well without this table, like standing here and like kicking a virtual ball in a virtual environment…

Participant: Yeah. Yeah. So I guess like that's still a less possibility of injury there. So that's a good thing there.

Interviewer: Yeah, yeah. Cool. Perfect. Was there anything else you wanted to add, or any questions or anything in regards to virtual reality?

Participant: No, I think that was about it. Unless you want to know more - I'm just interested in trying it as well.

Interviewer: Yeah, absolutely.

Participant: Because yeah, it'll be interesting to see what it does. And I think that it could have a positive effect on some players... Definitely, you would get a fair bit of players that would probably be like: Nah, waste of time, or like: Nah too hard to access, or whatever. But I think… I think it could really move something in people and really give that - I think the confidence aspect is really big… Just getting used to situations, yeah.

Interviewer: Can you think of other ways it could help you?

Participant: Hmmm… I don’t really. Really like how specific can you be - like if, let's say that we started on the [removed for protection] team - could you create a change room environment, as well, could you create everything or is it more just on field?

Interviewer: Like it's - you can essentially create anything, but I would say like mainly on field, because that’s where you can… Like you could create a change room, but I don’t think it’s been done before, I think it’s mainly like on the field to help you improve on field…

Participant: Yeah. Yeah. Yeah, yeah. No – cool. Yeah. I would, I would say something you could practise as well could be like when the whistle blows for the first time - like cause that's always when you're very nervy and like: Oooh I haven't touched the ball yet, so like your first couple of touches on the ball I would say that could be a smart thing as well. And obviously your position, like it's been a lot about having the ball, your position in the pressure -like how are people playing? Yeah, around you as well. How do you position yourself in a game like that.

Interviewer: Yeah right…

Participant: You can't really talk to people in it - can you?

Interviewer: Nah… Well, no, not really, cause there's, you know, still a robot kind of thing, so although everything can be manipulated then it’s like…

Participant: It's more you and your situations.. Yeah, but even the pressure would be good to train... But I think, yeah, I think that's it.

Interviewer: Cool. Perfect. That's really good insights.

**Participant 13:**

Interviewer: Can you start off just telling me what you know about virtual reality?

Participant: Just know kind of like the goggles, gaming... Yeah, bit of like.. Uuummm… Oohhh what do you call it? Thee…. I don’t know now. Like I’ve seen videos and stuff of people like literally living in those worlds, you know so… Umm… Ohh that's annoying I don't know what, what the hell I'm trying to say, but yeah they have basically a different life in there. Can work, live - all that kind of stuff, which is pretty crazy. But yeah I don't, like to be fair know a whole lot only you know that you can essentially play games, be in that other reality.

Interviewer: Yeah, absolutely. Have you had any experience with it at all?

Participant: No, I've never tried it.

Interviewer: No. Cool. Can you think of how it could be used in football training?

Participant: I mean... Just from, you know, chatting initially about this whole project, I think it can be used in so many different ways in football. Umm… You know, and I've just been thinking as well, like it would be awesome resource for training, you know, of course you've got to go out there and run and do things physically and all that - do all that. But you know, just an additional resource to reinforce things. Maybe you wanna - after you've done training, after you've done gym. Maybe just wanna sit in the room, you know, and it can be an animation of something that you guys have been working on training, where people should go, yeah, as you've mentioned, corners, penalties, free kicks… Ummm. Yeah, all that kind of stuff would be awesome just to, yeah, just reinforcing the mind like: Okay in these situations I've got this player that's free, I should be able to move the ball here, move the ball there. Yeah.

Interviewer: Yeah, yeah, yeah. Cool. So can you think of how it could help you improve your performance on field?

Participant: I think just as that additional resource like… It's.. It's putting it in the brain over and over again. So like in a meeting, I might write some stuff down in my notebooks about what the coach has said or things he wants us to do, you know, which is great to sort of look back on, or pre-training the next day. But you know, and then you go out and you train it. And those are the two opportunities that you really kind of have to, either be better or be better at what you're doing or be better at the team plan, right? So you know, if you, if you only have those two options to practise what you're able to do, then the additional resource could be using a virtual reality of, you know, the plan, the people, the field, the positions, movements - all that sort of stuff. So you know, there's, there's also additional extra resources like watching video that - which is what we do, and.. And you know, maybe even just sitting down and having a meeting and talking about it. But I think it would add a huge additional layer of, of exposure and just kind of being able to make it more realistic than sort of you and I sitting here, you're the coach, I'm the player we're talking about what we should do and then you know, it's maybe not until the next morning that you can actually get out there and practise it. So... Yeah.

Interviewer: Yeah. Yeah cool. So through like exposure, repetition…

Participant: Yeah

Interviewer: And just, you know, getting experience with what you're talking about eseentially…

Participant: Mmmhmm.. Yeah.

Interviewer: Cool. Ummm and - if you had access to it - would you be open to implement it as part of your training?

Participant: Yeah, definitely. And I think like maybe some people won't be some people will be, but I think, yeah, if I was... If it was available tomorrow for us to use, I think it would be, yeah, just another great thing that you could, um you know, spen - you do in your spare time before you leave, you go and yeah, sit in the, in the chair and put the goggles on and just go over a couple more things, or right before the game.. You know, you're just going over where should I run in a corner, you know, where should I be on a free kick, where should I be, yeah in this position? I, I really like the thought of just that additional resource for that reinforcement. Yeah.

Interviewer: Yeah, absolutely. Cool. So in what areas do you think it would be most beneficial?

Participant: I think it would be most beneficial… Ummm. I think it would - like I don't know if there's a more - like one that's most and one that's least, I think overall it would just help in every aspect. For me personally, I'd probably say practising attacking corners, practising defensive corners would be awesome. Because I actually really find that like my brain capacity from heading the ball could potentially be really affecting me later in life and when we just practise one or two, just to get the movement right and it's like rock hard, you know, it's, it's really affecting cognitively my brain. Like I can feel it over the amount of years that I've been playing now. So you know if we could practise that without actually the impact. Amazing.

Interviewer: Yeah. Yeah, yeah, yeah.

Participant: Yeah, really amazing I think for me personally. And you know, that doesn't actually need… For example, you can't, you can't practise through that - running and chasing a player and doing a slide tackle, or timing your tackles - like you can't practise that cause that's something physically, but you can practise - yeah, your movement, where you need to run, trying to evade a defender, or, or defensive corners.. Trying to, you know, keep them there or you know, whatever - I just think that those kind of set play ones would probably benefit, yeah the most. Yeah.

Interviewer: Yeah, absolutely. So like different movements that you do throughout the game would be helpful?

Participant: Mhm. Yeah.

Interviewer: Cool. Do you think it would be easy to implement as part of your the training you're doing right now?

Participant: Mhm. Definitely. I think with technology and the advancement of like being professional

Interviewer: Yeah

Participant: Especially like here in Australia like I've been at this club before and I've been at many other clubs and sort of each year you sort of see a little bit of progression, if you're lucky.

Interviewer: Yeah

Participant: This year it's been sort of really good access to all these high performance facilities. There's nothing really that extra that we need, that we normally have. This is something new that would come in, you know, and advance. I think training and just being better athlete in general and a better teammate, so I think that… So what was the question again at the start?

Interviewer: Whether like - how you think could…

Participant: Or how, if I would, if I would do it…

Interviewer: If you would use it…

Participant: Oh yeah, yeah, yeah. So yeah, I do. I absolutely would.. Just because, yeah, I wanna do everything I can to be better.

Interviewer: Yeah.

Participant: And I know that every other athlete who's playing at this level wants to do that as well. So definitely. If it was an extra resource, we could use I have no doubt that a lot of people would wanna - at least try it, you know - if it doesn't work, doesn't work.

Interviewer: Yeah, yeah, yeah

Participant: But at least try it, yeah.

Interviewer: Yeah, absolutely… Umm do you foresee any like challenges or drawbacks with using this?

Participant: Ummm… Maybe just the fact that you can only do something that's pre-programmed.

Interviewer: Yeah

Participant: Maybe not every possibility can be in there, which is obviously normal, but I think that's a small challenge really, because if you can put as many as sort of you can in there, you'll probably be better off dealing with 80%, or 90% of situations and handle the 10%, you know that you can't sort of do around that.

Interviewer: Yeah, absolutely.

Participant: Yeah. Yeah. So, but yeah - other challenges maybe financially. Like - is there, is this costing a lot of money? A lot? I know a lot of clubs don't have a lot of money, or especially for their women's programs, maybe they give it to the men, but that's just the daily battles that we face. So yeah, maybe potentially financially, I don't know what a program or something like this would cost, but if money wasn't an issue, I don't think that would be a challenge.

Interviewer: Yeah

Participant: Umm… Maybe just your basic technology challenges - crashing and batteries running out, like that sort of thing that we deal with every day with phones and stuff. Yeah.,

Interviewer: Yeah, yeah, yeah. Yeah cool. So overall, sounds like you are very positive and open towards the use of this technology?

Participant: Mmhm. Yeah.

Interviewer: Cool. So you also mentioned that like you could put the headset on just before going into a game or a training session.. Can you talk about how that could help? Like do you mean like mental preparation for a game or like in what way?

Participant: I think more so in a way like if I was to just take the example of going in - just before a game.

Interviewer: Yeah

Participant: Maybe the morning of, I don't know if I would wanna do it while I'm in the change rooms getting ready, literally like an hour before you go and warm up.

Interviewer: Yeah

Participant: I think that would be too much. But yeah, just, just about using it sort of before - on the day of the game it would, it would just be that repetition reinforcement: Okay, do I have a question about where I need to be? Have I forgotten… You know, normally in the change rooms, we would put up sheets of paper on the wall of set plays or things that we sort of need short dot-point reminders on, and you know you would sort of stand there for 10 minutes, read through them all - that could act as something like that. Doing it with a bit more realism.

Interviewer: Yeah

Participant: And a bit more detail, a bit more information, but... Yeah, I, I think that I, yeah probably would avoid doing that, yeah, about now before the game because it’s, it is a lot of overload and it's like - you still have to worry about how you're performing and what you're gonna do and how you're prepared and what am I gonna do, what I'm gonna focus on this game like… I think it would be a good tool to use sort of like during the week rather than like on a game day? Yeah, for sure.

Interviewer: Yeah, yeah. Yeah, absolutely. Has the use ever been discussed among your teammates? It's a new club, but like with your teammates from previous clubs at all?

Participant: Nah. No, never. Nah, I've never heard of like incorporating this kind of technology. No.

Interviewer: No. Do you think the players and your team would be open towards the use?

Participant: Yeah, definitely. Yeah. As I mentioned like we're all wanting to be better everyday and we all want great facilities and, you know, you know we don't want to be in a dirty change room. We wanna be in nice clean, like professional change room or, you know, a gym as well... We want pools, we want, you know - we want all the 1%s to be able to recover and perform, and I think that's it's an additional 1% resource. So yeah I think a lot, if not everyone would be keen.

Interviewer: Yeah absolutely… And how about the coaching team - do you think they would be keen?

Participant: Oohhh they would love it. Yeah, they would love it, especially our coach now. He's a teacher background and he's just, like, very meticulous with his planning and his PowerPoints and his scheduling and his, yeah, timetables and colour coding and all that kind of stuff. So he would love the.. Umm. The technology, first of all, he'd love the idea of, of it being that extra resource and potentially getting 1, 2, 3% better. Yeah.

Interviewer: Yeah, right. Yeah, that's cool. So overall it sounds like everyone in the sport and a new club would be open towards using it. Um and the main benefits you see are like, you know, practising heading drills, but also just drills in general. And like different situations you may be exposed to, and then just getting that realistic experience rather than just, you know, talking about it or training - then you can train outside of regular training. Is that correct?

Participant: Yeah, I think it would be pretty hard to replicate, but maybe not… I don't know what the extent of the technology is, but to replicate like real gameplay, where you actually see your 11 in front of you, or, or your 9 because the keeper is behind you and the other 11 of the other team in front of you moving at realistic game pace and game-decision movements, I, I don't even know if that's possible - if that is then it would be brilliant.

Interviewer: Yeah

Participant: But definitely your penalty, definitely your corners, pre-program free kicks, all that sort of stuff I think would be awesome. Yeah.

Interviewer: Yeah. Yeah. How do you think it could help - so like, do you think just through the exposure, or like improving your skill set, or – can you talk about how?

Participant: Um not improving skill set because that's all about physical touch on the ball, positioning, moving the hips, you know, like actually moving the body

Interviewer: Yeah

Participant: However… More confidence in what you're going to be able to do, more confidence in the spaces that should open up, more confidence in what your role is.

Interviewer: Yeah

Participant: Because there's nothing worse really when you are on the ball and you're like - where's my players? Or what am I supposed to do in this situation? You kind of panic, you're on fight or flight mode, and you just kind of like have to make decisions on the spot, so I think with that repetition, with that continuous exposure of, of what should happen. Yeah, it gives you a better chance to be prepared for that 90% and then that 10% of yeah the game that might come at you differently to what you would expect, or what you could do that training for is sort of like still prepared to be able to handle those moments, and hopefully there's not too many in the game. Umm.. Yeah.

Interviewer: Yeah. Yeah. That's really good point. So there's like, you know, getting used to different situations and maybe like improving your scanning and like what decisions to make under the pressure.

Participant: Yeah. Yeah, definitely.

Interviewer: Cool. And the biggest barriers you see like the technology issues and then like affordability and maybe a bit of a accessibility –

Participant: Yeah

Interviewer: And then just like, you know, because you haven't tried it, it's hard to know whether it will actually work – is that right?

Participant: Mhm. Yeah, but you know, yeah, you don't know until you try, but you know, sort of if what I'm seeing in my brain about how it could work, I think it, it really could.

Interviewer: Yeah.

Participant: If this isn't invented yet, then it will be very soon, of course. And.. As the years go on, while it's here, existing, then the better it will become, you know, of course, like anything.

Interviewer: Yeah

Participant: So, you know, it's pretty exciting to think that there could be something that completely mimics real-game life stuff. So yeah.

Interviewer: Yeah. Yeah cool. Perfect. Do you have any other like thoughts about virtual reality in football training? Or any questions…

Participant: Ummm.. Only just that it's, you know, it's probably not even limited to football, you know, there's probably so many other sports that as well that can do exactly the same thing.

Interviewer: Yeah absolutely.

Participant: If it's not already there so... Yeah, There's my thoughts. Yeah.

Interviewer: Yeah. Perfect. And overall you’re very positive towards it, it sounds like?

Participant: Yeah, definitely for sure. And I would, yeah... As I said, love to try it and love to be hitting less balls with my head.

Interviewer: Yeah. No, that's good

Participant: Because I know that I'm gonna be.. I just have this feeling I'm gonna be suffering later on in life.

**Participant 14:**

Interviewer: Can you start off just telling me what you know about virtual reality?

Participant: Um so yeah, no stuff about virtual reality in general - not specifically what to do with sports and just, yeah, like very basic kind of like with the kind of headset on and then you can, you know, simulate different events or things or whatever it's used for, I guess in sport you can simulate, you know, being on a pitch um and yeah, just trying to immerse yourself into specific situations to replicate whatever it is. So that's kind of the general sort of idea I've got about it at the moment.

Interviewer: Yeah, cool. Nah that's really cool and that's very good knowledge when you haven't had any.. I don't know - have you ever had any experience with it outside of sport?

Participant: Never personally, but obviously like I've seen videos and know people that have used it so…

Interviewer: Yeah, yeah, yeah. Cool. Um so thinking about how it could be used for football specifically - can you thought talk about how you could see it being beneficial for football training?

Participant: Yeah, I guess it comes down to like replicating any sort of situation so… Whether it's a training situation, a match situation, anything specific, I guess can be replicated through the.. Um yeah simulator, if that's the way to call it. Uum and yeah, just kind of putting yourself in there to know how you're gonna respond to certain situations uummm so then come on, match day, you're prepared, or you've at least seen, you know, similar images or scenarios before. So I think it's something that's maybe not been tapped into with sport a lot. Um obviously, being in that environment now, it's never ever been sort of brought up or used, or even mentioned. So I think it's something very new but with the way like sport and just the world in general is going with technology, I think maybe in the future it could play a part. How big that part is, I'm not sure because it's still so new, but yeah, I think it's something that I'm probably gonna have to come across in my career at some stage.

Interviewer: Yeah. Nah, that's really good insight and very interesting... Um so you spoke about like, you know, getting exposure to situations that may come up during a game or even during training. Can you talk about some of the specific like scenarios or skills you think that you could um practise in a virtual reality simulator?

Participant: Uuhm I think the big one would be more like tactics and obviously you can run through tactics through training sessions or you can try and replicate it as best you can, um but it's kind of hard when you can't do it with the actual opposition. So having the VR reality to mimic the exact opposition, the exact player, the exact personnel. Um yeah, through the technology, however that would be set up to actually, yeah, practise your tactics or practise what you're gonna do against that specific opposition I think would be a big help. Um cause yeah, you can kind of replicate it to a certain extent internally just with your team and with making fake scenarios and that in training. But in terms of actually seeing it and being on the pitch and yeah - seeing it through your own eyes and actually being out there in your specific positions, I think would be a big difference. So then it's just nothing that you haven't seen or it's not as foreign come the game and then you're more comfortable um to make a better decision, I guess.

Interviewer: Yeah, absolutely. Nah, that's really good insight to use it for like that tactical training. Are there any other areas you could see it could be beneficial in terms of your football performance?

Participant: I think just with anything to be fair. Um like I mentioned, the tactic would be, you know, a really good specific thing, but I think anything.. Ummm, yeah, if it's more from like a skill type based, if it's more from like an individual or like a… Yeah more like a personnel type base to kind of replicate what you'll be up against on the weekend, whether that's a team or a specific player or whether it's a specific stadium or whatever it may be I think there's nothing really off limits with it, just from thinking and not really experiencing it, I think with technology you can kind of replicate anything, so I presume you could, yeah, replicate or give scenarios for absolutely anything got to do with you know, the sport.

Interviewer: Absolutely. And that really is one of the major benefits of virtual reality - that you can individualise the training and make it like, you know, for your team, but also for you as an individual versus, you know, someone who's a goalkeeper, like you could do it from the perspective of the goalkeeper and what training they need. So you mentioned it hadn't really been spoken about, but if you had access to it, would you be open to using it as part of your training?

Participant: Yeah, I think for sure. I think us athletes are always looking to find that bit of an edge or something that's gonna enhance our performance. Obviously, as a team first, to try and win games, but also individually to try and perform your best. And yeah, like I said at the start, I think it’s just something that has not been mentioned or ever available because it is so new. Um but I think yeah, myself 100% would want to try it at least and I think from the broad knowledge I have about it, it would be something that… You know, if you were to try and it wasn't for you, you're not really losing anything. But I think there's only gains to be made from it and only positive impact to be made from it. So yeah, 100% would give it a go.

Interviewer: Oh yeah, that's um excellent. Um so um you mentioned a few, like you said nothing is really off-limits and then you touched on like, you know, individualised training, um tactical training and exposure just in general. So what would you find would be the most beneficial in a virtual reality simulator, do you think?

Participant: Ummm in sort of specific sort of thing got to do with the sport like that I'll find useful?

Interviewer: Yeah, in what areas do you think it could benefit you with the most?

Participant: Yeah, I think it's more from, like I said at the start the tactical. I think, yeah, like I said, you can replicate or watch as much video or do enough research analysis on the tactical and your opposition and that. Um but until you actually get out on that pitch on the weekend and you're in a stadium with people and yeah, you just kind of not see it for the first time, but you're in that moment for the first time, it is very different from everything you've done throughout the week, or your years of experience until you actually get out there. Um so I think the virtual reality would help me probably the most. And also being a midfielder in soccer, so being in the middle of the pitch the most, there's the most traffic going around, the most - you're seeing the most different scenarios, the different images in your head because you're always involved in the play. So trying to replicate that and get as much um training and mental training of seeing the different images, different scenarios would help me the most as a midfielder.

Interviewer: Yeah, that's very good insight. Thank you. So it does sound like you overall are very positive towards the use of virtual reality. Is that right?

Participant: Yeah, like I said, without ever experiencing it, I think just… Yeah, it would be silly not to, yeah, be open to it. Especially, I'm someone that wants to try and always improve or find new ways to get that edge, as I was saying before. And I think without obviously speaking for anyone else, majority of professional athletes, I think in any sport or field would be open to it.

Interviewer: Yeah, absolutely. So of course it is hard when you haven't experience it, experienced it yet. But can you see - like are there any drawbacks or challenges you think might come up with the use of virtual reality training?

Participant: Yeah, it would just… Like, yeah, without ever using it, it's just like I think more than like the logistical part of getting it set up. Where would you implement it in a, let's say, a professional training base, or how would that work, and how would you incorporate that into your training? Because obviously you train a lot and obviously on the field you're doing a lot, off the field recovery. How would you incorporate that sort of into your schedule and how would you do it, you know, is it something that's done individually or can you somehow do it, you know, with a couple of players at a time or… How would the coaches use it, maybe within a training session? Or is it something that has to be done, you know, completely away from the training, you know, maybe on… Yeah, like your spare days or, um… So kind of like the logistical part of it, I would say. But in terms of like the actual use of it and the actual - what it can be used for, I don't think there's any real limitations on that side of it.

Interviewer: Yeah, that's good. So mainly just like, you know, the planning of the of it and…

Participant: Yeah

Interviewer: Yeah, cool. So you mentioned it hadn't really been spoken about, you know, in your training environment. How do you think your teammates would be… Um what do you think their attitudes to the use of virtual reality would be?

Participant: I think the majority would be positive, like I said. Umm I think maybe with the younger sort of players. Obviously I'm only 22 and technology's been a big part of my life, just in general. Maybe some of the older players who are a bit more old fashioned or don't quite believe in it as much might struggle to find the benefit in it. But I think as a whole… If it was something that was explained to them and they had a clear outline of how it would work, how it would benefit, how easy it is to use and every, everything put in front of them so they didn't have to, you know, come to make their own conclusions in their head, I think everyone would be open to it if it had some benefit.

Interviewer: Yeah, absolutely. Um so just about like providing the like evidence or like, and then of course how to use it and what?

Participant: Yeah. I think with a lot of… I think in general with a lot of people, especially with athletes, we like to know that something's proven.

Interviewer: Yeah.

Participant: And obviously, there's years and years and years of evidence with a lot of things, whether that's physical, mental training habits and that sort of thing. And with it, just this, um this sort of stuff being so new, I think, yeah, if it was all kind of compiled into one thing or explained to someone or if there was a session for those that were unsure and they maybe found the answers that they had in their own head. That would make it, yeah, a lot easier for some people to come around to it.

Interviewer: Yeah. Yep, cool. That makes sense. What about your coaching team - do you think they would be open to their athletes using it?

Participant: Yeah I think for the most part. Again, I think it'll come back down to maybe the older sort of old-fashioned coaches, maybe not so much. Um, yeah, just older sort of people in general are kind of against technology in a lot of things.

Interviewer: Yeah.

Participant: But again, if it's something that.. Not time consuming, not cost consuming, nothing that's out of, out of the ordinary or, you know, too much effort for - to go through with whatever it is I think, yeah, that would be open to it. And yeah, if it improved the team's performance and it helps them get messages or coaching points across, or make us perform better on the weekend, I think, you know, they'll be stupid not to be on board with that, you know.

Interviewer: Yeah, absolutely. Um so just all about like you know, providing as much information and evidence as possible for, you know, athletes, but also the coaching staff?

Participant: Yeah, and I think that's like in any sort of field in life is, I think people will be very hesitant at the start until you kind of get and kind of until it's proven that you know it does work, or it is worth at least being open to it. So I think that's kind of the main thing around, whether it's players or coaches is just, yeah, being provided with some evidence or some information that supports it being, you know, a positive thing.

Interviewer: Yeah, absolutely. Um so if you had access to a virtual reality simulator, so you know, of course the headset and then like a software, you think it would be easy to work out if you sort of had it explained to you?

Participant: Yeah, I think for the most part, if it, yeah, obviously we were showing how to use it. Um it would be pretty easy, without ever using one myself, but when I'm seeing other people use it, or videos, it seems pretty straightforward. But yeah, as long as it was explained to us and you know, we got to test it out first I think there's no reason why anybody couldn't pick it up.

Interviewer: Yeah, cool. And do you think it would be easy to incorporate into your training routine? So of course, you know, the physical training would always be there and it would never replace that. But just as an addition to everything else that you do?

Participant: Yeah, I think for sure. And then it would just, yeah, be about trying to find the best time to use it and when's the most effective time to kind of incorporate that into the obviously physical training and that.

Interviewer: Yeah.

Participant: And just, yeah, how that impacts yourself physically and mentally, everyone, every player, every individual would be different on that. Some would be able to do, excuse me… Some would be able to do more or less, so just kind of trying to find everyone's, you know, kind of sweet spot with it I think would be the main thing.

Interviewer: Yeah, absolutely. And of course it is very hard to speak on it when you haven't tried it, but it's good to hear that you have some initial thoughts about how it could be beneficial. Um so I will just summarise everything that we have spoken about and if I've missed anything, or you have anything to add, please just add it. But overall it does sound like you are very positive and open towards using virtual reality in football training. Um and you sort of see it, like there are many benefits and like there, there are no limits really, but the biggest limits, or like you know the biggest benefit you see are tactical training, exposure to situations that you may encounter in games and just exposure to like, you know, the like the opposite team, the stadium and other factors. Um and then the biggest barriers you see are like the logistics of it and then, you know, just figuring out how to implement it so it, it benefits you the best.

Participant: Yeah, I think that pretty much sums it up. The only, not sort of limitations, but the only, yeah question marks, because it is something so new, just how does that look in training and I think… Yeah, until it's kind of tested consistently and at a high sort of level, you know, at a professional environment those answers maybe can't be given. And whether it is being tested which I'm sure it is you know in a lot of bigger clubs, I'm sure in Europe where the financial side of it doesn't, you know, hold teams or people back from using it, I'm sure there is like evidence but that would be the main thing. And then, yeah, in terms of the actual using the headset and the VR system, I think yeah, there's nothing really negative about it. Um, yeah, for some people it won't work. But yeah, in my opinion, I think there's only positives that can be taken from it.

Interviewer: Yeah, absolutely. So um just touching on that again. You don't think it could negatively impact your performance?

Participant: I don't think so. I think - obviously I don't think physically it's gonna harm you, mentally, it's not.. Obviously it's gonna stimulate your mind, but it's not gonna push you to the edge to say. Um and yeah, it could only really benefit you. And if it's someone tries it, or a player tries, or a coach tries it and it's not for them, like it's not gonna, it's not gonna be the end of the world or impact their performance on that given week, you know. Um it's not like you're running 10 kilometres and physically you might be impacted. You know, it's just trying it, if it doesn't work, then yeah, it's not gonna affect you. So there's only really positive that I can see you can gain from it.

Interviewer: Yeah. No, that's very good. Um… Cool. Just wanna come back to one point you said, so you said that as a midfielder, there's a lot of traffic going around you. Do you think it could help you improve sorta like your scanning and, you know, like the way you have to always look front, left and centre um and with decision-making?

Participant: Yeah. Like I said, being a midfielder, and always being in the middle, that's probably the main one. And yeah, like I say, in training you can replicate that, but you're training against your own team.

Interviewer: Yeah, yeah

Participant: So obviously you can create teams, or you can create - if you're on one team, you can create the other team to, you know, replicate what the opposition might do, or a specific individual. But it's still not that actual personal, that actual team, or you know.. And until that actual person is in front of you and you can see it, and you can - I'm sure with the VR reality you can replicate it pretty much, you know, 100 % um even to the smallest detail of how big that person is. So when you're actually on the pitch, is it someone small, big, like you can, yeah, pretty much replicate that to the, to the specifics. So I think yeah, with the scanning and being a midfielder, just getting as much information and training your mind to see every little detail on the pitch which people probably don't see when you're, you know, as a fan or someone that outside, which is, you know, completely fair enough - you don't understand every little detail, but as an athlete, every minute detail matters. And if you can, at least expose yourself to that, it means come on the match day when you're in that exact same scenario. Then of course you're going to be more comfortable, which means you're going to make a more better, well calculated decision of whatever it is. And then that usually results in, you know, a more positive outcome and whatever that outcome is, for a midfielder, it might mean I'll make a good pass or… Um I score a goal or you know, I help my team do something in our tactical stuff or whatever it may be. Obviously there's a whole range of stuff, but I think, yeah, just being exposed to it and then come match day when there's all the factors of the stadium, the fans, it's loud, maybe you're tired. Well, your mind's already seen this picture, so you know exactly, you know, what to do or what to expect.

Interviewer: Yeah, that is very good insight. Thank you so much.

Participant: That’s okay.

Interviewer: So just wanna finish up um talking about virtual reality. You just gave a lot of very good insight, but do you have any additional thoughts about the use, the benefits, the potential barriers or anything that we haven't touched upon you want to add?

Participant: Not really. I'm just more thinking, yeah, like how it would kind of work trying to like relate it to like my training… How you would best use it? Cause it's not really, yeah, like a limitation or negative. It's just more like, yeah, my head like a brainstorm of how that would look. And I think it could like, 100% be used in, I think, professional athletes in general. Cause, like I said, it's nothing physical or mentally draining where it's gonna affect your performance. It would just be more around, yeah, like I said, the logistical side of it and how you actually set that up and incorporate that into your training. Which I think, yeah, I think there's definitely a way and I think it just requires, you know, trial and error, but.. For sure, I reckon in the next few years that's something that maybe will become normal in professional training.

Interviewer: Absolutely. I think it's just about incorporating it into, you know, it's a very good mental tool. So like on the pitch, it's so like physical, but to have it as an additional thing so you can, you know, practise those scenarios that you may encounter in the game, but it will not be as physical on your body.

Participant: Yeah

Interviewer: Um but of course there's so much more research to be done in what's the most optimal way of using it as a professional athlete.

Participant: Yeah, sure. I think that's, I think that's probably the exciting bit - is just it's still so new and so raw, so yeah, as long as - like the idea is right and the idea is there, it's just yeah, about taking it a step further, I think.

Interviewer: Yeah, absolutely. Cool. Do you have any other thoughts about virtual reality?

Participant: Nah. Not of top of my head anymore.

Interviewer: Cool. Perfect. And we can always come back to it - if you want to.

Participant: Sounds good.
